# Gene expression profiling gut microbiota in different races of humans

**DOI:** 10.1038/srep23075

**Published:** 2016-03-15

**Authors:** Lei Chen, Yu-Hang Zhang, Tao Huang, Yu-Dong Cai

**Affiliations:** 1 School of Life Sciences, Shanghai University, Shanghai 200444, People’s Republic of China; 2 College of Information Engineering, Shanghai Maritime University, Shanghai 201306, People’s Republic of China; 3 Institute of Health Sciences, Shanghai Institutes for Biological Sciences, Chinese Academy of Sciences, Shanghai 200031, People’s Republic of China

## Abstract

The gut microbiome is shaped and modified by the polymorphisms of microorganisms in the intestinal tract. Its composition shows strong individual specificity and may play a crucial role in the human digestive system and metabolism. Several factors can affect the composition of the gut microbiome, such as eating habits, living environment, and antibiotic usage. Thus, various races are characterized by different gut microbiome characteristics. In this present study, we studied the gut microbiomes of three different races, including individuals of Asian, European and American races. The gut microbiome and the expression levels of gut microbiome genes were analyzed in these individuals. Advanced feature selection methods (minimum redundancy maximum relevance and incremental feature selection) and four machine-learning algorithms (random forest, nearest neighbor algorithm, sequential minimal optimization, Dagging) were employed to capture key differentially expressed genes. As a result, sequential minimal optimization was found to yield the best performance using the 454 genes, which could effectively distinguish the gut microbiomes of different races. Our analyses of extracted genes support the widely accepted hypotheses that eating habits, living environments and metabolic levels in different races can influence the characteristics of the gut microbiome.

Microorganisms are often considered to be small, single-celled or multi-cellular life forms that can only be observed via microscopy^1^. However, despite their size, microbes have been established to be critical mutualists that can act to maintain the stability of human physiology, especially in the intestinal tissues^2^. Since the development of novel sequencing techniques (metagenomics sequencing techniques), a new concept termed the “microbiome” has been established and has allowed comparison and study of the microorganisms in the intestine, or the gut microbiome^3^. The gut microbiome reflects variation in the microorganisms that reside in intestinal tissues, the composition of which shows strong individual specificity and may play a critical role in the human digestive system and metabolism^4^.

It is known that the digestive system is the major site in the human body where food is digested and absorbed. Such processes in the digestive tract are important in humans as they directly affect health and may even be related to life span^5^. However, the efficiency of digestion depends not only on specific and powerful enzymes in the human digestive tract is but also associated with micro-organisms that colonize the same tract, especially in the intestine^6^. Therefore, our health status can be maintained because of the activities of both humans and the symbiotic bacterium found in and around our bodies. Recently, several publications have established the concept that the diversity and stability of our symbiotic bacterium in our digestive tract may protect us from several diseases, such as obesity, cancer and even mental disorders^7^,^8^. The diversity of our gut bacteria may be advantageous. To measure gut microbiome diversity and stability, gene expression profiling of fecal samples may be the most direct reflection of the gut microbiome environment^9^. Without separating and culturing gut micro-organisms, such methods can also reduce human errors and biases. Moreover, gene expression profiling can be used to relatively quantify the expression of specific proteins and functional factors. It can also be used to further evaluate the significance of each functional gene, which may make it an accurate and convenient method for general use^10^.

Abnormal alterations in the gut microbiome can precipitate several diseases; however, even in normal individuals, the gut microbiome still shows great diversity, which may be associated with genetic and environmental factors. Eating habits represent the foremost factor that can affect the composition of the gut microbiome and may further influence our health over a long period of time^11^, giving rise to the aphorism “We are what we eat.” The term “eating habits” encompasses what we eat and the quantity and frequency of food intake, which may also contribute to health maintenance^12^. Moreover, genetic or environmental factors such as gender, age, race can also influence the diversity of the gut microbiome^13^. Recently, differences between gut microbiome from various countries have been revealed^14^. However, differences among various races have received less attention. Such diversity may be induced by differences in eating habits, culture and genetic variations and could reflect the specific function of a gut microbiome found within a given race. Therefore, the specific characteristics of the gut microbiome may represent a useful marker that could be used to cluster people of different races in order study how gut microbiome diversity is established.

Herein, based on gene catalog profiles of the intestinal gut microbiome, we investigated the gut microbiomes of different races. According to the gene profile database of fecal samples obtained from individuals of different races (e.g., Asian, European and American races), we adopted some feature selection methods (minimum redundancy maximum relevance (mRMR) and incremental feature selection (IFS)) and four machine-learning algorithms (random forest, nearest neighbor algorithm, sequential minimal optimization (SMO), Dagging) to analyze the data. Based on the results, the SMO was the best one to identify key differentially expressed genes that may represent optimal functional genes that could reflect differences among different races.

## Materials and Methods

### Materials

The expression levels of 9,879,896 gut microbial genes in 1,267 samples of three different races, which included 139 Americans, 368 Chinese and 760 Europeans^14^, were retrieved from http://meta.genomics.cn/metagene/meta/dataTools. Each sample was RNA-sequenced and represented based on the expression levels of the 9,879,896 gut microbial genes. The goal of this analysis was to identify the most discriminative gut microbiome gene set that was differentially expressed among individuals from different races and investigate the differences in the human gut microbiome caused by food, lifestyle, race and other factors.

### mRMR method

An obvious prediction is that many genes encoded by the gut microbiome will differ between individuals but not be related to differences in race. Some genes may show strong associations, whereas others may not. Thus, it is necessary to use feature selection methods to analyze fecal samples and identify the genes that are the most important. The mRMR method, which was proposed by Peng *et al.*^15^, is a popular feature selection method that has been widely applied to the analysis of various biological problems^16^,^17^,^18^,^19^. In their method, two outstanding criteria were proposed—Max-Relevance and Min-Redundancy. The Max-Relevance criterion aims to select features that have maximum relevance to sample class labels. These features also provide the greatest contribution for purposes of classification. The Min-Redundancy criterion attempts to select features that have minimum redundancies. If the classification ability of a feature is covered by another feature, then the former feature will not be selected by the Min-Redundancy criterion. Accordingly, for a given dataset, two feature lists can be produced using the mRMR method—the MaxRel and mRMR feature lists. Specifically, the MaxRel feature list sorts all features according to their relevance to sample class labels, which is defined to be the mutual information (MI) of a feature and the target variable,


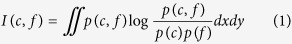


where *c* is the target variable representing sample class labels and *f* is another variable representing the values of all samples under a certain feature. A feature with a high MI value receives a high rank, whereas a feature with a low MI value receives a low rank. The mRMR feature list sorts features using both Max-Relevance and Min-Redundancy criteria. The rank of a feature in this list is determined by its relevance to sample class labels along with redundancies to features listed above a given feature. In the present study, these two feature lists were formulated as follows:


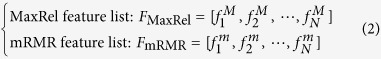


where *N* represents the total number of features investigated. The mRMR method can be accessed from the following website: http://penglab.janelia.org/proj/mRMR/.

### Machine-learning algorithm

As mentioned in Section “mRMR method”, the mRMR method provides only two feature lists. To extract key features, a machine-learning algorithm should be employed. In this present study, we tried four machine-learning algorithms: random forest, nearest neighbor algorithm, SMO, and Dagging, and selected the optimal one.

#### Random forest

Random forest was initially proposed by Leo Breiman^20^ and is an ensemble classifier that integrates several decision trees. Each of the decision trees for a given dataset with *N* samples is constructed according to the following steps:

1. Randomly select *N* samples from a given dataset, but with replacement, *i.e.*, a selected sample is not removed from the dataset and may be selected again. These *N* samples are used to construct a decision tree.
2. Set an integer *m* that is much smaller than the total number of features. To expand the tree at node *v*, *m* features are randomly selected from the total list of features. The optimized split on these *m* features is adopted to split node *v*.
3. Each tree is fully grown without pruning.

For a query sample, each decision tree should yield its predicted result. The predicted result of a random forest method integrates these results by majority voting, *i.e.*, the class that receives the most votes is the predicted result of the random forest.

#### Nearest neighbor algorithm

Nearest neighbor algorithm is one of the most classic classifiers. Although it is simple, it can yield good performance in some cases. Given a test sample, its distance to each sample in the training set can be computed, thereby finding the training sample with the minimum distance to the test sample. Its class is assigned to the test sample as the predicted result.

#### SMO

SMO is a type of support vector machine (SVM) trained by the John Platt’s sequential minimal optimization algorithm. The optimization problem of this type of SVM is always broken into a series of the smallest possible sub-problems. And they are solved analytically. Similar to ordinary SVM, pairwise coupling was applied to tackle multi-class problems.

#### Dagging

Dagging is a type of meta classifier, which builds multiple models and integrates them by majority voting. For a given training set, it firstly constructs a number of subsets from it satisfying that any two of them have no common members. Then, a selected learning algorithm is trained on these subsets, thereby building a number of prediction models. For a test sample, these prediction models would produce their predicted results and the final predicted result is the class receiving most votes.

Weka^21^ is a popular software suite that collects several widely used machine learning algorithms. It contains four classifiers, which are termed RandomForest, IB1, SMO, Dagging, respectively, which implements the four machine-learning algorithms described above, respectively. For convenience, they were directly adopted one by one as the basic machine-learning algorithm to extract important features and to build an optimal prediction model. Notably, they were all executed using their default parameters.

### Cross-validation method

The ten-fold cross-validation method is a popular cross-validation approach that is often used to examine the performance of prediction methods. In this method, a given dataset is equally and randomly divided into ten parts. The samples in each part are selected as testing samples to test the classifier that is trained by samples in the other nine parts. Thus, each sample is tested only once. Herein, it was adopted to examine the performance of different prediction models.

### Accuracy measurement

As mentioned in Section “Materials”, all samples were classified into three classes. To evaluate the performance of a certain prediction model, we can calculate the accuracies for three classes and overall prediction accuracy. However, none of them can accurately evaluate the performance of the prediction model because the dataset was an imbalance dataset, in which the European samples were more than five times as many as the American samples. For a two-class classification problem, the Matthews’s correlation coefficient (MCC)^22^ is always used to evaluate the performance of a prediction model because it is a balanced measure even if the classes are of very different sizes. In 2004, Gorodkin^23^ proposed the MCC for multiclass case. Here, we employed it to evaluate the performance of various prediction models. Its brief description is as follows.

For a classification problem with *n* samples, denoted by *s*_1_, *s*_2_, ⋅⋅⋅, *s*_*n*_, which involve *N* classes encoded by 1, 2, ⋅⋅⋅, *N*. Based on the class of each sample, a matrix *Y* = (*y*_*ij*_)_*n*×*N*_ is constructed in the following manner: *y*_*ij*_ = 1 if the *i*-th sample is in class *j* and *y*_*ij*_ = 0 otherwise. The predicted results of a prediction model can be summarized as another matrix, denoted by *X* = (*x*_*ij*_)_*n*×*N*_, where *x*_*ij*_ is set to one if the *i*-th sample is predicted to be in class *j*, otherwise it is set to zero. The covariance function of matrices *X* and *Y* is computed by


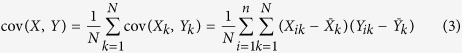


where *X*_*k*_ is a vector consisting of numbers in the *k*-th column of *X*, *Y*_*k*_ is a vector consisting of numbers in the *k*-th column of *Y*, 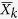 is the mean of numbers in *X*_*k*_, and 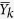 is the mean of numbers in *Y*_*k*_. Then, the MCC for multiclass case can be calculated by


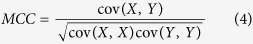


In fact, MCC for multiclass case can also be computed by the following formulation, which was reported in Jurman *et al.*’s study^24^,


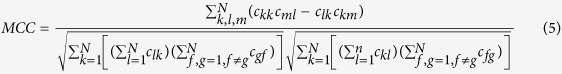


where *c*_*ij*_ is the number of samples in class *i* that is predicted to be in class *j*. For convenience, we simply used MCC to represent the MCC for multiclass case in the rest parts of this study.

### The IFS method

The rank of a feature in the MaxRel feature list indicates only its single contribution to a given classification. The combination of some of the top features in this list does not always represent an optimal choice because redundancies exist among them. Considering this point, the mRMR feature list represents a better choice. The IFS method uses the mRMR feature list and a basic machine-learning algorithm (*e.g*., the random forest, SMO, *etc*.) to extract an optimal combination of features and to build an optimal prediction model. A brief description of this method is as follows:

1. Use the mRMR feature list 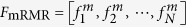 to construct the *N* feature sets denoted by *F*_1_, *F*_2_, ⋅⋅⋅, *F*_*N*_, in which 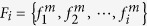, *i.e.*, *F*_*i*_ consists of the first *i* features in the mRMR feature list.
2. For each constructed feature set *F*_*i*_, samples in a dataset are represented by features in that set. A basic machine-learning algorithm is then executed on these samples, and its performance is evaluated by ten-fold cross-validation. The predicted results induce some accuracy measurements (*e.g.*, overall prediction accuracy, MCC).
3. Select one measurement of accuracy to be the key measurement. The feature set that yields the best key measurement is considered to be the optimal combination of features for classification.

## Results

### Findings of the mRMR method

The mRMR method was executed on a dataset that consisted of 1,267 fecal samples from individuals of three different races. This method yielded two lists, which were obtained as mentioned in Section “mRMR method”. Because of our limited computational power, we analyzed only the top 1,000 features for each list. The resulting MaxRel and mRMR feature lists are provided in Tables S1 and S2. In the MaxRel feature list, the MI value of each feature is also listed.

### Findings of the IFS method

**Figure 1:**
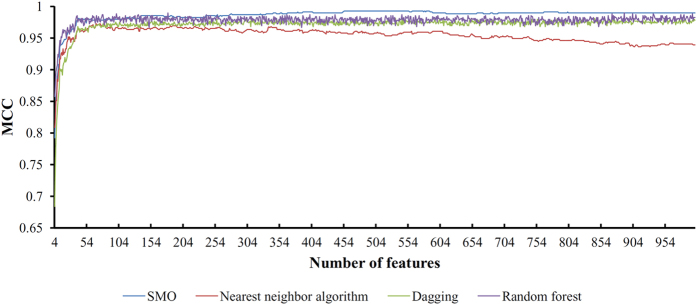
Four IFS curves show the results of four prediction engines obtained using the IFS method. The X-axis represents the number of features used for classification, whereas the Y-axis represents the MCC. It can be observed that the prediction engine SMO can produce the best performance.

Based on the mRMR feature list obtained in Section “Findings of the mRMR method”, the IFS method was executed on the dataset in which each of four prediction engines: random forest, nearest neighbor algorithm, SMO, and Dagging, was used as the basic machine-learning algorithm one by one. For each set *F*_*i*_ and a prediction engine, we calculated the MCC, overall prediction accuracy, and the accuracy for each of the three races. The above values for four prediction engines are all provided in Tables S3–S6, respectively. As mentioned in Section “Accuracy measurement”, the MCC was selected as the key measurement for the extraction of important features and to build an optimal prediction model for each prediction engine. For ease of observation, we plotted an IFS curve for each prediction engine by setting the MCC as the Y-axis and the number of features contributing to the classification as the X-axis. The resulting four curves are shown in Fig. 1, from which we can observe that the prediction engine SMO can produce the best performance. The measurements of the best performance for four prediction engines are listed in Table 1, from which the highest MCCs for random forest, nearest neighbor algorithm, SMO, and Dagging were 0.990, 0.972, 0.993 and 0.981, respectively. These MCCs were obtained by using the first 890, 80, 454, 956 features from the mRMR feature list. According to the fact that the SMO produced the best performance, we selected the optimal SMO prediction model as the optimal prediction model, in which the first 454 features from the mRMR feature list were used to represent samples, and the SMO was used as the prediction engine. In addition, these 454 features were deemed to be the optimal combination of features for classification. The IDs for the 454 extracted genes are presented in the mRMR feature list in Table S2.

## Discussion

**Figure 2:**
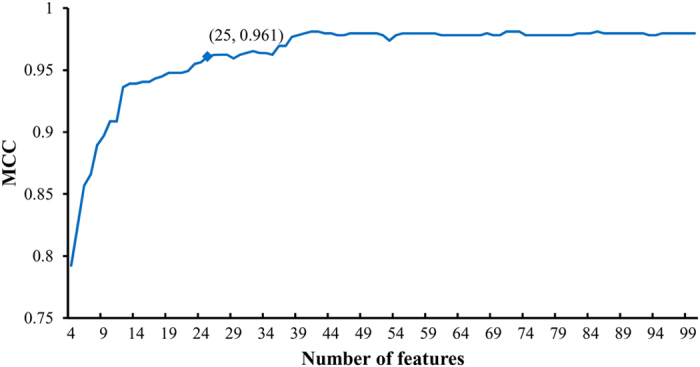
The IFS curve of the SMO between X-axis 4 and X-axis 100. The X-axis represents the number of features used for classification, whereas the Y-axis represents the MCC. It can be observed that the first 25 features in the mRMR feature list yielded a MCC greater than 0.960.

**Figure 3:**
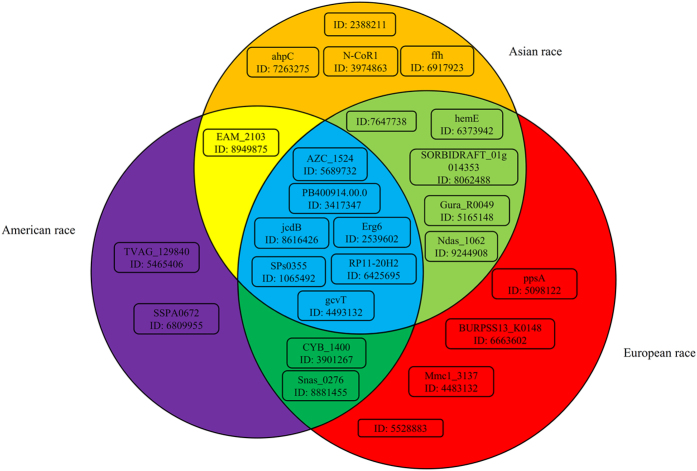
The relationships among 25 important genes and three different races. Three circles represent the sets of genes that are specific to American, Asian and European races, respectively. Genes in the overlap of circles indicate that they are specific to multiple races. A total of ten genes are specific to only one race, whereas eight genes are specific to two races, and seven genes are shared by all three races analyzed.

A set of 454 genes was used for the optimal prediction model. However, it is very difficult to analyze each gene one-by-one. As shown in Fig. 1, the IFS curve of SMO initially follows a sharp increasing trend, after which the MCC is maintained at a high level. Thus, we believe that those features with high ranks in the mRMR feature list are more important than other features. By amplifying the IFS-curve between X-axis 4 and 100 (see Fig. 2), the first 25 features in the mRMR feature list alone yielded a MCC of 0.961. Thus, we mainly focused on these 25 features (listed in Table 2) and discuss them below in this section. It can be observed from Table 2 that features with high MI values do not always receive high ranks in the mRMR feature list because there exist redundancies between them. Among these 25 genes, some of them are specific to a single race, and some are specific to two races, whereas others are common to all three races. Figure 3 shows the relationships among these 25 genes and the three different races that we analyzed.

### Genes specific to one single race

As we all know, the gut microbe system is completely modified and established after birth. Therefore, the gut microbe system of different ethnic groups may be affected by both the genetic background and the specific external environment^13^. According to our identification, ten genes were found in only one specific race, which are described below. **TVAG_129840** (ID: 5465406) is a unique gene of *Trichomonas vaginalis G3* that is specifically expressed in the microbiome of Americans^25^. As an anaerobic, flagellated protozoan parasite, Trichomonas vaginalis turns out to be the causative agent of trichomoniasis and may further induce vaginitis, which badly threaten the health of adult female^26^. Although *Trichomonas vaginalis G3* has not been reported to invade the intestinal tissues, the horizontal gene transfer between *Trichomonas vaginalis G3* and *Entamoeba histolytica*, a common intestinal pathogens has been reported, suggesting that such gene may be transferred to *Entamoeba histolytica* and further identified by the microbiome data analysis^27^. Therefore, the identification of *Trichomonas vaginalis G3* in stool samples may be induced by the coinfection of *Trichomonas vaginalis G3* and *Entamoeba histolytica*. What’s more, *Trichomonas vaginalis G3* has been shown to be more infectious for African race, which has a high frequency in American groups (comparing with another two ethnic groups), validating our identification of American specific microbiome. According to the results, another functional gene, **SSPA0672** (ID: 6809955) is also specifically expressed in the gut microbe of American ethnic groups. Such gene has been identified to contribute to nitrogen associated metabolic processes in human gut. As we all know, Americans, compared to the Asians and the Mediterraneans (a subgroup of Europeans) have quite fewer people with lactose intolerance and deficiency. Considering that our predicted gene contribute to nitrogen-associated processes which is quite significant for lactose metabolism, the discrepant distribution of such gene may partially contribute to the regional functional differences of lactose metabolism processes, which in turn, validates the accuracy of our algorithm^28^.

Four genes were predicted to be specific to European fecal samples. Phosphoenolpyruvate synthase, which is encoded by one of them, **ppsA** (ID: 5098122), has only been identified in *Pseudomonas stutzeri*. *Pseudomonas stutzeri* is a non-fluorescent denitrifying bacterium bacteria that has been regarded as a conditioned pathogen of humans^29^. Strikingly, *Pseudomonas stutzeri* has been reported to be associated with lactate metabolism and may contribute to the intake and digestion of lactate in intestinal tissues^30^. One in sixty thousand newborns in Finland, as the one of the typical European populations, perform as lactose intolerance^31^. However, along with the construction of intestinal flora after birth, according to adult data, Europeans showed the lowest rate of lactose intolerance (comparing with another two ethnic groups) which may be induced by the population-specific colonization of *Pseudomonas stutzeri*^32^. Another European-specific gene is **BURPSS13_K0148** (ID: 6663602), which contributes to amino acid transport and metabolism in *Burkholderia pseudomallei S13*. In Southeast Asia and Northern Australia, *Burkholderia pseudomallei* is the causative pathogen of a specific disease, melioidosis which can be spread through multiple routes including the digestive tract^33^. However, *Burkholderia pseudomallei* rarely cause severe diseases in Europe, no matter which ethnic groups, suggesting that the pathogenicity of such microbe may be regionally specific. Therefore, with low pathogenicity in Europe, such microbe may be widely carried and identified as symbiotic bacteria in European race. **Mmc1_3137** (ID: 4483132) is also in our prediction list. Such gene has been identified in *Roseburia* which belongs to *Phylum Firmicutes*. *Roseburia* has been confirmed to be a butyrate-producing bacterium in human intestine which participates in SCFA (short chain fatty acid) -associated energy metabolism processes^34^. It has been confirmed that the SCFA levels together with the related gut flora are different in various species, especially between Europeans and other species^35^. Therefore, such difference may be partially induced by the differential distributions of *Roseburia* with the specific gene we identified, validating our prediction of Mmc1_3137 as a European specific gut biomarker. Another gene, **MH0053_GL0075770** (ID: 5528883) has also been identified as a European-specific gut microbe, which is expressed in *Bacteroides*. The level of *Bacteroides* in fetal samples, which reflects the gut microbial environment, has been confirmed to be positively correlated with the assumption of protein and animal fat^36^. Considering the high-protein diet of Europeans and Americans comparing to Asians, it’s no wonder that such gene which is specifically expressed in *Bacteroides* has been identified as a European or American specific gene (as for Asians, only nomadic nations have similar diet). What’s more, gene MH0053_GL0075770 (ID: 5528883) contributes to type IV secretion processes of microbes which is associated with the immune response of the host. It has been reported that the Europeans and Americans react differently against the *Bacteroides*, suggesting that such immune associated, metabolic related gene may be expressed in the European gut microbe, validating our prediction^37^.

More specific genes have been uniquely detected in Asians. **AhpC** (ID: 7263275) (alkyl hydroperoxide reductase) is a unique gene in the Asian intestinal microbiome, which is mostly expressed in aphids and is associated with aphid glycol-metabolism^38^. Additionally, AhpC has been recently established to be expressed in *Helicobacterium* and may contribute to gastritis caused by *Helicobacter pylori*, which is more widespread in Asia^39^. Regulated by such gene, N-CoR1 (ID: 3974863) contributes to immunity and cell proliferation in intestinal tissues^40^. N-CoR1 has been shown to exhibit ethnic differences, especially between Asians and other races, which may be in accord with our hypotheses^41^. Another specific gene (ID: 2388211) is associated with intracellular trafficking, secretion and vesicular transport. Such gene is specifically expressed in *Caenorhabditis elegans* and have homologous protein in the human intestine that shares a similar function which is identified in Asian race^42^. The identification of such gene may be induced by mistaken mapping of homologous genes which still reflects the ethnic specificity of samples. The last gene **ffh** (ID: 6917923) encoding ffh signal recognition particle protein has also been identified in Asian populations. As we have mentioned above, *Bacteroides* has been generally regarded to be overexpressed in the gut microbe of people with a high-protein diet^36^. Considering the data sources of our analysis (partially from patients with the type 2 diabetes), the identification of such high-protein consumption associated gene may be interfere by the specific sequencing data from people with high-protein and fat consumption which is quite common in type 2 diabetes patients^14^. Apart from that, Asian nomadic nations as we have analyzed above may share similar diet with the Europeans, which may further explain the identification of such gene as an Asian specific gene signature based on our algorithm.

### Genes specific to two independent races

In addition to the race-specific genes, there were eight genes that we found to be expressed in two independent races. European and American races share two genes, **CYB_1400** (ID: 3901267) and **Snas_0276** (ID: 8881455). CYB_1400 is a specific gene that clustered with *Synechococcus*. The DNA component of *Synechococcus* has been reported to be able to pass through the food chain and may further accumulate in the consumers, especially in the Atlantic Ocean and its related water areas like the Mediterranean Sea^43^. The Atlantic Ocean and the Mediterranean Sea are the main source of marine products for Europeans and Americans. Therefore, such gene may accumulate via the food chain and finally turn out to be an identical gut microbe genetic marker of Europeans and Americans. Another gene, Snas_0276, encodes a unique thioesterase that is specifically expressed in *Stackebrandtia nassauensis*, which has been reported to be associated with the synthesis of phosphonates and sugar metabolism, especially in intestinal tissues^44^. What’s more, *Stackebrandtia nassauensis* has been shown to be located in hypersaline habitats. Since the Mediterranean Sea turns out to be a unique sea area with quite high salinity, *Stackebrandtia nassauensis* may be more likely to survive in such sea area and our predicted gene may also be accumulated via the food chain and finally enrich in the intestinal tissues of specific races.

**EAM_2103** (ID: 8949875) is shared by American and Asian individuals and is only expressed in *Erwinia amylovora*. *Erwinia amylovora* is not a pathogenic bacteria for humans, but can cause a disease known as fire blight in fruits from *Rosaceae*, such as apples and pears^45^. Just like microbes we have mentioned above, DNA of such pathogen can accumulate via the food chain and finally present in the human gut. Although such pathogen caused catastrophic decrease in agriculture production all over the world including Europe, it has not been reported in Europe until 1970s, suggesting that the accumulation of such pathogen’s DNA may not be extensive enough to be detected.

The remaining five genes are shared by Asian and European individuals. **HemE** (ID: 6373942) is a crucial uroporphyrinogen decarboxylase in *Chlorobium phaeobacteroides* has been identified in human intestine as a normal gut microbe^46^. There are two main subtypes of *Chlorobium phaeobacteroides* in natural habits (BS1 and DSM 266) which are respectively identified in east region of Black Sea, Western Asia and Lake Blankvann, Norway, reflecting the regional distribution of such microbe^47^. Therefore, since the microbe is mainly identified in Asia and Europe, the gene we identified may be reasonably accumulated in the gut of humans from such two regions. Another gene, **Gura_R0049** (ID: 5165148) is a transcription-related gene of *Geobacter uraniireducens*, which is well known because of its specific iron metabolic mechanism^48^. Such microbe has been identified mostly deep in the earth or in the heavy metal polluted area. The smelting and production processes of heavy metal mainly concentrated in some areas of Asia and Europe in respective historical period. However, North Americans contribute to the mining process with fewer heavy metal polluted areas, though there are still some restricted ones. Similarly, another gene (ID: 7647738) has been previously reported to be a gene that is expressed in tomato flower buds in certain locales and may be accumulated via the specific food chain. The intake methods of different ethnic group may lead to different gene abundances in the intestinal tract^49^. **Ndas_1062** (ID: 9244908) is also shared by Asians and Europeans. Such gene is expressed in *Nocardiopsis dassonvillei subsp. dassonvillei DSM 43111*. Although such microbe has been identified all over the world (most in animals and livestock), however, it has only been identified in the fetal sample (gut microbe) of European and Asian people not in Americans up to now, validating our prediction of Asian and European specific gut microbe^50^,^51^. The gene, **SORBIDRAFT_01g014353** (ID: 8062488) which has been identified to be expressed in *Salmonella enterica subsp. enterica serovar Paratyphi A str. AKU_12601*. Just like *Nocardiopsis dassonvillei*, as we have mentioned above, *Salmonella enterica* has been recognized as a crucial microbe and pathogen distributed all over the world. However, the specific subtype of such microbe, *AKU_1260* has mainly been identified in Yuxi, China and Cambridge, UK, implying that such subtype of *Salmonella enterica* may specifically distribute in Asian and Europe, validating the accuracy of our prediction algorithm^52^,^53^.

### Genes shared by all three races

All three races shared the other seven predictive genes. **AZC_1524** (ID: 5689732), which was present at the highest frequency, encodes a specific flavin-dependent oxidoreductase in *Azorhizobium caulinodans*. It has been established to be the symbiotic bacteria of *Sesbania* Scop, which is an important feed for poultry and domestic animals^54^. Therefore, this gene may be carried into the intestinal tract by our daily food. The second most frequent gene is a transcriptional factor of *Dictyostelium discoideum*, **jcdB** (ID: 8616426). Considering the wide distribution of *Dictyostelium discoideum*, especially in water, it is quite normal for the DNA of such microbe to accumulate via food chain and have such transcripts be present in bowels^55^. Another gene, **erg6** (ID: 2539602), is also associated with transcription regulation. As it is expressed in *Schizosaccharomyces pombe*, erg6 may be carried into the human gastrointestinal tract by the intake of fermented grains or molasses^56^. Another gene, **gcvT** (ID: 4493132), encodes a specific glycine-cleaving aminomethyltransferase T in *E. coli*, which may participate in the metabolism of glycine^57^. Such a gene may contribute to the colonization of *E. coli* in the human digestive tract, as well as to the intake of glycine, which is crucial for all races. **RP11-20H2** (ID: 6425695), which is a specific gene from a plant source is also in our prediction list. Originating from *Capsicum annuum*, a worldwide food, this gene may be carried into our intestine by foods that are present globally^58^. The co-expression of RP11-20H2 among the three races demonstrates the globalization of modern food supply. Gene **PB400914.00.0** (ID: 3417347) is also in our prediction list. Such gene is expressed in a specific *Plasmodium Berghei*, which has been detected all over the world (Asian, America and Europe)^59^. The last gene **SPs0355** (ID: 1065492) expressed by specific pathogen *Streptococcus pyogenes SSI-1* encodes the specific subunit of PTS system mannose-specific transporter. As a component of gut microbe, such bacteria has been identified all over the world and has been confirmed to have extensive diversity due to global-scale transmission processes^60^.

**Figure 4:**
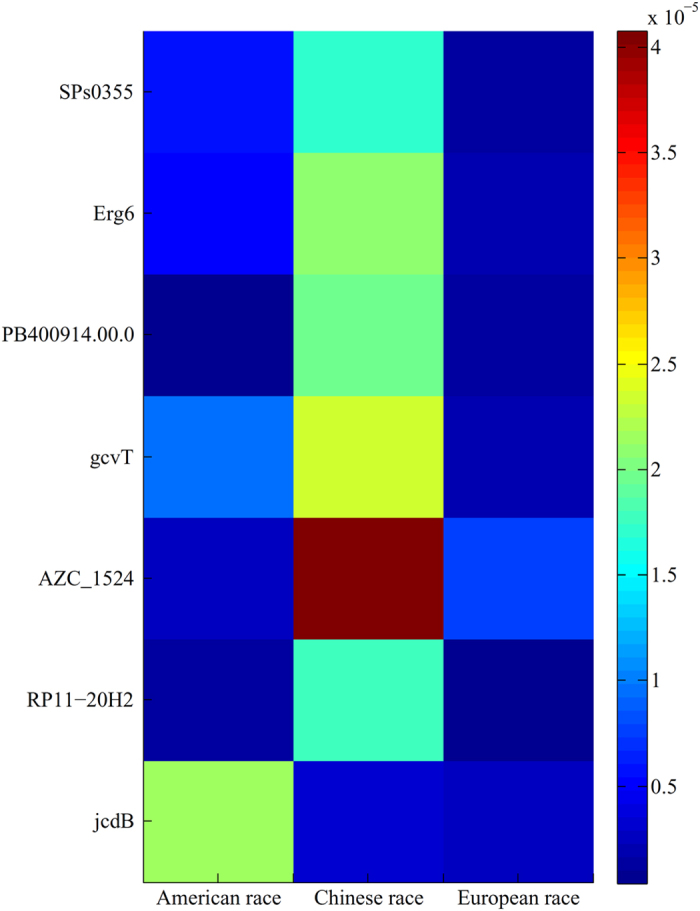
A heat map showing the expression levels of seven genes shared by three races on different races.

Although the above seven genes are shared by all three races, their expression levels to the gut microbiomes of three races are of great difference, thereby giving contributions for distinguishing the gut microbiomes of different races. To confirm this, we picked out the values of all samples under these features (genes) and calculated the mean values on different races. A heat map, as shown in Fig. 4, was plotted to illustrate these mean values, from which we can see that the expression levels of these seven genes on samples of different races are much different. For example, the expression level of gene AZC_1524 on Chinese race is about 15 times and 5 times of that on American and European races, respectively.

Overall, the diversity of gene profile we analyzed above reflects differences in eating habits, genetic susceptibility, living environments and metabolic levels among the races. Because such genes each have their respective functions, appreciating the variation in the human gut microbiome may help us learn more about the potential mechanisms that operate in the gut microenvironment and the interactions between those microorganisms and the human body. Comparing with the integrated gene catalog reported by Jun Wang & Peer Bork, this study further identify the gene profile diversity of different races, not just countries^14^. All in all, this study offers a new perspective to identify metabolic and structural gut microbiome differences between different races and contribute to further study of the intestinal flora.

## Conclusions

This study characterized the gut microbiomes of individuals from three different races. The extracted genes reflect differences among these races, such as eating habits, living environments and metabolic levels, which are also important factors that can influence the composition of the gut microbiome. We hope that the new findings presented in this study may yield new insights into studies of the gut microbiome.

## Additional Information

**How to cite this article**: Chen, L. *et al.* Gene expression profiling gut microbiota in different races of humans. *Sci. Rep.* **6**, 23075; doi: 10.1038/srep23075 (2016).

## Supplementary Material

Supplementary Table

## Figures and Tables

**Table 1:** The best performance of four prediction engines.

Prediction engine	Number of features	Accuracy for American race	Accuracy for Asian race	Accuracy for European race	Overall prediction accuracy	MCC
Random forest	890	0.986	0.989	0.999	0.994	0.990
Nearest neighbor algorithm	80	0.964	0.981	0.991	0.985	0.972
SMO	454	0.986	0.992	1.000	0.996	0.993
Dagging	956	0.964	0.978	1.000	0.990	0.981

**Table 2:** Genes that are the most important for distinguishing gut microbiome from different races.

Gene ID	Gene symbol	Gene name	Species Annotation (Phylum Level)	Species Annotation (Genus Level)	Gene Function	MI value	Rank in the mRMR feature list
5098122	ppsA	O2.UC22-1_GL0086706	Firmicutes	unknown	Carbon metabolism, methane metabolism,pyruvate metabolism	0.328	1
2388211	—	N032A_GL0031359	Bacteroidetes	Bacteroides	Intracellular trafficking, secretion, and vesicular transport	0.256	3
5465406	TVAG_129840	763982056-stool2_revised_scaffold11317_1_gene87646	Bacteroidetes	Bacteroides	Intracellular trafficking, secretion, and vesicular transport	0.253	7
3974863	N-CoR1	NLM004_GL0000416	Bacteroidetes	unknown	Transcription associated processes, immune associated processes	0.252	11
6917923	ffh	N013A_GL0070806	Bacteroidetes	Bacteroides	Bacterial secretion, protein export processes	0.238	20
8616426	jcdB	508703490-stool1_revised_scaffold18525_1_gene60248	Bacteroidetes	Bacteroides	Function unknown	0.238	15
5528883	–	MH0053_GL0075770	Bacteroidetes	Bacteroides	Intracellular trafficking, secretion, and vesicular transport	0.236	22
7263275	ahpC	T2D-120A_GL0064788	Bacteroidetes	Bacteroides	Transcriptiion associated processes	0.236	16
7647738	—	V1.UC49-0_GL0182914	Bacteroidetes	Bacteroides	Function unknown	0.234	5
6373942	hemE	V1.UC35-4_GL0001594	Firmicutes	unknown	Amino acid transport and metabolism, uroporphyrinogen metabolism	0.229	2
5165148	Gura_R0049	T2D-132A_GL0073376	Bacteroidetes	Bacteroides	Translation, ribosomal structure and biogenesis, aminoacyl-tRNA biosynthesis	0.224	9
6809955	SSPA0672	764143897-stool2_revised_C578533_1_gene62401	Bacteroidetes	Bacteroides	Transferring nitrogenous groups	0.216	19
8881455	Snas_0276	508703490-stool1_revised_scaffold1234_2_gene24275	Bacteroidetes	Bacteroides	Thioesterase associated metabolism processes	0.214	13
8062488	SORBIDRAFT_01g014353	V1.CD12-3_GL0107538	Bacteroidetes	Alistipes	Function unknown	0.195	24
5689732	AZC_1524	MH0135_GL0082377	Firmicutes	unknown	General function prediction only	0.185	6
6663602	BURPSS13_K0148	V1.FI03_GL0058372	Firmicutes	unknown	Amino acid transport and metabolism	0.184	14
3417347	PB400914.00.0	MH0346_GL0140692	unknown	unknown	Secondary metabolites biosynthesis, transport and catabolism (General function prediction only)	0.173	18
8949875	EAM_2103	DLF001_GL0011563	Firmicutes	Streptococcus	Function unknown	0.169	10
2539602	erg6	MH0444_GL0048102	Bacteroidetes	Bacteroides	Transcription, steroid biosynthesis, ergocalciferol biosynthesis	0.169	4
4483132	Mmc1_3137	MH0311_GL0031693	Firmicutes	Roseburia	Porphyrin and chlorophyll metabolism	0.162	21
3901267	CYB_1400	MH0150_GL0048296	Firmicutes	unknown	Function unkown	0.158	12
6425695	RP11-20H2	V1.CD54-0_GL0116394	Bacteroidetes	Alistipes	Function unknown	0.158	8
4493132	gcvT	V1.UC4-5_GL0212543	Bacteroidetes	Bacteroides	Signal transduction mechanisms, carbon metabolism, Glyoxylate and dicarboxylate metabolism	0.156	17
9244908	Ndas_1062	DLF004_GL0039505	unknown	unknown	Function unknown	0.146	23
1065492	SPs0355	MH0077_GL0010885	Bacteroidetes	Bacteroides	Amino sugar and nucleotide sugar metabolism, fructose and mannose metabolism, phosphotransferase system (PTS) associated biological processes	0.146	25
