# Supplementary material for: Gene expression profiling gut microbiota in different races of humans
Source: Sci Rep. 2016 Mar 15;6:23075. doi: 10.1038/srep23075 (PMC4791684; doi:10.1038/srep23075)
Supplement: Supplementary Table [file srep23075-s1.pdf]

Gene expression profiling gut microbiota in different races of humans

Lei Chen, Yu-Hang Zhang, Tao Huang, and Yu-Dong Cai

Table S1. MaxRel feature list obtained by mRMR method

Order	Feature name (gene ID)	MI value
1	5098122	0.328
2	2388211	0.256
3	5465406	0.253
4	3974863	0.252
5	8616426	0.238
6	6917923	0.238
7	5528883	0.236
8	7263275	0.236
9	7647738	0.234
10	6373942	0.229
11	5165148	0.224
12	6487507	0.221
13	6809955	0.216
14	8881455	0.214
15	5920015	0.214
16	7067500	0.201
17	8212155	0.2
18	9363763	0.196
19	820922	0.195
20	8062488	0.195
21	6483426	0.195
22	6236815	0.19
23	5839247	0.189
24	7325156	0.188
25	8374351	0.188
26	5689732	0.185
27	7969462	0.185
28	6663602	0.184
29	6198080	0.182
30	3631729	0.181
31	2791564	0.178
32	7152364	0.176
33	769245	0.175
34	7665161	0.173
35	967612	0.173
36	8793127	0.173
37	3417347	0.173

38	7654325	0.172
39	4670212	0.171
40	2539602	0.169
41	8949875	0.169
42	8380075	0.169
43	7196752	0.168
44	6967249	0.168
45	7808350	0.168
46	8864827	0.167
47	1498055	0.167
48	9457082	0.166
49	8238982	0.166
50	5819707	0.165
51	6950798	0.165
52	9303112	0.164
53	8411581	0.164
54	7685628	0.164
55	8173226	0.164
56	9162268	0.164
57	6569244	0.163
58	8381050	0.162
59	8761619	0.162
60	4483132	0.162
61	2003434	0.161
62	5914227	0.161
63	8389950	0.16
64	9651057	0.159
65	6925485	0.159
66	7325186	0.159
67	6425695	0.158
68	5291379	0.158
69	3901267	0.158
70	4716906	0.158
71	6552573	0.157
72	8535223	0.156
73	4493132	0.156
74	9165384	0.155
75	2347706	0.155
76	9074465	0.155
77	8140126	0.155
78	9034657	0.154
79	7494205	0.154

80	4064699	0.153
81	7469336	0.152
82	7368417	0.152
83	6623843	0.152
84	8574879	0.152
85	8546832	0.152
86	6175579	0.151
87	3137088	0.151
88	3870661	0.151
89	1788883	0.151
90	6923966	0.151
91	3387149	0.15
92	9716358	0.15
93	5436646	0.15
94	4517777	0.149
95	4818620	0.149
96	7562332	0.149
97	7964617	0.149
98	167092	0.149
99	7262922	0.149
100	1053790	0.148
101	8073962	0.148
102	7944344	0.148
103	1638573	0.148
104	4541155	0.148
105	5886479	0.148
106	7626865	0.148
107	6180723	0.148
108	6584971	0.148
109	7779840	0.148
110	1042188	0.147
111	1721263	0.147
112	7637701	0.147
113	6318660	0.146
114	8043052	0.146
115	7555191	0.146
116	7649432	0.146
117	934950	0.146
118	8574459	0.146
119	8336327	0.146
120	3952917	0.146
121	9244908	0.146

122	6782303	0.146
123	1065492	0.146
124	3946216	0.145
125	2783595	0.145
126	8460143	0.145
127	5964010	0.145
128	1307208	0.145
129	7362059	0.145
130	9649021	0.145
131	1057169	0.144
132	8268633	0.144
133	6249854	0.144
134	9496247	0.144
135	848350	0.144
136	5046426	0.144
137	6606805	0.144
138	7188868	0.144
139	7906635	0.144
140	3115639	0.143
141	2707872	0.143
142	9185473	0.143
143	6769051	0.143
144	4912641	0.143
145	2692662	0.143
146	9690270	0.143
147	3985401	0.143
148	1316998	0.142
149	7988121	0.142
150	2178931	0.142
151	7232634	0.142
152	6962348	0.142
153	4382685	0.141
154	6916260	0.141
155	4519541	0.141
156	7895354	0.141
157	9647862	0.141
158	8572373	0.141
159	5203152	0.141
160	6355465	0.141
161	6303800	0.141
162	7260507	0.14
163	3705709	0.14

164	3785085	0.14
165	1109807	0.14
166	8232071	0.14
167	6583135	0.14
168	5606398	0.14
169	8136472	0.14
170	8490775	0.14
171	2098249	0.14
172	1392099	0.14
173	7092762	0.14
174	7035028	0.14
175	8240540	0.139
176	4082506	0.139
177	7733567	0.139
178	1840806	0.139
179	3690622	0.139
180	8266589	0.139
181	6320274	0.139
182	8452753	0.139
183	4632508	0.139
184	7248142	0.139
185	7187921	0.138
186	5827184	0.138
187	7416400	0.138
188	303867	0.138
189	6882272	0.138
190	4842570	0.138
191	206700	0.138
192	4433848	0.138
193	8209782	0.137
194	2351443	0.137
195	8916358	0.137
196	7840733	0.137
197	7354160	0.137
198	694449	0.137
199	4110560	0.137
200	7000841	0.137
201	3455438	0.137
202	7712055	0.137
203	8844117	0.137
204	7883793	0.137
205	3884682	0.137

206	5102690	0.137
207	6433467	0.137
208	1929480	0.136
209	880759	0.136
210	2641562	0.136
211	4866741	0.136
212	1739382	0.136
213	6837770	0.136
214	3145217	0.136
215	1368454	0.136
216	7342104	0.136
217	3808418	0.136
218	5198754	0.136
219	7524300	0.135
220	2640614	0.135
221	4728208	0.135
222	7877735	0.135
223	7960901	0.135
224	4910837	0.135
225	8740546	0.135
226	7708348	0.135
227	8044750	0.135
228	7068724	0.135
229	7067404	0.134
230	268152	0.134
231	3570019	0.134
232	8772194	0.134
233	1812704	0.134
234	7875286	0.134
235	2527629	0.134
236	8217691	0.134
237	7611716	0.134
238	6476438	0.134
239	7521849	0.133
240	8554280	0.133
241	4206059	0.133
242	2031193	0.133
243	7255402	0.133
244	2793387	0.133
245	7202377	0.133
246	6536549	0.133
247	4234151	0.133

248	524218	0.133
249	556525	0.133
250	7351495	0.133
251	7774956	0.133
252	4812931	0.133
253	7775715	0.133
254	9372805	0.133
255	7172226	0.132
256	5479861	0.132
257	8153263	0.132
258	7497005	0.132
259	6484880	0.132
260	3607460	0.132
261	3131297	0.132
262	7785784	0.132
263	6334067	0.132
264	9871218	0.132
265	5082759	0.132
266	4517170	0.132
267	3623917	0.131
268	4950554	0.131
269	6949181	0.131
270	1383475	0.131
271	1719438	0.131
272	8037231	0.131
273	8832451	0.131
274	5217041	0.131
275	7475506	0.131
276	7747571	0.13
277	310530	0.13
278	9319600	0.13
279	576765	0.13
280	1193700	0.13
281	8723500	0.13
282	7184920	0.13
283	2231078	0.13
284	7168722	0.13
285	7090877	0.13
286	5042335	0.13
287	1554460	0.13
288	4164214	0.13
289	3363441	0.129

290	6703827	0.129
291	4465242	0.129
292	710143	0.129
293	4612940	0.129
294	8310337	0.129
295	6291759	0.129
296	303973	0.129
297	8585230	0.129
298	9313020	0.129
299	7525392	0.129
300	1649508	0.129
301	7741661	0.129
302	7470105	0.129
303	4677255	0.129
304	1242620	0.128
305	9432152	0.128
306	1660056	0.128
307	4621837	0.128
308	4857730	0.128
309	4859122	0.128
310	3244801	0.128
311	3746862	0.128
312	8378964	0.128
313	4472109	0.128
314	9661301	0.128
315	4865521	0.128
316	1826624	0.128
317	4318240	0.128
318	6101171	0.128
319	7028884	0.128
320	1835656	0.128
321	7204396	0.127
322	4828955	0.127
323	2508145	0.127
324	8496396	0.127
325	9032356	0.127
326	7356311	0.127
327	3743113	0.127
328	3511632	0.127
329	1041140	0.127
330	956908	0.127
331	5360278	0.127

332	8075896	0.127
333	1679319	0.127
334	6561477	0.127
335	6867329	0.127
336	6688638	0.127
337	3184391	0.127
338	7131354	0.126
339	7062883	0.126
340	7269311	0.126
341	7912842	0.126
342	2169612	0.126
343	8136726	0.126
344	2780388	0.126
345	616614	0.126
346	2614413	0.126
347	2811137	0.126
348	1397015	0.126
349	9133898	0.126
350	8025733	0.126
351	9116453	0.126
352	6548886	0.126
353	7566039	0.125
354	7685726	0.125
355	7636732	0.125
356	7820728	0.125
357	3148305	0.125
358	4872045	0.125
359	6065732	0.125
360	8245862	0.125
361	281891	0.125
362	9355642	0.125
363	4612772	0.125
364	9580498	0.125
365	8168361	0.125
366	7971308	0.125
367	2649257	0.125
368	5914370	0.125
369	8295394	0.125
370	8232327	0.125
371	9158899	0.125
372	9135339	0.125
373	9848709	0.125

374	1921270	0.125
375	7945248	0.125
376	9799037	0.125
377	9766572	0.125
378	3390732	0.125
379	1662030	0.125
380	7060763	0.125
381	8801880	0.124
382	8615847	0.124
383	8820687	0.124
384	7196592	0.124
385	4125240	0.124
386	4422484	0.124
387	6403257	0.124
388	3549612	0.124
389	4457907	0.124
390	5906415	0.124
391	5023319	0.124
392	7977976	0.124
393	7809021	0.123
394	4984566	0.123
395	7483094	0.123
396	353395	0.123
397	6533245	0.123
398	6941479	0.123
399	7291769	0.123
400	9242814	0.123
401	5072538	0.123
402	7526217	0.123
403	1264894	0.123
404	1136915	0.123
405	9612386	0.123
406	1918248	0.123
407	7747649	0.123
408	2871936	0.123
409	1805565	0.123
410	6525937	0.123
411	7011362	0.123
412	7522691	0.123
413	8003474	0.123
414	1649976	0.123
415	2032669	0.123

416	8393098	0.123
417	8348328	0.123
418	2183373	0.123
419	4832096	0.123
420	9569161	0.123
421	7515373	0.122
422	3001646	0.122
423	2349672	0.122
424	7710556	0.122
425	5957138	0.122
426	8105008	0.122
427	9437718	0.122
428	2688543	0.122
429	8234880	0.122
430	8172427	0.122
431	9399531	0.122
432	6884809	0.122
433	7350156	0.122
434	4920458	0.122
435	5311360	0.122
436	7748987	0.122
437	7832252	0.121
438	5241833	0.121
439	7921637	0.121
440	6837231	0.121
441	6741216	0.121
442	104132	0.121
443	7558362	0.121
444	4943243	0.121
445	7328878	0.121
446	5396612	0.121
447	8695153	0.121
448	8206968	0.121
449	9359029	0.121
450	3036013	0.121
451	7679993	0.121
452	7996940	0.121
453	9393100	0.121
454	6296147	0.121
455	7966964	0.121
456	730467	0.121
457	4590176	0.121

458	8522361	0.121
459	7654344	0.12
460	3051030	0.12
461	1660057	0.12
462	8250650	0.12
463	1029150	0.12
464	6405757	0.12
465	6417752	0.12
466	1370021	0.12
467	8360852	0.12
468	2452650	0.12
469	4812334	0.12
470	5104883	0.12
471	6803221	0.12
472	7593558	0.12
473	4323846	0.12
474	484126	0.12
475	8715146	0.12
476	7092467	0.12
477	5108888	0.12
478	5374445	0.12
479	6938114	0.12
480	8753895	0.12
481	8768186	0.12
482	7968479	0.12
483	7270923	0.12
484	4626434	0.119
485	651436	0.119
486	9228678	0.119
487	9712389	0.119
488	5430192	0.119
489	1555454	0.119
490	3856323	0.119
491	6946286	0.119
492	7479270	0.119
493	9765942	0.119
494	2649994	0.119
495	8284655	0.119
496	6615446	0.119
497	8002338	0.119
498	7712653	0.119
499	8043508	0.119

500	3995980	0.119
501	8809398	0.119
502	5461428	0.119
503	8460750	0.119
504	7372735	0.119
505	9180441	0.119
506	5176001	0.119
507	7743258	0.118
508	1689743	0.118
509	7681192	0.118
510	953365	0.118
511	1104894	0.118
512	2324789	0.118
513	6702736	0.118
514	3462644	0.118
515	5741950	0.118
516	8312644	0.118
517	9458891	0.118
518	9577288	0.118
519	4338288	0.118
520	6754720	0.118
521	8051372	0.118
522	6895724	0.118
523	7812388	0.118
524	4298023	0.118
525	7066058	0.118
526	7762764	0.118
527	8633890	0.118
528	4472116	0.118
529	9385558	0.118
530	1293722	0.118
531	1622042	0.118
532	9573813	0.118
533	3504950	0.118
534	5849662	0.118
535	4105368	0.118
536	4425723	0.118
537	7673200	0.118
538	4037665	0.118
539	1396933	0.118
540	7034847	0.118
541	3996465	0.118

542	1054709	0.118
543	281220	0.118
544	3064552	0.117
545	5079601	0.117
546	7753951	0.117
547	1323038	0.117
548	5539363	0.117
549	8037243	0.117
550	5938747	0.117
551	2285744	0.117
552	1117703	0.117
553	1156041	0.117
554	9692371	0.117
555	7473439	0.117
556	8387363	0.117
557	9768026	0.117
558	2637188	0.117
559	8075606	0.117
560	437849	0.117
561	7262486	0.117
562	3002489	0.117
563	5745460	0.117
564	2202385	0.117
565	4729990	0.116
566	9221487	0.116
567	8650543	0.116
568	8916629	0.116
569	8046944	0.116
570	3148367	0.116
571	3323510	0.116
572	8303610	0.116
573	4573728	0.116
574	2522342	0.116
575	7930718	0.116
576	9016264	0.116
577	8588327	0.116
578	8927540	0.116
579	9483731	0.116
580	9702525	0.116
581	5287980	0.116
582	9281152	0.116
583	9806737	0.116

584	1157171	0.116
585	9342284	0.116
586	8685615	0.116
587	9543076	0.116
588	9090647	0.116
589	6738433	0.116
590	4837283	0.116
591	6646862	0.116
592	8527713	0.116
593	2780453	0.115
594	398822	0.115
595	1279543	0.115
596	3766170	0.115
597	5616853	0.115
598	773254	0.115
599	8429177	0.115
600	8060075	0.115
601	6161276	0.115
602	5317017	0.115
603	7407685	0.115
604	7528479	0.115
605	8585286	0.115
606	4329531	0.115
607	1629710	0.115
608	2595039	0.115
609	7553434	0.115
610	8687817	0.115
611	10944	0.115
612	6079401	0.115
613	9314089	0.115
614	7438314	0.115
615	4845926	0.115
616	7534471	0.115
617	7320570	0.115
618	7602444	0.115
619	4858029	0.115
620	2873126	0.115
621	5055278	0.115
622	6190083	0.115
623	2306785	0.115
624	6021642	0.115
625	2659131	0.114

626	8065282	0.114
627	8065293	0.114
628	7826639	0.114
629	4626271	0.114
630	5492311	0.114
631	9690453	0.114
632	2688667	0.114
633	7415763	0.114
634	8874747	0.114
635	4641328	0.114
636	401775	0.114
637	6834452	0.114
638	8396899	0.114
639	8365374	0.114
640	4942009	0.114
641	7090800	0.114
642	9695261	0.114
643	8838223	0.114
644	5864688	0.114
645	1459592	0.114
646	1759107	0.114
647	9693262	0.114
648	4454749	0.114
649	1529580	0.114
650	8242714	0.114
651	1502298	0.114
652	8757352	0.114
653	3426515	0.114
654	1373653	0.114
655	8048934	0.114
656	5720304	0.114
657	7228108	0.114
658	9447457	0.114
659	4353052	0.114
660	8422959	0.114
661	961471	0.114
662	5846743	0.114
663	9033851	0.114
664	9359652	0.114
665	5164791	0.114
666	7797808	0.114
667	7144921	0.113

668	5995387	0.113
669	7587949	0.113
670	3368125	0.113
671	124955	0.113
672	8038046	0.113
673	8543033	0.113
674	1018468	0.113
675	3201453	0.113
676	7060590	0.113
677	645821	0.113
678	987735	0.113
679	5133685	0.113
680	4079488	0.113
681	3240366	0.113
682	8283108	0.113
683	7299767	0.113
684	6734009	0.113
685	1999692	0.113
686	2327465	0.113
687	9556151	0.113
688	3504949	0.113
689	7606203	0.113
690	6485169	0.113
691	8384185	0.113
692	6945585	0.113
693	2071466	0.113
694	7356857	0.113
695	5401466	0.113
696	8706506	0.113
697	1035437	0.113
698	1990367	0.113
699	8425949	0.112
700	7378680	0.112
701	8036213	0.112
702	4058216	0.112
703	3407463	0.112
704	2176579	0.112
705	5222051	0.112
706	8060702	0.112
707	7004856	0.112
708	9638887	0.112
709	8449152	0.112

710	7378113	0.112
711	6225942	0.112
712	9614629	0.112
713	462693	0.112
714	7840702	0.112
715	7803204	0.112
716	4403723	0.112
717	7606609	0.112
718	7498637	0.112
719	6808976	0.112
720	9767650	0.112
721	9262225	0.112
722	3628339	0.112
723	6945928	0.112
724	4038289	0.112
725	9209579	0.112
726	9331534	0.112
727	8695275	0.112
728	6013567	0.112
729	6535163	0.112
730	4015428	0.112
731	155900	0.112
732	7570729	0.112
733	3197165	0.112
734	585198	0.112
735	58042	0.112
736	8255214	0.111
737	4175584	0.111
738	6662916	0.111
739	1356893	0.111
740	7996070	0.111
741	4604899	0.111
742	2387578	0.111
743	2823300	0.111
744	6376718	0.111
745	8565151	0.111
746	4561757	0.111
747	7375452	0.111
748	4662226	0.111
749	6160264	0.111
750	2834098	0.111
751	3292524	0.111

752	3316866	0.111
753	7542500	0.111
754	5686495	0.111
755	1130254	0.111
756	6066044	0.111
757	7320989	0.111
758	7780447	0.111
759	5931209	0.111
760	3655894	0.111
761	9318479	0.111
762	8574275	0.111
763	6199878	0.111
764	5847432	0.111
765	384573	0.111
766	9420117	0.111
767	8820988	0.111
768	8096757	0.111
769	4570887	0.111
770	9223590	0.111
771	5194412	0.111
772	6619671	0.111
773	7571249	0.111
774	9134239	0.111
775	7527797	0.111
776	7845623	0.111
777	9222052	0.111
778	6407417	0.111
779	7775704	0.111
780	6871901	0.111
781	7907111	0.11
782	2621532	0.11
783	630654	0.11
784	7233144	0.11
785	8211735	0.11
786	6118870	0.11
787	5724331	0.11
788	7906950	0.11
789	7502477	0.11
790	6725548	0.11
791	6060657	0.11
792	7780481	0.11
793	5400399	0.11

794	5681821	0.11
795	2733536	0.11
796	4355576	0.11
797	70970	0.11
798	9722595	0.11
799	7978266	0.11
800	7379146	0.11
801	7815749	0.11
802	9403440	0.11
803	1038068	0.11
804	5875841	0.11
805	812194	0.11
806	5819599	0.11
807	5739480	0.11
808	5317096	0.11
809	7749786	0.109
810	8991719	0.109
811	8838407	0.109
812	9287257	0.109
813	9043259	0.109
814	4442677	0.109
815	3879327	0.109
816	4748574	0.109
817	3181815	0.109
818	9606237	0.109
819	8418744	0.109
820	7330489	0.109
821	6430933	0.109
822	3179911	0.109
823	8278203	0.109
824	9393290	0.109
825	3098085	0.109
826	5728580	0.109
827	8447846	0.109
828	3954744	0.109
829	8247641	0.109
830	5376824	0.109
831	7166232	0.109
832	9064768	0.109
833	3188320	0.109
834	7423537	0.109
835	6967978	0.109

836	2352242	0.109
837	4227806	0.109
838	5397254	0.109
839	7519925	0.109
840	4941951	0.109
841	9210137	0.109
842	7323865	0.109
843	1170542	0.109
844	5280726	0.109
845	5800658	0.109
846	7772204	0.109
847	3171122	0.109
848	6621056	0.109
849	4235852	0.109
850	148096	0.109
851	2578374	0.109
852	6406621	0.109
853	7839719	0.108
854	9246728	0.108
855	2874149	0.108
856	5280450	0.108
857	254535	0.108
858	8252767	0.108
859	1659340	0.108
860	4832168	0.108
861	7704082	0.108
862	8611551	0.108
863	7995313	0.108
864	4637561	0.108
865	1680690	0.108
866	4403301	0.108
867	7152344	0.108
868	2964836	0.108
869	6433058	0.108
870	347424	0.108
871	682816	0.108
872	6080440	0.108
873	8495831	0.108
874	6470284	0.108
875	1811678	0.108
876	7539828	0.108
877	8191875	0.108

878	8233560	0.108
879	1570014	0.108
880	8241545	0.108
881	1235314	0.108
882	7433509	0.108
883	8988339	0.108
884	2414099	0.108
885	4944437	0.108
886	2235102	0.108
887	1220835	0.108
888	9429763	0.108
889	5963535	0.108
890	9104750	0.108
891	6788034	0.108
892	1417875	0.108
893	3778311	0.108
894	9116836	0.108
895	3242924	0.108
896	7977267	0.108
897	9313006	0.108
898	8629787	0.107
899	8141224	0.107
900	7744174	0.107
901	3571921	0.107
902	8810662	0.107
903	8996780	0.107
904	7996060	0.107
905	4007740	0.107
906	2958986	0.107
907	8092062	0.107
908	6645618	0.107
909	6062350	0.107
910	2626122	0.107
911	6460778	0.107
912	470486	0.107
913	8206927	0.107
914	9329883	0.107
915	4050852	0.107
916	7743523	0.107
917	1899535	0.107
918	7260237	0.107
919	8833955	0.107

920	8312192	0.107
921	7674401	0.107
922	6179868	0.107
923	5878724	0.107
924	4381797	0.107
925	3613669	0.107
926	5684770	0.107
927	2656097	0.107
928	5913343	0.107
929	8512177	0.107
930	7272412	0.107
931	6351691	0.107
932	7161250	0.107
933	2537188	0.107
934	1434438	0.107
935	859951	0.107
936	1064163	0.107
937	2145540	0.107
938	4843741	0.107
939	4703809	0.107
940	7720241	0.106
941	1300003	0.106
942	4244469	0.106
943	7200788	0.106
944	8343778	0.106
945	8211801	0.106
946	3408187	0.106
947	5888064	0.106
948	9652011	0.106
949	9398878	0.106
950	435097	0.106
951	9003958	0.106
952	3208241	0.106
953	6831858	0.106
954	6748046	0.106
955	203486	0.106
956	170297	0.106
957	7975453	0.106
958	3169978	0.106
959	5867503	0.106
960	1006907	0.106
961	7077804	0.106

962	5829560	0.106
963	7935482	0.106
964	4638865	0.106
965	4477994	0.106
966	1394759	0.106
967	5256483	0.106
968	7155788	0.106
969	6644011	0.106
970	621717	0.106
971	955831	0.106
972	7835734	0.106
973	1886666	0.106
974	7873021	0.106
975	8344342	0.106
976	9100095	0.106
977	3925728	0.106
978	5476346	0.106
979	6407277	0.106
980	8957278	0.106
981	8241379	0.106
982	1926601	0.105
983	2060178	0.105
984	7685985	0.105
985	802551	0.105
986	2234243	0.105
987	582492	0.105
988	7232263	0.105
989	1015393	0.105
990	8763743	0.105
991	5321821	0.105
992	6433345	0.105
993	8740155	0.105
994	2082371	0.105
995	6645909	0.105
996	3703308	0.105
997	165851	0.105
998	4615175	0.105
999	5772434	0.105
1000	3046010	0.105

Table S2. mRMR feature list obtained by mRMR method

Order	Feature name (gene ID)
1	5098122
2	6373942
3	2388211
4	2539602
5	7647738
6	5689732
7	5465406
8	6425695
9	5165148
10	8949875
11	3974863
12	3901267
13	8881455
14	6663602
15	8616426
16	7263275
17	4493132
18	3417347
19	6809955
20	6917923
21	4483132
22	5528883
23	9244908
24	8062488
25	1065492
26	6236815
27	5914227
28	6487507
29	3137088
30	7665161
31	769245
32	9363763
33	5920015
34	9716358
35	7325156
36	1042188
37	8212155
38	1057169
39	820922
40	8268633

41	967612
42	6569244
43	4433848
44	8374351
45	6175579
46	7067500
47	7626865
48	1739382
49	3631729
50	2791564
51	6198080
52	1109807
53	9651057
54	6483426
55	3115639
56	7152364
57	7494205
58	5839247
59	6923966
60	9303112
61	7969462
62	167092
63	9185473
64	8864827
65	3690622
66	7196752
67	1368454
68	3145217
69	6950798
70	1392099
71	8140126
72	6925485
73	2003434
74	1498055
75	1193700
76	7654325
77	6320274
78	8793127
79	6967249
80	8460143
81	7875286
82	4670212

83	4866741
84	9457082
85	8772194
86	1316998
87	9165384
88	7368417
89	8238982
90	3985401
91	7808350
92	9690270
93	6334067
94	694449
95	1053790
96	7775715
97	8761619
98	2098249
99	576765
100	9162268
101	8723500
102	4064699
103	6303800
104	5436646
105	9647862
106	1840806
107	8043052
108	206700
109	2707872
110	8389950
111	5291379
112	5819707
113	6916260
114	9871218
115	8380075
116	4865521
117	4859122
118	7469336
119	9074465
120	7351495
121	8336327
122	6552573
123	8173226
124	1679319

125	4716906
126	4910837
127	1307208
128	2347706
129	4519541
130	6180723
131	8240540
132	6584971
133	268152
134	8381050
135	1719438
136	7964617
137	4842570
138	7325186
139	4472109
140	1660056
141	8411581
142	9355642
143	524218
144	934950
145	6962348
146	8232071
147	1638573
148	3549612
149	6623843
150	3870661
151	7416400
152	7637701
153	2452650
154	6533245
155	7685628
156	7470105
157	4082506
158	9432152
159	6867329
160	4541155
161	556525
162	3387149
163	7733567
164	9372805
165	8574879
166	6561477

167	5046426
168	880759
169	1788883
170	9580498
171	7362059
172	310530
173	5102690
174	5886479
175	848350
176	710143
177	5827184
178	9034657
179	7944344
180	2811137
181	8535223
182	8073962
183	4818620
184	4612772
185	8245862
186	6249854
187	353395
188	2032669
189	4517777
190	6703827
191	5198754
192	9393100
193	7555191
194	7342104
195	2178931
196	3946216
197	4164214
198	303867
199	3705709
200	1397015
201	9496247
202	7988121
203	9116453
204	7262922
205	8136726
206	4517170
207	6782303
208	2614413

209	8217691
210	1383475
211	7354160
212	2692662
213	9649021
214	281891
215	8490775
216	3363441
217	4912641
218	8554280
219	4612940
220	7611716
221	8266589
222	5914370
223	1370021
224	6318660
225	956908
226	7883793
227	4382685
228	1264894
229	9766572
230	7840733
231	7475506
232	8003474
233	7187921
234	1835656
235	3785085
236	7062883
237	8452753
238	3244801
239	7685726
240	7035028
241	1721263
242	4465242
243	3570019
244	8572373
245	1555454
246	7255402
247	7562332
248	1826624
249	7232634
250	3607460

251	7248142
252	2351443
253	5906415
254	6355465
255	9799037
256	4125240
257	8546832
258	6484880
259	3746862
260	8136472
261	5360278
262	2527629
263	5964010
264	616614
265	8153263
266	7260507
267	2780388
268	9359029
269	5203152
270	7779840
271	1812704
272	7558362
273	7649432
274	6615446
275	2169612
276	8916358
277	5241833
278	4632508
279	9612386
280	6583135
281	2231078
282	1805565
283	7654344
284	2508145
285	7092762
286	4590176
287	4812334
288	8310337
289	4920458
290	8574459
291	3390732
292	6837770

293	9385558
294	3131297
295	5108888
296	104132
297	7712055
298	5311360
299	1242620
300	5082759
301	5023319
302	8844117
303	4728208
304	8585230
305	9690453
306	5072538
307	4110560
308	8172427
309	7708348
310	1660057
311	8393098
312	5606398
313	1396933
314	4422484
315	7521849
316	6291759
317	8250650
318	6769051
319	1041140
320	2641562
321	3455438
322	1029150
323	4943243
324	3952917
325	7968479
326	1649508
327	6882272
328	7090877
329	6738433
330	6433467
331	3051030
332	3036013
333	7188868
334	8687817

335	7000841
336	7996940
337	6606805
338	8206968
339	9180441
340	2649257
341	7906635
342	9692371
343	9090647
344	7971308
345	5042335
346	8037231
347	2031193
348	5079601
349	6949181
350	9135339
351	7168722
352	8768186
353	4457907
354	7068724
355	1759107
356	5479861
357	1662030
358	7524300
359	7350156
360	7515373
361	3808418
362	462693
363	9695261
364	6548886
365	8832451
366	3001646
367	5938747
368	6536549
369	9765942
370	2783595
371	6702736
372	303973
373	4828955
374	6688638
375	2688543
376	7877735

377	1293722
378	953365
379	4234151
380	8312644
381	8422959
382	1929480
383	8065282
384	9806737
385	3148305
386	7895354
387	8168361
388	9702525
389	1622042
390	9437718
391	3184391
392	8002338
393	4950554
394	1323038
395	7960901
396	5461428
397	9133898
398	7774956
399	630654
400	4323846
401	7269311
402	7060590
403	1054709
404	7636732
405	7679993
406	8522361
407	4984566
408	7785784
409	4338288
410	9661301
411	1649976
412	8740546
413	3996465
414	4079488
415	7172226
416	7262486
417	4626434
418	4872045

419	9319600
420	9577288
421	8695153
422	7407685
423	8044750
424	6525937
425	7028884
426	7741661
427	3462644
428	7184920
429	7743258
430	4677255
431	4206059
432	1117703
433	4832096
434	7525392
435	437849
436	1689743
437	8105008
438	9483731
439	3743113
440	9768026
441	9313020
442	8916629
443	4621837
444	484126
445	9032356
446	3323510
447	2324789
448	4837283
449	7912842
450	7681192
451	8303610
452	7144921
453	7747571
454	5176001
455	9543076
456	8209782
457	4105368
458	961471
459	8278203
460	6079401

461	4318240
462	6803221
463	6476438
464	8588327
465	7060763
466	3002489
467	3292524
468	7090800
469	2640614
470	8384185
471	3884682
472	5055278
473	8295394
474	6101171
475	7553434
476	6646862
477	8232327
478	8633890
479	1018468
480	7497005
481	1104894
482	5217041
483	8809398
484	9848709
485	2183373
486	3504950
487	3995980
488	651436
489	5374445
490	6485169
491	7483094
492	8075896
493	730467
494	2780453
495	7522691
496	7502477
497	9242814
498	5849662
499	2793387
500	2649994
501	4845926
502	9359652

503	8378964
504	3407463
505	1156041
506	1918248
507	1157171
508	5864688
509	7202377
510	6834452
511	6190083
512	7606203
513	5401466
514	7566039
515	3148367
516	7092467
517	6430933
518	9722595
519	2352242
520	6403257
521	6725548
522	2637188
523	2071466
524	7067404
525	2349672
526	2659131
527	1921270
528	5396612
529	987735
530	7809021
531	5616853
532	5164791
533	8075606
534	7204396
535	7004856
536	3098085
537	7977976
538	8038046
539	5133685
540	3623917
541	9767650
542	8801880
543	1459592
544	7152344

545	7753951
546	3511632
547	398822
548	8025733
549	5745460
550	7131354
551	7775704
552	6938114
553	2285744
554	1373653
555	281220
556	2522342
557	3179911
558	10944
559	7840702
560	155900
561	7906950
562	7748987
563	802551
564	1554460
565	7378680
566	7196592
567	4812931
568	5539363
569	9209579
570	9399531
571	2202385
572	4425723
573	3064552
574	8810662
575	7356311
576	6945585
577	7930718
578	9393290
579	5741950
580	4038289
581	9693262
582	165851
583	8820687
584	8615847
585	3504949
586	1502298

587	645821
588	7270923
589	7820728
590	4298023
591	6945928
592	2993014
593	124955
594	9573813
595	621717
596	1990367
597	254535
598	8496396
599	8585286
600	9614629
601	8360852
602	5430192
603	9262225
604	1394759
605	9712389
606	2235102
607	3368125
608	9654831
609	7832252
610	3426515
611	7710556
612	6748046
613	6644011
614	6065732
615	8348328
616	4058216
617	4227806
618	7571249
619	4615175
620	6417752
621	9134239
622	4472116
623	1035437
624	384573
625	6871901
626	8753895
627	4857730
628	7378113

629	4944437
630	2733536
631	6741216
632	6754720
633	3201453
634	1279543
635	6433058
636	5222051
637	7921637
638	9287257
639	7291769
640	7685985
641	9016264
642	6884809
643	58042
644	3703308
645	7826639
646	7593558
647	6066044
648	6460778
649	4641328
650	3169978
651	9569161
652	5681821
653	8234880
654	2306785
655	8685615
656	8512177
657	9318479
658	9100095
659	2595039
660	3181815
661	6946286
662	401775
663	2387578
664	6831858
665	5400399
666	9158899
667	773254
668	2414099
669	7966964
670	5963535

671	2234243
672	2873126
673	8283108
674	4244469
675	7839719
676	7328878
677	5957138
678	4662226
679	3197165
680	7034847
681	7161250
682	6405757
683	5287980
684	7570729
685	2688667
686	8046944
687	6662916
688	5728580
689	9281152
690	7835734
691	5104883
692	585198
693	812194
694	3856323
695	1013380
696	7299767
697	8734543
698	7473439
699	4604899
700	4329531
701	2327465
702	4175584
703	5280450
704	8574275
705	8650543
706	1006907
707	2871936
708	1170542
709	8284655
710	1659340
711	7812388
712	6645909

713	7415763
714	4450224
715	7747649
716	435097
717	3240366
718	7324109
719	7233144
720	4570887
721	9342284
722	9081913
723	6837231
724	8424410
725	8365374
726	7704082
727	1136915
728	2176579
729	8065293
730	2656097
731	7558041
732	8460750
733	9764098
734	5317017
735	5875841
736	2823300
737	6941479
738	1629710
739	7479270
740	8092062
741	5280726
742	8820988
743	9064768
744	1007888
745	7797808
746	7320989
747	7526217
748	5819599
749	3188320
750	5492311
751	8048934
752	2874149
753	5888064
754	9652011

755	8715146
756	203486
757	3628339
758	7762764
759	6160264
760	9222052
761	4381797
762	1235314
763	8387363
764	1199261
765	5127279
766	2578374
767	6296147
768	4970089
769	7539828
770	7066058
771	5724331
772	8580606
773	7945248
774	9003958
775	5755018
776	7744174
777	7320570
778	4139558
779	8043508
780	3655894
781	9329883
782	5194412
783	9458891
784	7780481
785	2338958
786	3766170
787	8740155
788	7606609
789	2253815
790	1570014
791	2621532
792	8242714
793	6062350
794	1529580
795	7749786
796	2874795

797	5995387
798	8425949
799	7712653
800	1038068
801	5720304
802	5829560
803	3925728
804	4858029
805	569482
806	8460901
807	7011362
808	1376843
809	3316866
810	7498637
811	1417875
812	2743275
813	9690171
814	4454749
815	955831
816	127397
817	1122213
818	7372735
819	1861639
820	8647092
821	9331534
822	8802035
823	7501699
824	9319206
825	3408777
826	2082371
827	3739551
828	4235852
829	945079
830	9228678
831	1356893
832	8706506
833	9321100
834	470486
835	8757352
836	5767767
837	682816
838	8801687

839	9606237
840	5739480
841	2834098
842	9032091
843	7743523
844	1826098
845	138292
846	7383923
847	3778311
848	8037243
849	5352828
850	648734
851	4573728
852	3616795
853	7079125
854	5317096
855	3084915
856	9110326
857	8233560
858	3862110
859	7423537
860	8874747
861	6021642
862	2841885
863	5714020
864	4613668
865	9514449
866	6326145
867	9314089
868	3571921
869	930612
870	4355576
871	8838223
872	9429763
873	9698219
874	4549242
875	6124201
876	5686495
877	1220835
878	5503668
879	6225942
880	899794

881	9638887
882	4941951
883	8954811
884	8927540
885	8543381
886	7644021
887	5931388
888	8429177
889	9403440
890	9320602
891	4442677
892	6139751
893	6535163
894	7260237
895	9420117
896	8211801
897	4626271
898	5193699
899	843583
900	8278231
901	9223590
902	3547883
903	8241545
904	9043259
905	9661204
906	4836962
907	7534471
908	7370386
909	4166531
910	6976497
911	7356857
912	8690526
913	2983671
914	8988339
915	9766994
916	6269574
917	4729990
918	6808976
919	4207243
920	89435
921	8449152
922	70970

923	6734009
924	5784618
925	8051372
926	3208241
927	7602444
928	3408187
929	1130254
930	791574
931	7996060
932	6572496
933	9556151
934	1811678
935	7228108
936	6407417
937	6062891
938	8382911
939	9210137
940	9107631
941	1279542
942	6895724
943	3477960
944	8543033
945	2211490
946	8799830
947	8919475
948	7287378
949	7673200
950	859951
951	6060657
952	8764663
953	8247641
954	1300699
955	3817366
956	4037665
957	649045
958	9104750
959	923103
960	3171122
961	4949073
962	1680690
963	4314237
964	7845623

965	8535919
966	4613652
967	8396899
968	7907111
969	1160683
970	8695275
971	5899447
972	5988494
973	2645278
974	5581051
975	8096757
976	579946
977	7900077
978	6351691
979	9033851
980	7438314
981	6139754
982	7621070
983	889276
984	7542500
985	753288
986	3918643
987	6271075
988	7873021
989	3459678
990	6013567
991	1242514
992	4832168
993	7392914
994	3724270
995	2452630
996	8060075
997	148096
998	4315237
999	9542672
1000	8772612

Table S3. The accuracies for different races and total prediction accuracy obtained by IFS method and SMO

Number of features	Accuracy for American race	Accuracy for Asian race	Accuracy for European race	Overall prediction accuracy	MCC
4	0.633094	0.798913	0.978947	0.888713	0.792331
5	0.805755	0.796196	0.976316	0.905288	0.824102
6	0.81295	0.839674	0.982895	0.922652	0.85688
7	0.856115	0.842391	0.981579	0.927388	0.865833
8	0.856115	0.875	0.986842	0.940016	0.889358
9	0.877698	0.875	0.989474	0.943962	0.896834
10	0.877698	0.888587	0.993421	0.950276	0.908682
11	0.877698	0.891304	0.992105	0.950276	0.908615
12	0.877698	0.934783	0.996053	0.965272	0.936195
13	0.892086	0.934783	0.996053	0.966851	0.939107
14	0.892086	0.934783	0.996053	0.966851	0.939107
15	0.899281	0.934783	0.996053	0.96764	0.940563
16	0.899281	0.934783	0.996053	0.96764	0.940563
17	0.899281	0.942935	0.994737	0.969219	0.943357
18	0.899281	0.942935	0.996053	0.970008	0.944875
19	0.913669	0.942935	0.996053	0.971586	0.947787
20	0.913669	0.942935	0.996053	0.971586	0.947787
21	0.906475	0.945652	0.996053	0.971586	0.94777
22	0.906475	0.94837	0.996053	0.972376	0.94921
23	0.906475	0.959239	0.996053	0.975533	0.95498
24	0.913669	0.959239	0.996053	0.976322	0.956434
25	0.913669	0.964674	0.997368	0.97869	0.960822
26	0.920863	0.964674	0.997368	0.979479	0.962274
27	0.920863	0.961957	0.998684	0.979479	0.962334
28	0.920863	0.961957	0.998684	0.979479	0.962334
29	0.920863	0.956522	0.998684	0.977901	0.959456
30	0.935252	0.956522	0.998684	0.979479	0.96236
31	0.935252	0.959239	0.998684	0.980268	0.963798
32	0.935252	0.959239	1	0.981058	0.965289
33	0.935252	0.959239	0.998684	0.980268	0.963798
34	0.942446	0.959239	0.997368	0.980268	0.963751
35	0.928058	0.959239	0.998684	0.979479	0.962334
36	0.928058	0.972826	0.998684	0.983425	0.969546
37	0.935252	0.972826	0.997368	0.983425	0.969513
38	0.935252	0.980978	1	0.987372	0.976819
39	0.94964	0.980978	0.998684	0.988161	0.978238
40	0.94964	0.983696	0.998684	0.98895	0.979685

41	0.94964	0.983696	1	0.98974	0.98116
42	0.94964	0.983696	1	0.98974	0.98116
43	0.94964	0.983696	0.998684	0.98895	0.979682
44	0.94964	0.983696	0.998684	0.98895	0.979682
45	0.94964	0.983696	0.997368	0.988161	0.978209
46	0.94964	0.983696	0.997368	0.988161	0.978209
47	0.94964	0.983696	0.998684	0.98895	0.979682
48	0.94964	0.983696	0.998684	0.98895	0.979682
49	0.94964	0.983696	0.998684	0.98895	0.979682
50	0.94964	0.983696	0.998684	0.98895	0.979682
51	0.94964	0.983696	0.998684	0.98895	0.979682
52	0.94964	0.986413	0.996053	0.988161	0.978193
53	0.94964	0.978261	0.996053	0.985793	0.973834
54	0.94964	0.986413	0.996053	0.988161	0.978193
55	0.94964	0.98913	0.996053	0.98895	0.979649
56	0.94964	0.98913	0.996053	0.98895	0.979649
57	0.956835	0.986413	0.996053	0.98895	0.979647
58	0.956835	0.986413	0.996053	0.98895	0.979647
59	0.956835	0.986413	0.996053	0.98895	0.979647
60	0.956835	0.986413	0.996053	0.98895	0.979647
61	0.94964	0.986413	0.996053	0.988161	0.978193
62	0.94964	0.986413	0.996053	0.988161	0.978193
63	0.94964	0.986413	0.996053	0.988161	0.978193
64	0.94964	0.986413	0.996053	0.988161	0.978193
65	0.94964	0.986413	0.996053	0.988161	0.978193
66	0.94964	0.986413	0.996053	0.988161	0.978193
67	0.94964	0.986413	0.996053	0.988161	0.978193
68	0.94964	0.986413	0.997368	0.98895	0.979661
69	0.94964	0.986413	0.996053	0.988161	0.978193
70	0.94964	0.986413	0.996053	0.988161	0.978193
71	0.964029	0.986413	0.996053	0.98974	0.981103
72	0.964029	0.986413	0.996053	0.98974	0.981103
73	0.964029	0.986413	0.996053	0.98974	0.981103
74	0.956835	0.983696	0.996053	0.988161	0.978195
75	0.956835	0.983696	0.996053	0.988161	0.978195
76	0.956835	0.983696	0.996053	0.988161	0.978195
77	0.956835	0.983696	0.996053	0.988161	0.978195
78	0.956835	0.983696	0.996053	0.988161	0.978195
79	0.956835	0.983696	0.996053	0.988161	0.978195
80	0.956835	0.983696	0.996053	0.988161	0.978195
81	0.956835	0.983696	0.996053	0.988161	0.978195
82	0.956835	0.986413	0.996053	0.98895	0.979648
83	0.956835	0.986413	0.996053	0.98895	0.979648

84	0.94964	0.986413	0.997368	0.98895	0.979661
85	0.94964	0.98913	0.997368	0.98974	0.981113
86	0.94964	0.986413	0.997368	0.98895	0.979661
87	0.94964	0.986413	0.997368	0.98895	0.979661
88	0.94964	0.986413	0.997368	0.98895	0.979661
89	0.94964	0.986413	0.997368	0.98895	0.979661
90	0.94964	0.986413	0.997368	0.98895	0.979661
91	0.94964	0.986413	0.997368	0.98895	0.979661
92	0.94964	0.986413	0.997368	0.98895	0.979661
93	0.94964	0.983696	0.997368	0.988161	0.978209
94	0.94964	0.983696	0.997368	0.988161	0.978209
95	0.94964	0.986413	0.997368	0.98895	0.979661
96	0.94964	0.986413	0.997368	0.98895	0.979661
97	0.94964	0.986413	0.997368	0.98895	0.979661
98	0.94964	0.986413	0.997368	0.98895	0.979661
99	0.94964	0.986413	0.997368	0.98895	0.979661
100	0.94964	0.986413	0.997368	0.98895	0.979661
101	0.94964	0.986413	0.997368	0.98895	0.979661
102	0.94964	0.986413	0.997368	0.98895	0.979661
103	0.94964	0.986413	0.997368	0.98895	0.979661
104	0.94964	0.986413	0.997368	0.98895	0.979661
105	0.94964	0.986413	0.997368	0.98895	0.979661
106	0.94964	0.986413	0.997368	0.98895	0.979661
107	0.94964	0.986413	0.997368	0.98895	0.979661
108	0.94964	0.986413	0.997368	0.98895	0.979661
109	0.94964	0.98913	0.997368	0.98974	0.981113
110	0.94964	0.98913	0.997368	0.98974	0.981113
111	0.94964	0.98913	0.997368	0.98974	0.981113
112	0.94964	0.986413	0.997368	0.98895	0.979661
113	0.94964	0.98913	0.997368	0.98974	0.981113
114	0.94964	0.986413	0.997368	0.98895	0.979661
115	0.956835	0.98913	0.997368	0.990529	0.982565
116	0.956835	0.986413	0.997368	0.98974	0.981112
117	0.956835	0.98913	0.997368	0.990529	0.982565
118	0.956835	0.98913	0.997368	0.990529	0.982565
119	0.94964	0.98913	0.998684	0.990529	0.982582
120	0.94964	0.986413	0.997368	0.98895	0.979661
121	0.956835	0.986413	0.997368	0.98974	0.981112
122	0.956835	0.986413	0.997368	0.98974	0.981112
123	0.956835	0.986413	0.997368	0.98974	0.981112
124	0.956835	0.986413	0.998684	0.990529	0.982582
125	0.956835	0.986413	0.998684	0.990529	0.982582
126	0.956835	0.986413	0.997368	0.98974	0.981112

127	0.956835	0.986413	0.997368	0.98974	0.981112
128	0.956835	0.986413	0.997368	0.98974	0.981112
129	0.956835	0.983696	0.997368	0.98895	0.979661
130	0.956835	0.983696	0.997368	0.98895	0.979661
131	0.956835	0.983696	0.997368	0.98895	0.979661
132	0.956835	0.986413	0.997368	0.98974	0.981112
133	0.956835	0.986413	0.997368	0.98974	0.981112
134	0.956835	0.986413	0.997368	0.98974	0.981112
135	0.956835	0.983696	0.998684	0.98974	0.981133
136	0.956835	0.983696	0.997368	0.98895	0.979661
137	0.956835	0.98913	0.998684	0.991318	0.984032
138	0.956835	0.98913	0.998684	0.991318	0.984032
139	0.956835	0.98913	1	0.992107	0.985503
140	0.956835	0.98913	1	0.992107	0.985503
141	0.956835	0.98913	1	0.992107	0.985503
142	0.956835	0.98913	1	0.992107	0.985503
143	0.956835	0.98913	1	0.992107	0.985503
144	0.956835	0.98913	1	0.992107	0.985503
145	0.956835	0.98913	1	0.992107	0.985503
146	0.956835	0.98913	1	0.992107	0.985503
147	0.956835	0.98913	1	0.992107	0.985503
148	0.956835	0.98913	1	0.992107	0.985503
149	0.956835	0.98913	1	0.992107	0.985503
150	0.956835	0.98913	1	0.992107	0.985503
151	0.956835	0.98913	1	0.992107	0.985503
152	0.956835	0.98913	1	0.992107	0.985503
153	0.956835	0.986413	1	0.991318	0.984055
154	0.956835	0.98913	1	0.992107	0.985503
155	0.956835	0.98913	1	0.992107	0.985503
156	0.956835	0.98913	1	0.992107	0.985503
157	0.956835	0.98913	1	0.992107	0.985503
158	0.956835	0.98913	1	0.992107	0.985503
159	0.956835	0.98913	1	0.992107	0.985503
160	0.956835	0.98913	1	0.992107	0.985503
161	0.956835	0.98913	1	0.992107	0.985503
162	0.956835	0.98913	1	0.992107	0.985503
163	0.956835	0.98913	1	0.992107	0.985503
164	0.956835	0.98913	1	0.992107	0.985503
165	0.956835	0.98913	1	0.992107	0.985503
166	0.956835	0.98913	1	0.992107	0.985503
167	0.956835	0.98913	1	0.992107	0.985503
168	0.956835	0.98913	1	0.992107	0.985503
169	0.956835	0.98913	1	0.992107	0.985503

170	0.956835	0.98913	1	0.992107	0.985503
171	0.956835	0.98913	1	0.992107	0.985503
172	0.956835	0.98913	1	0.992107	0.985503
173	0.956835	0.98913	1	0.992107	0.985503
174	0.956835	0.98913	0.998684	0.991318	0.984032
175	0.956835	0.98913	0.998684	0.991318	0.984032
176	0.956835	0.98913	0.998684	0.991318	0.984032
177	0.956835	0.98913	0.998684	0.991318	0.984032
178	0.956835	0.98913	0.998684	0.991318	0.984032
179	0.956835	0.98913	0.998684	0.991318	0.984032
180	0.956835	0.98913	0.998684	0.991318	0.984032
181	0.956835	0.98913	0.998684	0.991318	0.984032
182	0.956835	0.98913	0.998684	0.991318	0.984032
183	0.956835	0.98913	0.998684	0.991318	0.984032
184	0.956835	0.991848	0.998684	0.992107	0.985482
185	0.956835	0.991848	0.998684	0.992107	0.985482
186	0.956835	0.991848	0.998684	0.992107	0.985482
187	0.956835	0.991848	0.997368	0.991318	0.984017
188	0.956835	0.98913	0.998684	0.991318	0.984032
189	0.956835	0.98913	0.998684	0.991318	0.984032
190	0.956835	0.98913	0.997368	0.990529	0.982565
191	0.956835	0.98913	0.997368	0.990529	0.982565
192	0.956835	0.98913	0.997368	0.990529	0.982565
193	0.956835	0.98913	0.997368	0.990529	0.982565
194	0.956835	0.98913	0.997368	0.990529	0.982565
195	0.956835	0.991848	0.997368	0.991318	0.984017
196	0.956835	0.991848	0.997368	0.991318	0.984017
197	0.956835	0.991848	0.997368	0.991318	0.984017
198	0.956835	0.991848	0.997368	0.991318	0.984017
199	0.956835	0.991848	0.997368	0.991318	0.984017
200	0.956835	0.98913	0.997368	0.990529	0.982565
201	0.956835	0.98913	0.997368	0.990529	0.982565
202	0.956835	0.98913	0.997368	0.990529	0.982565
203	0.956835	0.98913	0.997368	0.990529	0.982565
204	0.956835	0.98913	0.997368	0.990529	0.982565
205	0.956835	0.98913	0.997368	0.990529	0.982565
206	0.956835	0.98913	0.997368	0.990529	0.982565
207	0.956835	0.98913	0.997368	0.990529	0.982565
208	0.956835	0.98913	0.997368	0.990529	0.982565
209	0.956835	0.98913	0.997368	0.990529	0.982565
210	0.956835	0.98913	0.997368	0.990529	0.982565
211	0.956835	0.98913	0.997368	0.990529	0.982565
212	0.956835	0.98913	0.997368	0.990529	0.982565

213	0.956835	0.98913	0.997368	0.990529	0.982565
214	0.956835	0.98913	0.997368	0.990529	0.982565
215	0.956835	0.98913	0.997368	0.990529	0.982565
216	0.956835	0.98913	0.997368	0.990529	0.982565
217	0.956835	0.98913	0.997368	0.990529	0.982565
218	0.956835	0.98913	0.997368	0.990529	0.982565
219	0.956835	0.98913	0.997368	0.990529	0.982565
220	0.956835	0.98913	0.997368	0.990529	0.982565
221	0.956835	0.98913	0.997368	0.990529	0.982565
222	0.956835	0.98913	0.997368	0.990529	0.982565
223	0.956835	0.98913	0.997368	0.990529	0.982565
224	0.956835	0.98913	0.997368	0.990529	0.982565
225	0.956835	0.98913	0.997368	0.990529	0.982565
226	0.956835	0.98913	0.997368	0.990529	0.982565
227	0.956835	0.98913	0.997368	0.990529	0.982565
228	0.956835	0.98913	0.997368	0.990529	0.982565
229	0.956835	0.98913	0.997368	0.990529	0.982565
230	0.956835	0.98913	0.997368	0.990529	0.982565
231	0.956835	0.98913	0.997368	0.990529	0.982565
232	0.956835	0.98913	0.997368	0.990529	0.982565
233	0.956835	0.98913	0.997368	0.990529	0.982565
234	0.956835	0.98913	0.997368	0.990529	0.982565
235	0.956835	0.98913	0.997368	0.990529	0.982565
236	0.956835	0.98913	0.997368	0.990529	0.982565
237	0.956835	0.98913	0.997368	0.990529	0.982565
238	0.956835	0.98913	0.997368	0.990529	0.982565
239	0.964029	0.98913	0.998684	0.992107	0.985482
240	0.964029	0.98913	0.998684	0.992107	0.985482
241	0.964029	0.98913	0.998684	0.992107	0.985482
242	0.964029	0.98913	0.998684	0.992107	0.985482
243	0.964029	0.98913	0.998684	0.992107	0.985482
244	0.964029	0.98913	0.998684	0.992107	0.985482
245	0.964029	0.98913	0.998684	0.992107	0.985482
246	0.964029	0.98913	0.998684	0.992107	0.985482
247	0.964029	0.98913	0.998684	0.992107	0.985482
248	0.964029	0.98913	0.998684	0.992107	0.985482
249	0.964029	0.98913	0.998684	0.992107	0.985482
250	0.964029	0.98913	0.998684	0.992107	0.985482
251	0.964029	0.98913	0.998684	0.992107	0.985482
252	0.964029	0.98913	0.998684	0.992107	0.985482
253	0.964029	0.98913	0.998684	0.992107	0.985482
254	0.964029	0.98913	0.997368	0.991318	0.984018
255	0.964029	0.98913	0.998684	0.992107	0.985482

256	0.971223	0.98913	0.997368	0.992107	0.985472
257	0.971223	0.98913	0.998684	0.992897	0.986933
258	0.964029	0.98913	0.997368	0.991318	0.984018
259	0.964029	0.98913	0.997368	0.991318	0.984018
260	0.964029	0.98913	0.997368	0.991318	0.984018
261	0.964029	0.98913	0.998684	0.992107	0.985482
262	0.964029	0.98913	0.998684	0.992107	0.985482
263	0.964029	0.98913	0.998684	0.992107	0.985482
264	0.964029	0.98913	0.997368	0.991318	0.984018
265	0.964029	0.98913	0.998684	0.992107	0.985482
266	0.964029	0.98913	0.998684	0.992107	0.985482
267	0.964029	0.98913	0.998684	0.992107	0.985482
268	0.964029	0.98913	0.998684	0.992107	0.985482
269	0.964029	0.98913	0.998684	0.992107	0.985482
270	0.964029	0.98913	0.998684	0.992107	0.985482
271	0.964029	0.98913	0.998684	0.992107	0.985482
272	0.964029	0.98913	0.998684	0.992107	0.985482
273	0.964029	0.98913	0.998684	0.992107	0.985482
274	0.964029	0.98913	0.998684	0.992107	0.985482
275	0.964029	0.98913	0.998684	0.992107	0.985482
276	0.964029	0.991848	0.998684	0.992897	0.986933
277	0.964029	0.991848	0.998684	0.992897	0.986933
278	0.971223	0.991848	0.998684	0.993686	0.988384
279	0.971223	0.991848	0.998684	0.993686	0.988384
280	0.964029	0.991848	0.998684	0.992897	0.986933
281	0.964029	0.991848	0.998684	0.992897	0.986933
282	0.964029	0.991848	0.998684	0.992897	0.986933
283	0.964029	0.991848	0.998684	0.992897	0.986933
284	0.964029	0.991848	0.998684	0.992897	0.986933
285	0.964029	0.991848	0.998684	0.992897	0.986933
286	0.964029	0.991848	0.998684	0.992897	0.986933
287	0.964029	0.991848	0.998684	0.992897	0.986933
288	0.964029	0.991848	0.998684	0.992897	0.986933
289	0.964029	0.991848	0.998684	0.992897	0.986933
290	0.964029	0.991848	0.998684	0.992897	0.986933
291	0.964029	0.991848	0.998684	0.992897	0.986933
292	0.964029	0.991848	0.998684	0.992897	0.986933
293	0.964029	0.991848	0.998684	0.992897	0.986933
294	0.964029	0.991848	0.998684	0.992897	0.986933
295	0.964029	0.991848	0.998684	0.992897	0.986933
296	0.964029	0.991848	0.998684	0.992897	0.986933
297	0.964029	0.991848	0.998684	0.992897	0.986933
298	0.964029	0.991848	0.998684	0.992897	0.986933

299	0.964029	0.991848	0.998684	0.992897	0.986933
300	0.964029	0.991848	0.998684	0.992897	0.986933
301	0.964029	0.991848	0.998684	0.992897	0.986933
302	0.964029	0.991848	0.998684	0.992897	0.986933
303	0.964029	0.991848	0.998684	0.992897	0.986933
304	0.964029	0.991848	0.998684	0.992897	0.986933
305	0.964029	0.991848	0.998684	0.992897	0.986933
306	0.964029	0.991848	0.998684	0.992897	0.986933
307	0.964029	0.991848	0.998684	0.992897	0.986933
308	0.964029	0.991848	0.998684	0.992897	0.986933
309	0.964029	0.991848	0.998684	0.992897	0.986933
310	0.964029	0.991848	0.998684	0.992897	0.986933
311	0.964029	0.991848	0.998684	0.992897	0.986933
312	0.964029	0.991848	0.998684	0.992897	0.986933
313	0.964029	0.991848	0.998684	0.992897	0.986933
314	0.964029	0.991848	0.998684	0.992897	0.986933
315	0.964029	0.991848	0.998684	0.992897	0.986933
316	0.964029	0.991848	0.998684	0.992897	0.986933
317	0.964029	0.991848	0.998684	0.992897	0.986933
318	0.964029	0.991848	0.998684	0.992897	0.986933
319	0.964029	0.991848	0.998684	0.992897	0.986933
320	0.964029	0.991848	0.998684	0.992897	0.986933
321	0.964029	0.991848	0.998684	0.992897	0.986933
322	0.964029	0.991848	0.998684	0.992897	0.986933
323	0.964029	0.991848	0.998684	0.992897	0.986933
324	0.971223	0.991848	0.998684	0.993686	0.988384
325	0.964029	0.991848	0.998684	0.992897	0.986933
326	0.964029	0.991848	0.998684	0.992897	0.986933
327	0.964029	0.991848	0.998684	0.992897	0.986933
328	0.964029	0.991848	0.998684	0.992897	0.986933
329	0.964029	0.991848	0.998684	0.992897	0.986933
330	0.964029	0.991848	0.998684	0.992897	0.986933
331	0.964029	0.991848	0.998684	0.992897	0.986933
332	0.964029	0.991848	0.998684	0.992897	0.986933
333	0.971223	0.991848	0.998684	0.993686	0.988384
334	0.971223	0.991848	0.998684	0.993686	0.988384
335	0.971223	0.991848	0.998684	0.993686	0.988384
336	0.971223	0.991848	0.998684	0.993686	0.988384
337	0.971223	0.991848	0.998684	0.993686	0.988384
338	0.971223	0.991848	0.998684	0.993686	0.988384
339	0.971223	0.991848	0.998684	0.993686	0.988384
340	0.971223	0.991848	0.998684	0.993686	0.988384
341	0.971223	0.991848	0.998684	0.993686	0.988384

342	0.971223	0.991848	0.998684	0.993686	0.988384
343	0.971223	0.991848	0.998684	0.993686	0.988384
344	0.978417	0.991848	0.998684	0.994475	0.989835
345	0.978417	0.991848	0.998684	0.994475	0.989835
346	0.978417	0.991848	0.998684	0.994475	0.989835
347	0.978417	0.991848	0.998684	0.994475	0.989835
348	0.978417	0.991848	0.998684	0.994475	0.989835
349	0.971223	0.991848	0.998684	0.993686	0.988384
350	0.971223	0.991848	0.998684	0.993686	0.988384
351	0.971223	0.991848	0.998684	0.993686	0.988384
352	0.971223	0.991848	0.998684	0.993686	0.988384
353	0.971223	0.991848	0.998684	0.993686	0.988384
354	0.971223	0.991848	0.998684	0.993686	0.988384
355	0.971223	0.991848	1	0.994475	0.989848
356	0.971223	0.991848	1	0.994475	0.989848
357	0.971223	0.991848	1	0.994475	0.989848
358	0.971223	0.991848	0.998684	0.993686	0.988384
359	0.971223	0.991848	0.998684	0.993686	0.988384
360	0.971223	0.991848	0.998684	0.993686	0.988384
361	0.971223	0.991848	0.998684	0.993686	0.988384
362	0.971223	0.991848	0.998684	0.993686	0.988384
363	0.971223	0.991848	0.998684	0.993686	0.988384
364	0.971223	0.991848	0.998684	0.993686	0.988384
365	0.971223	0.991848	0.998684	0.993686	0.988384
366	0.971223	0.991848	1	0.994475	0.989848
367	0.971223	0.991848	1	0.994475	0.989848
368	0.971223	0.991848	1	0.994475	0.989848
369	0.971223	0.991848	1	0.994475	0.989848
370	0.971223	0.991848	1	0.994475	0.989848
371	0.971223	0.991848	1	0.994475	0.989848
372	0.971223	0.991848	1	0.994475	0.989848
373	0.971223	0.991848	1	0.994475	0.989848
374	0.971223	0.991848	1	0.994475	0.989848
375	0.971223	0.991848	1	0.994475	0.989848
376	0.971223	0.991848	1	0.994475	0.989848
377	0.971223	0.991848	1	0.994475	0.989848
378	0.971223	0.991848	1	0.994475	0.989848
379	0.971223	0.991848	1	0.994475	0.989848
380	0.971223	0.991848	1	0.994475	0.989848
381	0.971223	0.991848	1	0.994475	0.989848
382	0.978417	0.991848	1	0.995264	0.991298
383	0.971223	0.991848	1	0.994475	0.989848
384	0.971223	0.991848	1	0.994475	0.989848

385	0.971223	0.991848	1	0.994475	0.989848
386	0.971223	0.991848	1	0.994475	0.989848
387	0.971223	0.991848	1	0.994475	0.989848
388	0.971223	0.991848	1	0.994475	0.989848
389	0.971223	0.991848	1	0.994475	0.989848
390	0.978417	0.991848	1	0.995264	0.991298
391	0.978417	0.991848	1	0.995264	0.991298
392	0.978417	0.991848	1	0.995264	0.991298
393	0.978417	0.991848	1	0.995264	0.991298
394	0.978417	0.991848	1	0.995264	0.991298
395	0.978417	0.991848	1	0.995264	0.991298
396	0.978417	0.991848	1	0.995264	0.991298
397	0.978417	0.991848	1	0.995264	0.991298
398	0.978417	0.991848	1	0.995264	0.991298
399	0.978417	0.991848	1	0.995264	0.991298
400	0.978417	0.991848	1	0.995264	0.991298
401	0.978417	0.991848	1	0.995264	0.991298
402	0.978417	0.991848	1	0.995264	0.991298
403	0.978417	0.991848	1	0.995264	0.991298
404	0.978417	0.991848	1	0.995264	0.991298
405	0.978417	0.991848	1	0.995264	0.991298
406	0.978417	0.991848	0.998684	0.994475	0.989835
407	0.978417	0.991848	0.998684	0.994475	0.989835
408	0.978417	0.991848	0.998684	0.994475	0.989835
409	0.978417	0.991848	0.998684	0.994475	0.989835
410	0.978417	0.991848	0.998684	0.994475	0.989835
411	0.978417	0.991848	0.998684	0.994475	0.989835
412	0.978417	0.991848	0.998684	0.994475	0.989835
413	0.978417	0.991848	0.998684	0.994475	0.989835
414	0.978417	0.991848	0.998684	0.994475	0.989835
415	0.978417	0.991848	0.998684	0.994475	0.989835
416	0.978417	0.991848	0.998684	0.994475	0.989835
417	0.978417	0.991848	0.998684	0.994475	0.989835
418	0.978417	0.991848	0.998684	0.994475	0.989835
419	0.978417	0.991848	0.998684	0.994475	0.989835
420	0.978417	0.991848	0.998684	0.994475	0.989835
421	0.978417	0.991848	0.998684	0.994475	0.989835
422	0.978417	0.991848	0.998684	0.994475	0.989835
423	0.978417	0.991848	0.998684	0.994475	0.989835
424	0.978417	0.991848	0.998684	0.994475	0.989835
425	0.978417	0.991848	0.998684	0.994475	0.989835
426	0.978417	0.991848	0.998684	0.994475	0.989835
427	0.978417	0.991848	0.998684	0.994475	0.989835

428	0.978417	0.991848	0.998684	0.994475	0.989835
429	0.978417	0.991848	0.998684	0.994475	0.989835
430	0.978417	0.991848	0.998684	0.994475	0.989835
431	0.978417	0.991848	0.998684	0.994475	0.989835
432	0.978417	0.991848	0.998684	0.994475	0.989835
433	0.978417	0.991848	0.998684	0.994475	0.989835
434	0.978417	0.991848	0.998684	0.994475	0.989835
435	0.978417	0.991848	0.998684	0.994475	0.989835
436	0.978417	0.991848	0.998684	0.994475	0.989835
437	0.978417	0.991848	0.998684	0.994475	0.989835
438	0.978417	0.991848	0.998684	0.994475	0.989835
439	0.978417	0.991848	0.998684	0.994475	0.989835
440	0.978417	0.991848	0.998684	0.994475	0.989835
441	0.978417	0.991848	0.998684	0.994475	0.989835
442	0.978417	0.991848	0.998684	0.994475	0.989835
443	0.978417	0.991848	0.998684	0.994475	0.989835
444	0.978417	0.991848	0.998684	0.994475	0.989835
445	0.978417	0.991848	0.998684	0.994475	0.989835
446	0.978417	0.991848	0.998684	0.994475	0.989835
447	0.978417	0.991848	0.998684	0.994475	0.989835
448	0.978417	0.991848	0.998684	0.994475	0.989835
449	0.978417	0.991848	0.998684	0.994475	0.989835
450	0.978417	0.991848	0.998684	0.994475	0.989835
451	0.978417	0.991848	0.998684	0.994475	0.989835
452	0.978417	0.991848	1	0.995264	0.991298
453	0.978417	0.991848	1	0.995264	0.991298
454	0.985612	0.991848	1	0.996054	0.992748
455	0.985612	0.991848	1	0.996054	0.992748
456	0.985612	0.991848	1	0.996054	0.992748
457	0.985612	0.991848	1	0.996054	0.992748
458	0.985612	0.991848	1	0.996054	0.992748
459	0.985612	0.991848	1	0.996054	0.992748
460	0.985612	0.991848	1	0.996054	0.992748
461	0.985612	0.991848	1	0.996054	0.992748
462	0.985612	0.991848	1	0.996054	0.992748
463	0.985612	0.991848	1	0.996054	0.992748
464	0.985612	0.991848	1	0.996054	0.992748
465	0.985612	0.991848	1	0.996054	0.992748
466	0.985612	0.991848	1	0.996054	0.992748
467	0.985612	0.991848	1	0.996054	0.992748
468	0.985612	0.991848	1	0.996054	0.992748
469	0.985612	0.991848	1	0.996054	0.992748
470	0.985612	0.991848	1	0.996054	0.992748

471	0.985612	0.991848	1	0.996054	0.992748
472	0.985612	0.991848	1	0.996054	0.992748
473	0.985612	0.991848	1	0.996054	0.992748
474	0.985612	0.991848	1	0.996054	0.992748
475	0.985612	0.991848	1	0.996054	0.992748
476	0.985612	0.991848	1	0.996054	0.992748
477	0.985612	0.991848	1	0.996054	0.992748
478	0.985612	0.991848	1	0.996054	0.992748
479	0.985612	0.991848	1	0.996054	0.992748
480	0.985612	0.991848	1	0.996054	0.992748
481	0.985612	0.991848	1	0.996054	0.992748
482	0.985612	0.991848	1	0.996054	0.992748
483	0.985612	0.991848	1	0.996054	0.992748
484	0.985612	0.991848	1	0.996054	0.992748
485	0.985612	0.991848	1	0.996054	0.992748
486	0.985612	0.991848	1	0.996054	0.992748
487	0.985612	0.991848	1	0.996054	0.992748
488	0.985612	0.991848	1	0.996054	0.992748
489	0.985612	0.991848	1	0.996054	0.992748
490	0.985612	0.991848	1	0.996054	0.992748
491	0.985612	0.991848	1	0.996054	0.992748
492	0.985612	0.991848	1	0.996054	0.992748
493	0.985612	0.991848	1	0.996054	0.992748
494	0.985612	0.991848	1	0.996054	0.992748
495	0.985612	0.991848	1	0.996054	0.992748
496	0.985612	0.991848	1	0.996054	0.992748
497	0.985612	0.991848	1	0.996054	0.992748
498	0.985612	0.991848	1	0.996054	0.992748
499	0.985612	0.991848	1	0.996054	0.992748
500	0.985612	0.991848	1	0.996054	0.992748
501	0.985612	0.991848	1	0.996054	0.992748
502	0.985612	0.991848	1	0.996054	0.992748
503	0.985612	0.991848	1	0.996054	0.992748
504	0.985612	0.991848	1	0.996054	0.992748
505	0.985612	0.991848	1	0.996054	0.992748
506	0.985612	0.991848	1	0.996054	0.992748
507	0.985612	0.991848	1	0.996054	0.992748
508	0.985612	0.991848	1	0.996054	0.992748
509	0.985612	0.991848	1	0.996054	0.992748
510	0.985612	0.991848	1	0.996054	0.992748
511	0.985612	0.991848	1	0.996054	0.992748
512	0.985612	0.991848	1	0.996054	0.992748
513	0.985612	0.991848	1	0.996054	0.992748

514	0.985612	0.991848	1	0.996054	0.992748
515	0.985612	0.991848	1	0.996054	0.992748
516	0.985612	0.991848	1	0.996054	0.992748
517	0.985612	0.991848	1	0.996054	0.992748
518	0.985612	0.991848	1	0.996054	0.992748
519	0.985612	0.991848	1	0.996054	0.992748
520	0.985612	0.991848	1	0.996054	0.992748
521	0.985612	0.991848	1	0.996054	0.992748
522	0.985612	0.991848	1	0.996054	0.992748
523	0.985612	0.991848	1	0.996054	0.992748
524	0.985612	0.991848	1	0.996054	0.992748
525	0.985612	0.991848	1	0.996054	0.992748
526	0.985612	0.991848	1	0.996054	0.992748
527	0.985612	0.991848	1	0.996054	0.992748
528	0.985612	0.991848	1	0.996054	0.992748
529	0.985612	0.991848	1	0.996054	0.992748
530	0.985612	0.991848	1	0.996054	0.992748
531	0.985612	0.991848	1	0.996054	0.992748
532	0.985612	0.991848	1	0.996054	0.992748
533	0.985612	0.991848	1	0.996054	0.992748
534	0.985612	0.991848	1	0.996054	0.992748
535	0.985612	0.991848	1	0.996054	0.992748
536	0.985612	0.991848	1	0.996054	0.992748
537	0.985612	0.991848	1	0.996054	0.992748
538	0.985612	0.991848	1	0.996054	0.992748
539	0.985612	0.991848	1	0.996054	0.992748
540	0.985612	0.991848	1	0.996054	0.992748
541	0.985612	0.991848	1	0.996054	0.992748
542	0.985612	0.991848	1	0.996054	0.992748
543	0.985612	0.991848	1	0.996054	0.992748
544	0.985612	0.991848	1	0.996054	0.992748
545	0.985612	0.991848	1	0.996054	0.992748
546	0.985612	0.991848	1	0.996054	0.992748
547	0.985612	0.991848	1	0.996054	0.992748
548	0.985612	0.991848	1	0.996054	0.992748
549	0.985612	0.991848	1	0.996054	0.992748
550	0.985612	0.991848	1	0.996054	0.992748
551	0.985612	0.991848	1	0.996054	0.992748
552	0.985612	0.991848	1	0.996054	0.992748
553	0.985612	0.991848	1	0.996054	0.992748
554	0.985612	0.991848	1	0.996054	0.992748
555	0.985612	0.991848	1	0.996054	0.992748
556	0.978417	0.991848	1	0.995264	0.991298

557	0.978417	0.991848	1	0.995264	0.991298
558	0.978417	0.991848	1	0.995264	0.991298
559	0.978417	0.991848	1	0.995264	0.991298
560	0.985612	0.991848	1	0.996054	0.992748
561	0.985612	0.991848	1	0.996054	0.992748
562	0.985612	0.991848	1	0.996054	0.992748
563	0.985612	0.991848	1	0.996054	0.992748
564	0.985612	0.991848	1	0.996054	0.992748
565	0.985612	0.991848	1	0.996054	0.992748
566	0.985612	0.991848	1	0.996054	0.992748
567	0.985612	0.991848	1	0.996054	0.992748
568	0.985612	0.991848	1	0.996054	0.992748
569	0.985612	0.991848	1	0.996054	0.992748
570	0.985612	0.991848	1	0.996054	0.992748
571	0.985612	0.991848	1	0.996054	0.992748
572	0.985612	0.991848	1	0.996054	0.992748
573	0.985612	0.991848	1	0.996054	0.992748
574	0.985612	0.991848	1	0.996054	0.992748
575	0.985612	0.991848	1	0.996054	0.992748
576	0.985612	0.991848	1	0.996054	0.992748
577	0.985612	0.991848	1	0.996054	0.992748
578	0.978417	0.991848	1	0.995264	0.991298
579	0.985612	0.991848	1	0.996054	0.992748
580	0.985612	0.991848	1	0.996054	0.992748
581	0.978417	0.991848	1	0.995264	0.991298
582	0.985612	0.991848	1	0.996054	0.992748
583	0.985612	0.991848	1	0.996054	0.992748
584	0.985612	0.991848	1	0.996054	0.992748
585	0.985612	0.991848	1	0.996054	0.992748
586	0.985612	0.991848	1	0.996054	0.992748
587	0.985612	0.991848	1	0.996054	0.992748
588	0.978417	0.991848	1	0.995264	0.991298
589	0.978417	0.991848	1	0.995264	0.991298
590	0.978417	0.991848	1	0.995264	0.991298
591	0.978417	0.991848	1	0.995264	0.991298
592	0.971223	0.991848	1	0.994475	0.989848
593	0.971223	0.991848	1	0.994475	0.989848
594	0.971223	0.991848	1	0.994475	0.989848
595	0.971223	0.991848	1	0.994475	0.989848
596	0.971223	0.991848	1	0.994475	0.989848
597	0.971223	0.991848	1	0.994475	0.989848
598	0.971223	0.991848	1	0.994475	0.989848
599	0.971223	0.991848	1	0.994475	0.989848

600	0.971223	0.991848	1	0.994475	0.989848
601	0.971223	0.991848	1	0.994475	0.989848
602	0.971223	0.991848	1	0.994475	0.989848
603	0.971223	0.991848	1	0.994475	0.989848
604	0.971223	0.991848	1	0.994475	0.989848
605	0.971223	0.991848	1	0.994475	0.989848
606	0.971223	0.991848	1	0.994475	0.989848
607	0.971223	0.991848	1	0.994475	0.989848
608	0.971223	0.991848	1	0.994475	0.989848
609	0.971223	0.991848	1	0.994475	0.989848
610	0.971223	0.991848	1	0.994475	0.989848
611	0.971223	0.991848	1	0.994475	0.989848
612	0.971223	0.991848	1	0.994475	0.989848
613	0.971223	0.98913	1	0.993686	0.9884
614	0.971223	0.98913	1	0.993686	0.9884
615	0.971223	0.98913	1	0.993686	0.9884
616	0.971223	0.98913	1	0.993686	0.9884
617	0.971223	0.98913	1	0.993686	0.9884
618	0.971223	0.98913	1	0.993686	0.9884
619	0.971223	0.98913	1	0.993686	0.9884
620	0.971223	0.98913	1	0.993686	0.9884
621	0.971223	0.98913	1	0.993686	0.9884
622	0.971223	0.98913	1	0.993686	0.9884
623	0.971223	0.98913	1	0.993686	0.9884
624	0.971223	0.98913	1	0.993686	0.9884
625	0.971223	0.98913	1	0.993686	0.9884
626	0.971223	0.98913	1	0.993686	0.9884
627	0.971223	0.98913	1	0.993686	0.9884
628	0.971223	0.98913	1	0.993686	0.9884
629	0.971223	0.98913	1	0.993686	0.9884
630	0.971223	0.98913	1	0.993686	0.9884
631	0.971223	0.98913	1	0.993686	0.9884
632	0.971223	0.98913	1	0.993686	0.9884
633	0.971223	0.98913	1	0.993686	0.9884
634	0.971223	0.98913	1	0.993686	0.9884
635	0.971223	0.98913	1	0.993686	0.9884
636	0.971223	0.98913	1	0.993686	0.9884
637	0.971223	0.98913	1	0.993686	0.9884
638	0.971223	0.98913	1	0.993686	0.9884
639	0.971223	0.98913	1	0.993686	0.9884
640	0.971223	0.98913	1	0.993686	0.9884
641	0.971223	0.98913	1	0.993686	0.9884
642	0.971223	0.98913	1	0.993686	0.9884

643	0.971223	0.98913	1	0.993686	0.9884
644	0.971223	0.98913	1	0.993686	0.9884
645	0.971223	0.98913	1	0.993686	0.9884
646	0.971223	0.98913	1	0.993686	0.9884
647	0.971223	0.98913	1	0.993686	0.9884
648	0.971223	0.98913	1	0.993686	0.9884
649	0.971223	0.98913	1	0.993686	0.9884
650	0.971223	0.98913	1	0.993686	0.9884
651	0.971223	0.98913	1	0.993686	0.9884
652	0.971223	0.98913	1	0.993686	0.9884
653	0.971223	0.98913	1	0.993686	0.9884
654	0.971223	0.98913	1	0.993686	0.9884
655	0.971223	0.98913	1	0.993686	0.9884
656	0.971223	0.98913	1	0.993686	0.9884
657	0.971223	0.98913	1	0.993686	0.9884
658	0.971223	0.98913	1	0.993686	0.9884
659	0.971223	0.98913	1	0.993686	0.9884
660	0.971223	0.98913	1	0.993686	0.9884
661	0.978417	0.98913	1	0.994475	0.98985
662	0.978417	0.98913	1	0.994475	0.98985
663	0.978417	0.98913	1	0.994475	0.98985
664	0.978417	0.98913	1	0.994475	0.98985
665	0.978417	0.98913	1	0.994475	0.98985
666	0.978417	0.98913	1	0.994475	0.98985
667	0.978417	0.98913	1	0.994475	0.98985
668	0.978417	0.98913	1	0.994475	0.98985
669	0.978417	0.98913	1	0.994475	0.98985
670	0.978417	0.98913	1	0.994475	0.98985
671	0.971223	0.98913	1	0.993686	0.9884
672	0.971223	0.98913	1	0.993686	0.9884
673	0.971223	0.98913	1	0.993686	0.9884
674	0.971223	0.98913	1	0.993686	0.9884
675	0.971223	0.98913	1	0.993686	0.9884
676	0.971223	0.98913	1	0.993686	0.9884
677	0.971223	0.98913	1	0.993686	0.9884
678	0.971223	0.98913	1	0.993686	0.9884
679	0.971223	0.98913	1	0.993686	0.9884
680	0.971223	0.98913	1	0.993686	0.9884
681	0.971223	0.98913	1	0.993686	0.9884
682	0.978417	0.98913	1	0.994475	0.98985
683	0.978417	0.98913	1	0.994475	0.98985
684	0.978417	0.98913	1	0.994475	0.98985
685	0.978417	0.98913	1	0.994475	0.98985

686	0.971223	0.98913	1	0.993686	0.9884
687	0.971223	0.98913	1	0.993686	0.9884
688	0.971223	0.98913	1	0.993686	0.9884
689	0.971223	0.98913	1	0.993686	0.9884
690	0.971223	0.98913	1	0.993686	0.9884
691	0.971223	0.98913	1	0.993686	0.9884
692	0.971223	0.98913	1	0.993686	0.9884
693	0.971223	0.98913	1	0.993686	0.9884
694	0.971223	0.991848	1	0.994475	0.989848
695	0.971223	0.991848	1	0.994475	0.989848
696	0.971223	0.991848	1	0.994475	0.989848
697	0.971223	0.991848	1	0.994475	0.989848
698	0.971223	0.991848	1	0.994475	0.989848
699	0.971223	0.991848	1	0.994475	0.989848
700	0.971223	0.991848	1	0.994475	0.989848
701	0.971223	0.991848	1	0.994475	0.989848
702	0.971223	0.991848	1	0.994475	0.989848
703	0.971223	0.991848	1	0.994475	0.989848
704	0.971223	0.991848	1	0.994475	0.989848
705	0.971223	0.991848	1	0.994475	0.989848
706	0.971223	0.991848	1	0.994475	0.989848
707	0.971223	0.991848	1	0.994475	0.989848
708	0.971223	0.991848	1	0.994475	0.989848
709	0.971223	0.991848	1	0.994475	0.989848
710	0.971223	0.991848	1	0.994475	0.989848
711	0.971223	0.991848	1	0.994475	0.989848
712	0.971223	0.991848	1	0.994475	0.989848
713	0.971223	0.991848	1	0.994475	0.989848
714	0.971223	0.991848	1	0.994475	0.989848
715	0.971223	0.991848	1	0.994475	0.989848
716	0.971223	0.991848	1	0.994475	0.989848
717	0.971223	0.991848	1	0.994475	0.989848
718	0.971223	0.991848	1	0.994475	0.989848
719	0.971223	0.991848	1	0.994475	0.989848
720	0.971223	0.991848	1	0.994475	0.989848
721	0.971223	0.991848	1	0.994475	0.989848
722	0.971223	0.991848	1	0.994475	0.989848
723	0.971223	0.991848	1	0.994475	0.989848
724	0.971223	0.991848	1	0.994475	0.989848
725	0.971223	0.991848	1	0.994475	0.989848
726	0.971223	0.991848	1	0.994475	0.989848
727	0.971223	0.991848	1	0.994475	0.989848
728	0.971223	0.991848	1	0.994475	0.989848

729	0.971223	0.991848	1	0.994475	0.989848
730	0.971223	0.991848	1	0.994475	0.989848
731	0.971223	0.991848	1	0.994475	0.989848
732	0.971223	0.991848	1	0.994475	0.989848
733	0.971223	0.991848	1	0.994475	0.989848
734	0.971223	0.991848	1	0.994475	0.989848
735	0.971223	0.991848	1	0.994475	0.989848
736	0.971223	0.991848	1	0.994475	0.989848
737	0.971223	0.991848	1	0.994475	0.989848
738	0.971223	0.991848	1	0.994475	0.989848
739	0.971223	0.991848	1	0.994475	0.989848
740	0.971223	0.991848	1	0.994475	0.989848
741	0.971223	0.991848	1	0.994475	0.989848
742	0.971223	0.991848	1	0.994475	0.989848
743	0.971223	0.991848	1	0.994475	0.989848
744	0.971223	0.991848	1	0.994475	0.989848
745	0.971223	0.991848	1	0.994475	0.989848
746	0.971223	0.991848	1	0.994475	0.989848
747	0.971223	0.991848	1	0.994475	0.989848
748	0.971223	0.991848	1	0.994475	0.989848
749	0.971223	0.991848	1	0.994475	0.989848
750	0.971223	0.991848	1	0.994475	0.989848
751	0.971223	0.991848	1	0.994475	0.989848
752	0.971223	0.991848	1	0.994475	0.989848
753	0.971223	0.991848	1	0.994475	0.989848
754	0.971223	0.991848	1	0.994475	0.989848
755	0.971223	0.991848	1	0.994475	0.989848
756	0.971223	0.991848	1	0.994475	0.989848
757	0.971223	0.991848	1	0.994475	0.989848
758	0.971223	0.991848	1	0.994475	0.989848
759	0.971223	0.991848	1	0.994475	0.989848
760	0.971223	0.991848	1	0.994475	0.989848
761	0.971223	0.991848	1	0.994475	0.989848
762	0.971223	0.991848	1	0.994475	0.989848
763	0.971223	0.991848	1	0.994475	0.989848
764	0.971223	0.991848	1	0.994475	0.989848
765	0.978417	0.991848	1	0.995264	0.991298
766	0.978417	0.991848	1	0.995264	0.991298
767	0.978417	0.991848	1	0.995264	0.991298
768	0.978417	0.991848	1	0.995264	0.991298
769	0.978417	0.991848	1	0.995264	0.991298
770	0.978417	0.991848	1	0.995264	0.991298
771	0.978417	0.991848	1	0.995264	0.991298

772	0.978417	0.991848	1	0.995264	0.991298
773	0.978417	0.991848	1	0.995264	0.991298
774	0.978417	0.991848	1	0.995264	0.991298
775	0.978417	0.991848	1	0.995264	0.991298
776	0.978417	0.991848	1	0.995264	0.991298
777	0.978417	0.991848	1	0.995264	0.991298
778	0.978417	0.991848	1	0.995264	0.991298
779	0.978417	0.991848	1	0.995264	0.991298
780	0.978417	0.991848	1	0.995264	0.991298
781	0.978417	0.991848	1	0.995264	0.991298
782	0.978417	0.991848	1	0.995264	0.991298
783	0.978417	0.991848	1	0.995264	0.991298
784	0.978417	0.991848	1	0.995264	0.991298
785	0.978417	0.991848	1	0.995264	0.991298
786	0.978417	0.991848	1	0.995264	0.991298
787	0.978417	0.991848	1	0.995264	0.991298
788	0.971223	0.991848	1	0.994475	0.989848
789	0.971223	0.991848	1	0.994475	0.989848
790	0.971223	0.991848	1	0.994475	0.989848
791	0.971223	0.991848	1	0.994475	0.989848
792	0.971223	0.991848	1	0.994475	0.989848
793	0.971223	0.991848	1	0.994475	0.989848
794	0.971223	0.991848	1	0.994475	0.989848
795	0.971223	0.991848	1	0.994475	0.989848
796	0.971223	0.991848	1	0.994475	0.989848
797	0.971223	0.991848	1	0.994475	0.989848
798	0.971223	0.991848	1	0.994475	0.989848
799	0.971223	0.991848	1	0.994475	0.989848
800	0.971223	0.991848	1	0.994475	0.989848
801	0.971223	0.991848	1	0.994475	0.989848
802	0.978417	0.991848	1	0.995264	0.991298
803	0.971223	0.991848	1	0.994475	0.989848
804	0.971223	0.991848	1	0.994475	0.989848
805	0.971223	0.991848	1	0.994475	0.989848
806	0.971223	0.991848	1	0.994475	0.989848
807	0.971223	0.991848	1	0.994475	0.989848
808	0.971223	0.991848	1	0.994475	0.989848
809	0.971223	0.991848	1	0.994475	0.989848
810	0.971223	0.991848	1	0.994475	0.989848
811	0.971223	0.991848	1	0.994475	0.989848
812	0.971223	0.991848	1	0.994475	0.989848
813	0.971223	0.991848	1	0.994475	0.989848
814	0.971223	0.991848	1	0.994475	0.989848

815	0.971223	0.991848	1	0.994475	0.989848
816	0.971223	0.991848	1	0.994475	0.989848
817	0.971223	0.991848	1	0.994475	0.989848
818	0.971223	0.991848	1	0.994475	0.989848
819	0.971223	0.991848	1	0.994475	0.989848
820	0.971223	0.991848	1	0.994475	0.989848
821	0.971223	0.991848	1	0.994475	0.989848
822	0.971223	0.991848	1	0.994475	0.989848
823	0.971223	0.991848	1	0.994475	0.989848
824	0.971223	0.991848	1	0.994475	0.989848
825	0.971223	0.991848	1	0.994475	0.989848
826	0.971223	0.991848	1	0.994475	0.989848
827	0.971223	0.991848	1	0.994475	0.989848
828	0.971223	0.991848	1	0.994475	0.989848
829	0.971223	0.991848	1	0.994475	0.989848
830	0.971223	0.991848	1	0.994475	0.989848
831	0.971223	0.991848	1	0.994475	0.989848
832	0.971223	0.991848	1	0.994475	0.989848
833	0.971223	0.991848	1	0.994475	0.989848
834	0.971223	0.991848	1	0.994475	0.989848
835	0.971223	0.991848	1	0.994475	0.989848
836	0.971223	0.991848	1	0.994475	0.989848
837	0.971223	0.991848	1	0.994475	0.989848
838	0.971223	0.991848	1	0.994475	0.989848
839	0.971223	0.991848	1	0.994475	0.989848
840	0.971223	0.991848	1	0.994475	0.989848
841	0.971223	0.991848	1	0.994475	0.989848
842	0.971223	0.991848	1	0.994475	0.989848
843	0.971223	0.991848	1	0.994475	0.989848
844	0.971223	0.991848	1	0.994475	0.989848
845	0.971223	0.991848	1	0.994475	0.989848
846	0.971223	0.991848	1	0.994475	0.989848
847	0.971223	0.991848	1	0.994475	0.989848
848	0.971223	0.991848	1	0.994475	0.989848
849	0.971223	0.991848	1	0.994475	0.989848
850	0.971223	0.991848	1	0.994475	0.989848
851	0.971223	0.991848	1	0.994475	0.989848
852	0.971223	0.991848	1	0.994475	0.989848
853	0.971223	0.991848	1	0.994475	0.989848
854	0.971223	0.991848	1	0.994475	0.989848
855	0.971223	0.991848	1	0.994475	0.989848
856	0.971223	0.991848	1	0.994475	0.989848
857	0.971223	0.991848	1	0.994475	0.989848

858	0.971223	0.991848	1	0.994475	0.989848
859	0.971223	0.991848	1	0.994475	0.989848
860	0.971223	0.991848	1	0.994475	0.989848
861	0.971223	0.991848	1	0.994475	0.989848
862	0.971223	0.991848	1	0.994475	0.989848
863	0.971223	0.991848	1	0.994475	0.989848
864	0.971223	0.991848	1	0.994475	0.989848
865	0.971223	0.991848	1	0.994475	0.989848
866	0.971223	0.991848	1	0.994475	0.989848
867	0.971223	0.991848	1	0.994475	0.989848
868	0.971223	0.991848	1	0.994475	0.989848
869	0.971223	0.991848	1	0.994475	0.989848
870	0.971223	0.991848	1	0.994475	0.989848
871	0.971223	0.991848	1	0.994475	0.989848
872	0.971223	0.991848	1	0.994475	0.989848
873	0.971223	0.991848	1	0.994475	0.989848
874	0.971223	0.991848	1	0.994475	0.989848
875	0.971223	0.991848	1	0.994475	0.989848
876	0.971223	0.991848	1	0.994475	0.989848
877	0.971223	0.991848	1	0.994475	0.989848
878	0.971223	0.991848	1	0.994475	0.989848
879	0.971223	0.991848	1	0.994475	0.989848
880	0.971223	0.991848	1	0.994475	0.989848
881	0.971223	0.991848	1	0.994475	0.989848
882	0.971223	0.991848	1	0.994475	0.989848
883	0.971223	0.991848	1	0.994475	0.989848
884	0.971223	0.991848	1	0.994475	0.989848
885	0.971223	0.991848	1	0.994475	0.989848
886	0.971223	0.991848	1	0.994475	0.989848
887	0.971223	0.991848	1	0.994475	0.989848
888	0.971223	0.991848	1	0.994475	0.989848
889	0.971223	0.991848	1	0.994475	0.989848
890	0.971223	0.991848	1	0.994475	0.989848
891	0.971223	0.991848	1	0.994475	0.989848
892	0.971223	0.991848	1	0.994475	0.989848
893	0.971223	0.991848	1	0.994475	0.989848
894	0.971223	0.991848	1	0.994475	0.989848
895	0.971223	0.991848	1	0.994475	0.989848
896	0.971223	0.991848	1	0.994475	0.989848
897	0.971223	0.991848	1	0.994475	0.989848
898	0.971223	0.991848	1	0.994475	0.989848
899	0.971223	0.991848	1	0.994475	0.989848
900	0.971223	0.991848	1	0.994475	0.989848

901	0.971223	0.991848	1	0.994475	0.989848
902	0.971223	0.991848	1	0.994475	0.989848
903	0.971223	0.991848	1	0.994475	0.989848
904	0.971223	0.991848	1	0.994475	0.989848
905	0.971223	0.991848	1	0.994475	0.989848
906	0.971223	0.991848	1	0.994475	0.989848
907	0.971223	0.991848	1	0.994475	0.989848
908	0.971223	0.991848	1	0.994475	0.989848
909	0.971223	0.991848	1	0.994475	0.989848
910	0.971223	0.991848	1	0.994475	0.989848
911	0.971223	0.991848	1	0.994475	0.989848
912	0.971223	0.991848	1	0.994475	0.989848
913	0.971223	0.991848	1	0.994475	0.989848
914	0.971223	0.991848	1	0.994475	0.989848
915	0.971223	0.991848	1	0.994475	0.989848
916	0.971223	0.991848	1	0.994475	0.989848
917	0.971223	0.991848	1	0.994475	0.989848
918	0.971223	0.991848	1	0.994475	0.989848
919	0.971223	0.991848	1	0.994475	0.989848
920	0.971223	0.991848	1	0.994475	0.989848
921	0.971223	0.991848	1	0.994475	0.989848
922	0.971223	0.991848	1	0.994475	0.989848
923	0.971223	0.991848	1	0.994475	0.989848
924	0.971223	0.991848	1	0.994475	0.989848
925	0.971223	0.991848	1	0.994475	0.989848
926	0.971223	0.991848	1	0.994475	0.989848
927	0.971223	0.991848	1	0.994475	0.989848
928	0.971223	0.991848	1	0.994475	0.989848
929	0.971223	0.991848	1	0.994475	0.989848
930	0.971223	0.991848	1	0.994475	0.989848
931	0.971223	0.991848	1	0.994475	0.989848
932	0.971223	0.991848	1	0.994475	0.989848
933	0.971223	0.991848	1	0.994475	0.989848
934	0.971223	0.991848	1	0.994475	0.989848
935	0.971223	0.991848	1	0.994475	0.989848
936	0.971223	0.991848	1	0.994475	0.989848
937	0.971223	0.991848	1	0.994475	0.989848
938	0.971223	0.991848	1	0.994475	0.989848
939	0.971223	0.991848	1	0.994475	0.989848
940	0.971223	0.991848	1	0.994475	0.989848
941	0.971223	0.991848	1	0.994475	0.989848
942	0.971223	0.991848	1	0.994475	0.989848
943	0.971223	0.991848	1	0.994475	0.989848

944	0.971223	0.991848	1	0.994475	0.989848
945	0.971223	0.991848	1	0.994475	0.989848
946	0.971223	0.991848	1	0.994475	0.989848
947	0.971223	0.991848	1	0.994475	0.989848
948	0.971223	0.991848	1	0.994475	0.989848
949	0.971223	0.991848	1	0.994475	0.989848
950	0.971223	0.991848	1	0.994475	0.989848
951	0.971223	0.991848	1	0.994475	0.989848
952	0.971223	0.991848	1	0.994475	0.989848
953	0.971223	0.991848	1	0.994475	0.989848
954	0.971223	0.991848	1	0.994475	0.989848
955	0.971223	0.991848	1	0.994475	0.989848
956	0.971223	0.991848	1	0.994475	0.989848
957	0.971223	0.991848	1	0.994475	0.989848
958	0.971223	0.991848	1	0.994475	0.989848
959	0.971223	0.991848	1	0.994475	0.989848
960	0.971223	0.991848	1	0.994475	0.989848
961	0.971223	0.991848	1	0.994475	0.989848
962	0.971223	0.991848	1	0.994475	0.989848
963	0.971223	0.991848	1	0.994475	0.989848
964	0.971223	0.991848	1	0.994475	0.989848
965	0.971223	0.991848	1	0.994475	0.989848
966	0.971223	0.991848	1	0.994475	0.989848
967	0.971223	0.991848	1	0.994475	0.989848
968	0.971223	0.991848	1	0.994475	0.989848
969	0.971223	0.991848	1	0.994475	0.989848
970	0.971223	0.991848	1	0.994475	0.989848
971	0.971223	0.991848	1	0.994475	0.989848
972	0.971223	0.991848	1	0.994475	0.989848
973	0.971223	0.991848	1	0.994475	0.989848
974	0.971223	0.991848	1	0.994475	0.989848
975	0.971223	0.991848	1	0.994475	0.989848
976	0.971223	0.991848	1	0.994475	0.989848
977	0.971223	0.991848	1	0.994475	0.989848
978	0.971223	0.991848	1	0.994475	0.989848
979	0.971223	0.991848	1	0.994475	0.989848
980	0.971223	0.991848	1	0.994475	0.989848
981	0.971223	0.991848	1	0.994475	0.989848
982	0.971223	0.991848	1	0.994475	0.989848
983	0.971223	0.991848	1	0.994475	0.989848
984	0.971223	0.991848	1	0.994475	0.989848
985	0.971223	0.991848	1	0.994475	0.989848
986	0.971223	0.991848	1	0.994475	0.989848

987	0.971223	0.991848	1	0.994475	0.989848
988	0.971223	0.991848	1	0.994475	0.989848
989	0.971223	0.991848	1	0.994475	0.989848
990	0.971223	0.991848	1	0.994475	0.989848
991	0.971223	0.991848	1	0.994475	0.989848
992	0.971223	0.991848	1	0.994475	0.989848
993	0.971223	0.991848	1	0.994475	0.989848
994	0.971223	0.991848	1	0.994475	0.989848
995	0.971223	0.991848	1	0.994475	0.989848
996	0.971223	0.991848	1	0.994475	0.989848
997	0.971223	0.991848	1	0.994475	0.989848
998	0.971223	0.991848	1	0.994475	0.989848
999	0.971223	0.991848	1	0.994475	0.989848
1000	0.971223	0.991848	1	0.994475	0.989848

Table S4. The accuracies for different races and total prediction accuracy obtained by IFS method and nearest neighbor algorithm

Number of features	Accuracy for American race	Accuracy for Asian race	Accuracy for European race	Overall prediction accuracy	MCC
4	0.920863	0.845109	0.915789	0.895817	0.8087
5	0.94964	0.817935	0.935526	0.90292	0.820342
6	0.94964	0.850543	0.947368	0.919495	0.851093
7	0.935252	0.855978	0.947368	0.919495	0.850963
8	0.942446	0.883152	0.963158	0.937648	0.884636
9	0.920863	0.888587	0.961842	0.936069	0.881627
10	0.928058	0.899457	0.971053	0.945541	0.899197
11	0.928058	0.902174	0.971053	0.94633	0.900783
12	0.906475	0.9375	0.972368	0.955012	0.916835
13	0.906475	0.934783	0.973684	0.955012	0.916791
14	0.906475	0.945652	0.976316	0.959747	0.92558
15	0.920863	0.945652	0.975	0.960537	0.927109
16	0.928058	0.934783	0.975	0.958169	0.922725
17	0.928058	0.94837	0.977632	0.963694	0.932971
18	0.928058	0.932065	0.977632	0.958958	0.924185
19	0.935252	0.934783	0.977632	0.960537	0.92711
20	0.935252	0.934783	0.977632	0.960537	0.92711
21	0.935252	0.945652	0.977632	0.963694	0.93295
22	0.935252	0.94837	0.976316	0.963694	0.932979
23	0.935252	0.951087	0.984211	0.969219	0.9432
24	0.935252	0.951087	0.986842	0.970797	0.946104
25	0.935252	0.956522	0.989474	0.973954	0.951964
26	0.928058	0.956522	0.986842	0.971586	0.947578
27	0.928058	0.961957	0.988158	0.973954	0.951957
28	0.928058	0.959239	0.986842	0.972376	0.949051
29	0.920863	0.953804	0.985526	0.969219	0.943196
30	0.928058	0.953804	0.989474	0.972376	0.949046
31	0.928058	0.951087	0.989474	0.971586	0.947596
32	0.935252	0.951087	0.990789	0.973165	0.950531
33	0.935252	0.951087	0.990789	0.973165	0.950531
34	0.935252	0.94837	0.986842	0.970008	0.944651
35	0.935252	0.94837	0.986842	0.970008	0.944651
36	0.935252	0.961957	0.986842	0.973954	0.951926
37	0.928058	0.964674	0.984211	0.972376	0.949
38	0.94964	0.967391	0.988158	0.977901	0.959261
39	0.964029	0.967391	0.988158	0.979479	0.962205
40	0.964029	0.972826	0.986842	0.980268	0.963663

41	0.964029	0.972826	0.986842	0.980268	0.963663
42	0.964029	0.972826	0.985526	0.979479	0.962215
43	0.964029	0.975543	0.985526	0.980268	0.963677
44	0.964029	0.975543	0.982895	0.97869	0.960808
45	0.964029	0.972826	0.984211	0.97869	0.960784
46	0.964029	0.972826	0.985526	0.979479	0.962215
47	0.964029	0.972826	0.985526	0.979479	0.962215
48	0.964029	0.970109	0.985526	0.97869	0.960754
49	0.964029	0.970109	0.984211	0.977901	0.959322
50	0.964029	0.967391	0.986842	0.97869	0.960747
51	0.964029	0.967391	0.986842	0.97869	0.960747
52	0.964029	0.970109	0.989474	0.981058	0.965102
53	0.964029	0.964674	0.989474	0.979479	0.962195
54	0.964029	0.964674	0.989474	0.979479	0.962195
55	0.964029	0.970109	0.989474	0.981058	0.965102
56	0.964029	0.967391	0.989474	0.980268	0.963648
57	0.956835	0.970109	0.992105	0.981847	0.966538
58	0.956835	0.972826	0.992105	0.982636	0.967991
59	0.956835	0.975543	0.992105	0.983425	0.969446
60	0.956835	0.972826	0.992105	0.982636	0.967991
61	0.956835	0.975543	0.992105	0.983425	0.969446
62	0.956835	0.975543	0.992105	0.983425	0.969446
63	0.956835	0.978261	0.992105	0.984215	0.970901
64	0.956835	0.978261	0.992105	0.984215	0.970901
65	0.956835	0.970109	0.992105	0.981847	0.966538
66	0.956835	0.970109	0.992105	0.981847	0.966538
67	0.956835	0.970109	0.992105	0.981847	0.966538
68	0.964029	0.980978	0.990789	0.985004	0.972365
69	0.964029	0.980978	0.990789	0.985004	0.972365
70	0.964029	0.978261	0.990789	0.984215	0.970907
71	0.964029	0.978261	0.990789	0.984215	0.970907
72	0.964029	0.978261	0.990789	0.984215	0.970907
73	0.964029	0.980978	0.990789	0.985004	0.972365
74	0.964029	0.980978	0.990789	0.985004	0.972365
75	0.964029	0.978261	0.992105	0.985004	0.972363
76	0.956835	0.978261	0.990789	0.983425	0.969449
77	0.956835	0.978261	0.990789	0.983425	0.969449
78	0.956835	0.978261	0.990789	0.983425	0.969449
79	0.964029	0.978261	0.990789	0.984215	0.970914
80	0.964029	0.980978	0.990789	0.985004	0.972372
81	0.964029	0.975543	0.990789	0.983425	0.969458
82	0.964029	0.978261	0.990789	0.984215	0.970907
83	0.964029	0.970109	0.990789	0.981847	0.966537

84	0.964029	0.972826	0.989474	0.981847	0.966545
85	0.964029	0.970109	0.989474	0.981058	0.965118
86	0.964029	0.970109	0.989474	0.981058	0.965118
87	0.964029	0.972826	0.989474	0.981847	0.966572
88	0.964029	0.972826	0.989474	0.981847	0.966572
89	0.964029	0.972826	0.989474	0.981847	0.966572
90	0.964029	0.970109	0.989474	0.981058	0.965118
91	0.964029	0.972826	0.990789	0.982636	0.968016
92	0.956835	0.972826	0.990789	0.981847	0.966549
93	0.956835	0.972826	0.990789	0.981847	0.966549
94	0.956835	0.970109	0.989474	0.980268	0.963633
95	0.94964	0.970109	0.989474	0.979479	0.962179
96	0.94964	0.970109	0.989474	0.979479	0.962179
97	0.94964	0.970109	0.989474	0.979479	0.962179
98	0.94964	0.972826	0.990789	0.981058	0.965083
99	0.94964	0.975543	0.990789	0.981847	0.966537
100	0.94964	0.975543	0.990789	0.981847	0.966537
101	0.94964	0.975543	0.990789	0.981847	0.966537
102	0.94964	0.975543	0.990789	0.981847	0.966537
103	0.94964	0.972826	0.990789	0.981058	0.965083
104	0.94964	0.972826	0.990789	0.981058	0.965083
105	0.94964	0.972826	0.990789	0.981058	0.965083
106	0.94964	0.972826	0.990789	0.981058	0.965083
107	0.94964	0.972826	0.990789	0.981058	0.965083
108	0.94964	0.972826	0.990789	0.981058	0.965083
109	0.94964	0.972826	0.992105	0.981847	0.96655
110	0.94964	0.972826	0.992105	0.981847	0.96655
111	0.94964	0.970109	0.992105	0.981058	0.965099
112	0.94964	0.970109	0.992105	0.981058	0.965099
113	0.94964	0.972826	0.992105	0.981847	0.96655
114	0.94964	0.970109	0.992105	0.981058	0.965099
115	0.942446	0.972826	0.992105	0.981058	0.965086
116	0.942446	0.970109	0.992105	0.980268	0.963635
117	0.942446	0.970109	0.992105	0.980268	0.963635
118	0.942446	0.970109	0.992105	0.980268	0.963635
119	0.942446	0.970109	0.992105	0.980268	0.963635
120	0.942446	0.970109	0.992105	0.980268	0.963635
121	0.942446	0.970109	0.992105	0.980268	0.963635
122	0.942446	0.970109	0.992105	0.980268	0.963635
123	0.94964	0.970109	0.990789	0.980268	0.963645
124	0.94964	0.970109	0.990789	0.980268	0.963645
125	0.94964	0.970109	0.990789	0.980268	0.963645
126	0.94964	0.970109	0.990789	0.980268	0.963645

127	0.94964	0.970109	0.990789	0.980268	0.963645
128	0.94964	0.970109	0.992105	0.981058	0.965099
129	0.94964	0.970109	0.992105	0.981058	0.965099
130	0.94964	0.970109	0.992105	0.981058	0.965099
131	0.94964	0.970109	0.992105	0.981058	0.965099
132	0.942446	0.975543	0.992105	0.981847	0.96655
133	0.942446	0.972826	0.992105	0.981058	0.965099
134	0.942446	0.972826	0.992105	0.981058	0.965099
135	0.942446	0.967391	0.990789	0.97869	0.960727
136	0.942446	0.967391	0.990789	0.97869	0.960727
137	0.942446	0.975543	0.992105	0.981847	0.96655
138	0.942446	0.975543	0.992105	0.981847	0.96655
139	0.942446	0.972826	0.992105	0.981058	0.965099
140	0.942446	0.972826	0.992105	0.981058	0.965099
141	0.942446	0.972826	0.992105	0.981058	0.965099
142	0.942446	0.972826	0.992105	0.981058	0.965099
143	0.942446	0.970109	0.992105	0.980268	0.96365
144	0.942446	0.970109	0.992105	0.980268	0.96365
145	0.942446	0.970109	0.992105	0.980268	0.96365
146	0.942446	0.970109	0.992105	0.980268	0.96365
147	0.942446	0.970109	0.992105	0.980268	0.96365
148	0.942446	0.972826	0.992105	0.981058	0.965099
149	0.942446	0.972826	0.992105	0.981058	0.965099
150	0.942446	0.972826	0.992105	0.981058	0.965099
151	0.942446	0.972826	0.992105	0.981058	0.965099
152	0.942446	0.972826	0.992105	0.981058	0.965099
153	0.942446	0.970109	0.992105	0.980268	0.96365
154	0.942446	0.972826	0.992105	0.981058	0.965099
155	0.942446	0.972826	0.992105	0.981058	0.965099
156	0.942446	0.972826	0.992105	0.981058	0.965099
157	0.942446	0.972826	0.993421	0.981847	0.966559
158	0.942446	0.972826	0.993421	0.981847	0.966559
159	0.942446	0.972826	0.993421	0.981847	0.966559
160	0.942446	0.972826	0.993421	0.981847	0.966559
161	0.942446	0.972826	0.993421	0.981847	0.966559
162	0.942446	0.972826	0.993421	0.981847	0.966559
163	0.942446	0.970109	0.993421	0.981058	0.96511
164	0.942446	0.970109	0.993421	0.981058	0.96511
165	0.942446	0.970109	0.992105	0.980268	0.96365
166	0.942446	0.970109	0.992105	0.980268	0.96365
167	0.942446	0.970109	0.992105	0.980268	0.96365
168	0.942446	0.972826	0.992105	0.981058	0.965099
169	0.942446	0.972826	0.992105	0.981058	0.965099

170	0.942446	0.972826	0.992105	0.981058	0.965099
171	0.942446	0.972826	0.992105	0.981058	0.965099
172	0.942446	0.972826	0.992105	0.981058	0.965099
173	0.942446	0.970109	0.992105	0.980268	0.96365
174	0.942446	0.970109	0.992105	0.980268	0.96365
175	0.94964	0.970109	0.992105	0.981058	0.965115
176	0.94964	0.970109	0.992105	0.981058	0.965115
177	0.94964	0.970109	0.992105	0.981058	0.965115
178	0.94964	0.970109	0.993421	0.981847	0.966572
179	0.94964	0.970109	0.993421	0.981847	0.966572
180	0.94964	0.970109	0.993421	0.981847	0.966572
181	0.94964	0.970109	0.993421	0.981847	0.966572
182	0.94964	0.970109	0.993421	0.981847	0.966572
183	0.94964	0.972826	0.993421	0.982636	0.968021
184	0.94964	0.975543	0.993421	0.983425	0.96947
185	0.94964	0.972826	0.993421	0.982636	0.968021
186	0.94964	0.972826	0.993421	0.982636	0.968021
187	0.94964	0.975543	0.993421	0.983425	0.96947
188	0.94964	0.975543	0.993421	0.983425	0.96947
189	0.94964	0.975543	0.993421	0.983425	0.96947
190	0.94964	0.975543	0.993421	0.983425	0.96947
191	0.94964	0.975543	0.993421	0.983425	0.96947
192	0.94964	0.975543	0.993421	0.983425	0.96947
193	0.94964	0.975543	0.993421	0.983425	0.96947
194	0.94964	0.972826	0.993421	0.982636	0.968021
195	0.942446	0.972826	0.993421	0.981847	0.966559
196	0.942446	0.972826	0.993421	0.981847	0.966559
197	0.942446	0.972826	0.993421	0.981847	0.966559
198	0.942446	0.972826	0.993421	0.981847	0.966559
199	0.942446	0.972826	0.993421	0.981847	0.966559
200	0.942446	0.972826	0.993421	0.981847	0.966559
201	0.942446	0.972826	0.993421	0.981847	0.966559
202	0.942446	0.972826	0.993421	0.981847	0.966559
203	0.935252	0.972826	0.993421	0.981058	0.965097
204	0.935252	0.972826	0.993421	0.981058	0.965097
205	0.935252	0.972826	0.993421	0.981058	0.965097
206	0.935252	0.975543	0.993421	0.981847	0.966547
207	0.935252	0.975543	0.993421	0.981847	0.966547
208	0.935252	0.975543	0.993421	0.981847	0.966547
209	0.935252	0.975543	0.993421	0.981847	0.966547
210	0.935252	0.972826	0.993421	0.981058	0.965097
211	0.935252	0.972826	0.993421	0.981058	0.965097
212	0.935252	0.972826	0.993421	0.981058	0.965097

213	0.935252	0.972826	0.993421	0.981058	0.965097
214	0.935252	0.975543	0.993421	0.981847	0.966547
215	0.935252	0.975543	0.993421	0.981847	0.966547
216	0.935252	0.972826	0.993421	0.981058	0.965097
217	0.935252	0.972826	0.993421	0.981058	0.965097
218	0.935252	0.972826	0.993421	0.981058	0.965097
219	0.935252	0.972826	0.993421	0.981058	0.965097
220	0.935252	0.975543	0.993421	0.981847	0.966547
221	0.942446	0.975543	0.993421	0.982636	0.968008
222	0.942446	0.975543	0.993421	0.982636	0.968008
223	0.942446	0.975543	0.993421	0.982636	0.968008
224	0.935252	0.975543	0.993421	0.981847	0.966547
225	0.935252	0.975543	0.993421	0.981847	0.966547
226	0.935252	0.975543	0.993421	0.981847	0.966547
227	0.935252	0.975543	0.993421	0.981847	0.966547
228	0.935252	0.972826	0.993421	0.981058	0.965097
229	0.935252	0.972826	0.993421	0.981058	0.965097
230	0.935252	0.972826	0.993421	0.981058	0.965097
231	0.935252	0.972826	0.993421	0.981058	0.965097
232	0.935252	0.972826	0.993421	0.981058	0.965097
233	0.935252	0.972826	0.993421	0.981058	0.965097
234	0.935252	0.970109	0.992105	0.979479	0.962185
235	0.942446	0.970109	0.992105	0.980268	0.96365
236	0.942446	0.970109	0.992105	0.980268	0.96365
237	0.942446	0.970109	0.992105	0.980268	0.96365
238	0.94964	0.972826	0.992105	0.981847	0.966564
239	0.94964	0.972826	0.992105	0.981847	0.966564
240	0.94964	0.970109	0.992105	0.981058	0.965115
241	0.94964	0.970109	0.992105	0.981058	0.965115
242	0.94964	0.970109	0.992105	0.981058	0.965115
243	0.94964	0.967391	0.992105	0.980268	0.963667
244	0.94964	0.967391	0.992105	0.980268	0.963667
245	0.94964	0.967391	0.992105	0.980268	0.963667
246	0.94964	0.970109	0.992105	0.981058	0.965115
247	0.94964	0.970109	0.992105	0.981058	0.965115
248	0.94964	0.970109	0.992105	0.981058	0.965115
249	0.94964	0.970109	0.992105	0.981058	0.965115
250	0.94964	0.970109	0.992105	0.981058	0.965115
251	0.94964	0.970109	0.992105	0.981058	0.965115
252	0.94964	0.970109	0.992105	0.981058	0.965115
253	0.94964	0.970109	0.992105	0.981058	0.965115
254	0.94964	0.970109	0.992105	0.981058	0.965115
255	0.94964	0.972826	0.992105	0.981847	0.966564

256	0.94964	0.970109	0.992105	0.981058	0.965115
257	0.94964	0.972826	0.992105	0.981847	0.966564
258	0.94964	0.972826	0.992105	0.981847	0.966564
259	0.94964	0.972826	0.992105	0.981847	0.966564
260	0.94964	0.972826	0.992105	0.981847	0.966564
261	0.94964	0.972826	0.992105	0.981847	0.966564
262	0.94964	0.970109	0.992105	0.981058	0.965115
263	0.94964	0.970109	0.992105	0.981058	0.965115
264	0.94964	0.970109	0.992105	0.981058	0.965115
265	0.942446	0.970109	0.992105	0.980268	0.96365
266	0.942446	0.970109	0.992105	0.980268	0.96365
267	0.942446	0.970109	0.992105	0.980268	0.96365
268	0.942446	0.967391	0.992105	0.979479	0.962201
269	0.935252	0.967391	0.992105	0.97869	0.960736
270	0.935252	0.967391	0.992105	0.97869	0.960736
271	0.935252	0.967391	0.992105	0.97869	0.960736
272	0.935252	0.967391	0.992105	0.97869	0.960736
273	0.935252	0.967391	0.992105	0.97869	0.960736
274	0.935252	0.967391	0.992105	0.97869	0.960736
275	0.935252	0.970109	0.992105	0.979479	0.962185
276	0.942446	0.972826	0.992105	0.981058	0.965099
277	0.935252	0.972826	0.992105	0.980268	0.963635
278	0.935252	0.970109	0.992105	0.979479	0.962185
279	0.935252	0.970109	0.992105	0.979479	0.962185
280	0.935252	0.970109	0.992105	0.979479	0.962185
281	0.935252	0.970109	0.992105	0.979479	0.962185
282	0.935252	0.970109	0.992105	0.979479	0.962185
283	0.935252	0.970109	0.992105	0.979479	0.962185
284	0.935252	0.970109	0.992105	0.979479	0.962185
285	0.942446	0.970109	0.992105	0.980268	0.96365
286	0.942446	0.970109	0.992105	0.980268	0.96365
287	0.942446	0.964674	0.992105	0.97869	0.960754
288	0.942446	0.964674	0.992105	0.97869	0.960754
289	0.942446	0.964674	0.992105	0.97869	0.960754
290	0.942446	0.964674	0.992105	0.97869	0.960754
291	0.942446	0.961957	0.992105	0.977901	0.959307
292	0.942446	0.961957	0.992105	0.977901	0.959307
293	0.942446	0.961957	0.992105	0.977901	0.959307
294	0.942446	0.961957	0.992105	0.977901	0.959307
295	0.942446	0.959239	0.992105	0.977111	0.957862
296	0.942446	0.959239	0.992105	0.977111	0.957862
297	0.94964	0.959239	0.992105	0.977901	0.959328
298	0.94964	0.959239	0.992105	0.977901	0.959328

299	0.94964	0.961957	0.992105	0.97869	0.960773
300	0.94964	0.961957	0.992105	0.97869	0.960773
301	0.94964	0.964674	0.992105	0.979479	0.962219
302	0.94964	0.964674	0.992105	0.979479	0.962219
303	0.94964	0.967391	0.992105	0.980268	0.963667
304	0.94964	0.967391	0.992105	0.980268	0.963667
305	0.94964	0.967391	0.992105	0.980268	0.963667
306	0.94964	0.967391	0.992105	0.980268	0.963667
307	0.94964	0.967391	0.992105	0.980268	0.963667
308	0.94964	0.967391	0.992105	0.980268	0.963667
309	0.94964	0.967391	0.992105	0.980268	0.963667
310	0.94964	0.967391	0.992105	0.980268	0.963667
311	0.942446	0.967391	0.992105	0.979479	0.962201
312	0.942446	0.967391	0.992105	0.979479	0.962201
313	0.942446	0.967391	0.992105	0.979479	0.962201
314	0.942446	0.964674	0.992105	0.97869	0.960754
315	0.942446	0.964674	0.992105	0.97869	0.960754
316	0.942446	0.964674	0.992105	0.97869	0.960754
317	0.942446	0.967391	0.992105	0.979479	0.962201
318	0.942446	0.967391	0.992105	0.979479	0.962201
319	0.942446	0.964674	0.992105	0.97869	0.960754
320	0.942446	0.964674	0.992105	0.97869	0.960754
321	0.942446	0.961957	0.992105	0.977901	0.959307
322	0.942446	0.961957	0.992105	0.977901	0.959307
323	0.942446	0.961957	0.992105	0.977901	0.959307
324	0.942446	0.961957	0.992105	0.977901	0.959307
325	0.942446	0.961957	0.992105	0.977901	0.959307
326	0.942446	0.964674	0.992105	0.97869	0.960754
327	0.942446	0.964674	0.992105	0.97869	0.960754
328	0.942446	0.961957	0.992105	0.977901	0.959307
329	0.942446	0.959239	0.992105	0.977111	0.957862
330	0.942446	0.961957	0.992105	0.977901	0.959307
331	0.942446	0.961957	0.992105	0.977901	0.959307
332	0.942446	0.961957	0.992105	0.977901	0.959307
333	0.935252	0.961957	0.993421	0.977901	0.959308
334	0.935252	0.964674	0.993421	0.97869	0.960754
335	0.942446	0.964674	0.993421	0.979479	0.962216
336	0.942446	0.972826	0.993421	0.981847	0.966559
337	0.942446	0.975543	0.993421	0.982636	0.968008
338	0.942446	0.972826	0.993421	0.981847	0.966559
339	0.942446	0.972826	0.993421	0.981847	0.966559
340	0.94964	0.972826	0.993421	0.982636	0.968021
341	0.94964	0.972826	0.993421	0.982636	0.968021

342	0.94964	0.970109	0.993421	0.981847	0.966572
343	0.94964	0.970109	0.993421	0.981847	0.966572
344	0.94964	0.970109	0.993421	0.981847	0.966572
345	0.94964	0.970109	0.993421	0.981847	0.966572
346	0.94964	0.970109	0.993421	0.981847	0.966572
347	0.94964	0.970109	0.993421	0.981847	0.966572
348	0.94964	0.970109	0.993421	0.981847	0.966572
349	0.94964	0.970109	0.993421	0.981847	0.966572
350	0.94964	0.964674	0.993421	0.980268	0.963679
351	0.94964	0.964674	0.993421	0.980268	0.963679
352	0.94964	0.964674	0.993421	0.980268	0.963679
353	0.94964	0.964674	0.993421	0.980268	0.963679
354	0.94964	0.964674	0.993421	0.980268	0.963679
355	0.94964	0.964674	0.993421	0.980268	0.963679
356	0.94964	0.964674	0.993421	0.980268	0.963679
357	0.94964	0.961957	0.993421	0.979479	0.962234
358	0.942446	0.964674	0.993421	0.979479	0.962216
359	0.942446	0.967391	0.993421	0.980268	0.963663
360	0.942446	0.964674	0.993421	0.979479	0.962216
361	0.942446	0.964674	0.993421	0.979479	0.962216
362	0.942446	0.961957	0.993421	0.97869	0.960771
363	0.942446	0.961957	0.993421	0.97869	0.960771
364	0.942446	0.961957	0.993421	0.97869	0.960771
365	0.942446	0.961957	0.993421	0.97869	0.960771
366	0.942446	0.961957	0.993421	0.97869	0.960771
367	0.942446	0.961957	0.993421	0.97869	0.960771
368	0.942446	0.961957	0.993421	0.97869	0.960771
369	0.942446	0.961957	0.993421	0.97869	0.960771
370	0.942446	0.961957	0.993421	0.97869	0.960771
371	0.942446	0.961957	0.993421	0.97869	0.960771
372	0.942446	0.961957	0.993421	0.97869	0.960771
373	0.942446	0.961957	0.993421	0.97869	0.960771
374	0.942446	0.961957	0.993421	0.97869	0.960771
375	0.94964	0.961957	0.993421	0.979479	0.962234
376	0.94964	0.961957	0.993421	0.979479	0.962234
377	0.94964	0.961957	0.993421	0.979479	0.962234
378	0.94964	0.961957	0.993421	0.979479	0.962234
379	0.94964	0.961957	0.993421	0.979479	0.962234
380	0.94964	0.961957	0.993421	0.979479	0.962234
381	0.94964	0.961957	0.993421	0.979479	0.962234
382	0.942446	0.967391	0.993421	0.980268	0.963663
383	0.942446	0.967391	0.993421	0.980268	0.963663
384	0.942446	0.967391	0.993421	0.980268	0.963648

385	0.942446	0.961957	0.993421	0.97869	0.960754
386	0.935252	0.967391	0.993421	0.979479	0.962187
387	0.935252	0.967391	0.993421	0.979479	0.962187
388	0.935252	0.967391	0.993421	0.979479	0.962187
389	0.935252	0.967391	0.993421	0.979479	0.962187
390	0.935252	0.959239	0.993421	0.977111	0.957846
391	0.935252	0.959239	0.993421	0.977111	0.957846
392	0.935252	0.961957	0.993421	0.977901	0.959292
393	0.935252	0.961957	0.993421	0.977901	0.959292
394	0.935252	0.961957	0.993421	0.977901	0.959292
395	0.935252	0.961957	0.993421	0.977901	0.959292
396	0.935252	0.961957	0.993421	0.977901	0.959292
397	0.935252	0.961957	0.993421	0.977901	0.959292
398	0.935252	0.961957	0.993421	0.977901	0.959292
399	0.942446	0.961957	0.993421	0.97869	0.960754
400	0.942446	0.961957	0.993421	0.97869	0.960754
401	0.94964	0.961957	0.993421	0.979479	0.962216
402	0.94964	0.961957	0.993421	0.979479	0.962216
403	0.94964	0.961957	0.993421	0.979479	0.962216
404	0.94964	0.961957	0.993421	0.979479	0.962216
405	0.94964	0.964674	0.993421	0.980268	0.963663
406	0.94964	0.961957	0.993421	0.979479	0.962216
407	0.94964	0.961957	0.993421	0.979479	0.962216
408	0.94964	0.961957	0.993421	0.979479	0.962216
409	0.94964	0.961957	0.993421	0.979479	0.962216
410	0.956835	0.961957	0.993421	0.980268	0.963679
411	0.956835	0.959239	0.993421	0.979479	0.962234
412	0.94964	0.959239	0.993421	0.97869	0.960771
413	0.94964	0.961957	0.993421	0.979479	0.962216
414	0.94964	0.961957	0.993421	0.979479	0.962216
415	0.94964	0.961957	0.993421	0.979479	0.962216
416	0.94964	0.959239	0.993421	0.97869	0.960771
417	0.94964	0.959239	0.993421	0.97869	0.960771
418	0.94964	0.959239	0.993421	0.97869	0.960771
419	0.94964	0.959239	0.993421	0.97869	0.960771
420	0.94964	0.959239	0.993421	0.97869	0.960771
421	0.94964	0.959239	0.993421	0.97869	0.960771
422	0.94964	0.956522	0.993421	0.977901	0.959327
423	0.94964	0.956522	0.993421	0.977901	0.959327
424	0.94964	0.953804	0.993421	0.977111	0.957884
425	0.94964	0.953804	0.993421	0.977111	0.957884
426	0.942446	0.953804	0.993421	0.976322	0.95642
427	0.94964	0.959239	0.993421	0.97869	0.960771

428	0.942446	0.959239	0.993421	0.977901	0.959308
429	0.942446	0.959239	0.993421	0.977901	0.959308
430	0.942446	0.959239	0.993421	0.977901	0.959308
431	0.94964	0.959239	0.993421	0.97869	0.960771
432	0.94964	0.959239	0.993421	0.97869	0.960771
433	0.942446	0.959239	0.993421	0.977901	0.959308
434	0.942446	0.959239	0.993421	0.977901	0.959308
435	0.942446	0.959239	0.993421	0.977901	0.959308
436	0.942446	0.959239	0.993421	0.977901	0.959308
437	0.942446	0.959239	0.993421	0.977901	0.959308
438	0.942446	0.959239	0.993421	0.977901	0.959308
439	0.942446	0.959239	0.993421	0.977901	0.959308
440	0.942446	0.959239	0.993421	0.977901	0.959308
441	0.942446	0.956522	0.993421	0.977111	0.957864
442	0.942446	0.956522	0.993421	0.977111	0.957864
443	0.942446	0.956522	0.993421	0.977111	0.957864
444	0.942446	0.956522	0.993421	0.977111	0.957864
445	0.94964	0.956522	0.993421	0.977901	0.959327
446	0.94964	0.956522	0.993421	0.977901	0.959327
447	0.94964	0.956522	0.993421	0.977901	0.959327
448	0.94964	0.956522	0.993421	0.977901	0.959327
449	0.942446	0.956522	0.993421	0.977111	0.957864
450	0.942446	0.956522	0.993421	0.977111	0.957864
451	0.942446	0.956522	0.993421	0.977111	0.957864
452	0.942446	0.956522	0.993421	0.977111	0.957864
453	0.942446	0.956522	0.993421	0.977111	0.957864
454	0.935252	0.956522	0.994737	0.977111	0.957877
455	0.935252	0.956522	0.994737	0.977111	0.957877
456	0.935252	0.956522	0.994737	0.977111	0.957877
457	0.935252	0.956522	0.994737	0.977111	0.957877
458	0.935252	0.956522	0.994737	0.977111	0.957877
459	0.935252	0.956522	0.994737	0.977111	0.957877
460	0.935252	0.953804	0.994737	0.976322	0.956434
461	0.935252	0.953804	0.994737	0.976322	0.956434
462	0.935252	0.953804	0.994737	0.976322	0.956434
463	0.935252	0.953804	0.994737	0.976322	0.956434
464	0.935252	0.953804	0.994737	0.976322	0.956434
465	0.935252	0.953804	0.994737	0.976322	0.956434
466	0.935252	0.953804	0.994737	0.976322	0.956434
467	0.935252	0.953804	0.994737	0.976322	0.956434
468	0.935252	0.953804	0.994737	0.976322	0.956434
469	0.94964	0.953804	0.994737	0.977901	0.959354
470	0.94964	0.953804	0.994737	0.977901	0.959354

471	0.94964	0.956522	0.994737	0.97869	0.960797
472	0.94964	0.956522	0.994737	0.97869	0.960797
473	0.94964	0.956522	0.994737	0.97869	0.960797
474	0.94964	0.956522	0.994737	0.97869	0.960797
475	0.942446	0.956522	0.994737	0.977901	0.959336
476	0.942446	0.956522	0.994737	0.977901	0.959336
477	0.935252	0.956522	0.994737	0.977111	0.957877
478	0.942446	0.956522	0.994737	0.977901	0.959336
479	0.942446	0.956522	0.994737	0.977901	0.959336
480	0.942446	0.956522	0.994737	0.977901	0.959336
481	0.942446	0.959239	0.994737	0.97869	0.96078
482	0.942446	0.959239	0.994737	0.97869	0.96078
483	0.935252	0.956522	0.994737	0.977111	0.957877
484	0.935252	0.959239	0.994737	0.977901	0.959321
485	0.935252	0.959239	0.994737	0.977901	0.959321
486	0.935252	0.956522	0.994737	0.977111	0.957877
487	0.935252	0.959239	0.994737	0.977901	0.959321
488	0.935252	0.959239	0.994737	0.977901	0.959321
489	0.935252	0.956522	0.994737	0.977111	0.957877
490	0.935252	0.956522	0.994737	0.977111	0.957877
491	0.935252	0.956522	0.994737	0.977111	0.957877
492	0.935252	0.956522	0.994737	0.977111	0.957877
493	0.935252	0.953804	0.994737	0.976322	0.956434
494	0.935252	0.953804	0.994737	0.976322	0.956434
495	0.935252	0.953804	0.994737	0.976322	0.956434
496	0.935252	0.953804	0.994737	0.976322	0.956434
497	0.935252	0.953804	0.994737	0.976322	0.956434
498	0.935252	0.953804	0.994737	0.976322	0.956434
499	0.935252	0.953804	0.994737	0.976322	0.956434
500	0.935252	0.953804	0.994737	0.976322	0.956434
501	0.935252	0.956522	0.994737	0.977111	0.957877
502	0.935252	0.956522	0.994737	0.977111	0.957877
503	0.935252	0.956522	0.994737	0.977111	0.957877
504	0.935252	0.956522	0.994737	0.977111	0.957877
505	0.935252	0.956522	0.994737	0.977111	0.957877
506	0.942446	0.956522	0.994737	0.977901	0.959336
507	0.935252	0.956522	0.994737	0.977111	0.957877
508	0.935252	0.956522	0.994737	0.977111	0.957877
509	0.935252	0.956522	0.994737	0.977111	0.957877
510	0.935252	0.956522	0.994737	0.977111	0.957877
511	0.935252	0.956522	0.994737	0.977111	0.957877
512	0.935252	0.953804	0.994737	0.976322	0.956434
513	0.935252	0.953804	0.994737	0.976322	0.956434

514	0.935252	0.953804	0.994737	0.976322	0.956434
515	0.935252	0.953804	0.994737	0.976322	0.956434
516	0.935252	0.953804	0.994737	0.976322	0.956434
517	0.935252	0.951087	0.994737	0.975533	0.954992
518	0.935252	0.94837	0.994737	0.974743	0.953552
519	0.935252	0.94837	0.994737	0.974743	0.953552
520	0.935252	0.94837	0.994737	0.974743	0.953552
521	0.935252	0.94837	0.994737	0.974743	0.953552
522	0.935252	0.94837	0.994737	0.974743	0.953552
523	0.935252	0.94837	0.994737	0.974743	0.953552
524	0.935252	0.94837	0.994737	0.974743	0.953552
525	0.935252	0.94837	0.994737	0.974743	0.953552
526	0.935252	0.94837	0.994737	0.974743	0.953552
527	0.935252	0.94837	0.994737	0.974743	0.953552
528	0.935252	0.94837	0.994737	0.974743	0.953552
529	0.935252	0.951087	0.994737	0.975533	0.954992
530	0.935252	0.951087	0.994737	0.975533	0.954992
531	0.935252	0.951087	0.994737	0.975533	0.954992
532	0.935252	0.951087	0.994737	0.975533	0.954992
533	0.935252	0.951087	0.994737	0.975533	0.954992
534	0.935252	0.951087	0.994737	0.975533	0.954992
535	0.935252	0.951087	0.994737	0.975533	0.954992
536	0.935252	0.951087	0.994737	0.975533	0.954992
537	0.935252	0.951087	0.994737	0.975533	0.954992
538	0.935252	0.953804	0.994737	0.976322	0.956434
539	0.935252	0.953804	0.994737	0.976322	0.956434
540	0.935252	0.953804	0.994737	0.976322	0.956434
541	0.935252	0.956522	0.994737	0.977111	0.957877
542	0.935252	0.956522	0.994737	0.977111	0.957877
543	0.935252	0.953804	0.994737	0.976322	0.956434
544	0.935252	0.956522	0.994737	0.977111	0.957877
545	0.935252	0.956522	0.994737	0.977111	0.957877
546	0.935252	0.956522	0.994737	0.977111	0.957877
547	0.935252	0.959239	0.994737	0.977901	0.959321
548	0.935252	0.959239	0.994737	0.977901	0.959321
549	0.935252	0.959239	0.994737	0.977901	0.959321
550	0.935252	0.959239	0.994737	0.977901	0.959321
551	0.935252	0.959239	0.994737	0.977901	0.959321
552	0.935252	0.959239	0.994737	0.977901	0.959321
553	0.935252	0.959239	0.994737	0.977901	0.959321
554	0.935252	0.959239	0.994737	0.977901	0.959321
555	0.935252	0.959239	0.994737	0.977901	0.959321
556	0.935252	0.959239	0.994737	0.977901	0.959321

557	0.935252	0.959239	0.994737	0.977901	0.959321
558	0.935252	0.959239	0.994737	0.977901	0.959321
559	0.935252	0.961957	0.994737	0.97869	0.960765
560	0.935252	0.953804	0.994737	0.976322	0.956434
561	0.935252	0.953804	0.994737	0.976322	0.956434
562	0.935252	0.953804	0.994737	0.976322	0.956434
563	0.935252	0.956522	0.994737	0.977111	0.957877
564	0.935252	0.956522	0.994737	0.977111	0.957877
565	0.935252	0.956522	0.994737	0.977111	0.957877
566	0.935252	0.956522	0.994737	0.977111	0.957877
567	0.935252	0.956522	0.994737	0.977111	0.957877
568	0.935252	0.959239	0.994737	0.977901	0.959321
569	0.935252	0.959239	0.994737	0.977901	0.959321
570	0.935252	0.959239	0.994737	0.977901	0.959321
571	0.935252	0.959239	0.994737	0.977901	0.959321
572	0.935252	0.959239	0.994737	0.977901	0.959321
573	0.935252	0.959239	0.994737	0.977901	0.959321
574	0.935252	0.959239	0.994737	0.977901	0.959321
575	0.935252	0.959239	0.994737	0.977901	0.959321
576	0.935252	0.959239	0.994737	0.977901	0.959321
577	0.935252	0.959239	0.994737	0.977901	0.959321
578	0.935252	0.959239	0.994737	0.977901	0.959321
579	0.935252	0.959239	0.994737	0.977901	0.959321
580	0.935252	0.959239	0.994737	0.977901	0.959321
581	0.935252	0.959239	0.994737	0.977901	0.959321
582	0.935252	0.959239	0.994737	0.977901	0.959321
583	0.935252	0.959239	0.994737	0.977901	0.959321
584	0.935252	0.959239	0.994737	0.977901	0.959321
585	0.935252	0.959239	0.994737	0.977901	0.959321
586	0.935252	0.956522	0.994737	0.977111	0.957877
587	0.935252	0.956522	0.994737	0.977111	0.957877
588	0.935252	0.956522	0.994737	0.977111	0.957877
589	0.935252	0.956522	0.994737	0.977111	0.957877
590	0.942446	0.956522	0.994737	0.977901	0.959336
591	0.942446	0.956522	0.994737	0.977901	0.959336
592	0.942446	0.959239	0.994737	0.97869	0.96078
593	0.942446	0.959239	0.994737	0.97869	0.96078
594	0.942446	0.959239	0.994737	0.97869	0.96078
595	0.942446	0.959239	0.994737	0.97869	0.96078
596	0.942446	0.959239	0.994737	0.97869	0.96078
597	0.942446	0.959239	0.994737	0.97869	0.96078
598	0.942446	0.959239	0.994737	0.97869	0.96078
599	0.942446	0.959239	0.994737	0.97869	0.96078

600	0.942446	0.959239	0.994737	0.97869	0.96078
601	0.942446	0.959239	0.994737	0.97869	0.96078
602	0.942446	0.959239	0.994737	0.97869	0.96078
603	0.942446	0.959239	0.994737	0.97869	0.96078
604	0.942446	0.959239	0.994737	0.97869	0.96078
605	0.942446	0.959239	0.994737	0.97869	0.96078
606	0.942446	0.959239	0.994737	0.97869	0.96078
607	0.942446	0.959239	0.994737	0.97869	0.96078
608	0.942446	0.953804	0.994737	0.977111	0.957894
609	0.942446	0.953804	0.994737	0.977111	0.957894
610	0.942446	0.953804	0.994737	0.977111	0.957894
611	0.942446	0.953804	0.994737	0.977111	0.957894
612	0.942446	0.953804	0.994737	0.977111	0.957894
613	0.942446	0.953804	0.994737	0.977111	0.957894
614	0.942446	0.953804	0.994737	0.977111	0.957894
615	0.942446	0.953804	0.994737	0.977111	0.957894
616	0.942446	0.951087	0.994737	0.976322	0.956453
617	0.942446	0.951087	0.994737	0.976322	0.956453
618	0.942446	0.951087	0.994737	0.976322	0.956453
619	0.942446	0.94837	0.994737	0.975533	0.955012
620	0.942446	0.94837	0.994737	0.975533	0.955012
621	0.942446	0.94837	0.994737	0.975533	0.955012
622	0.942446	0.94837	0.994737	0.975533	0.955012
623	0.942446	0.94837	0.994737	0.975533	0.955012
624	0.942446	0.94837	0.994737	0.975533	0.955012
625	0.942446	0.94837	0.994737	0.975533	0.955012
626	0.942446	0.94837	0.994737	0.975533	0.955012
627	0.942446	0.94837	0.994737	0.975533	0.955012
628	0.942446	0.94837	0.994737	0.975533	0.955012
629	0.942446	0.94837	0.994737	0.975533	0.955012
630	0.942446	0.94837	0.994737	0.975533	0.955012
631	0.942446	0.94837	0.994737	0.975533	0.955012
632	0.942446	0.94837	0.994737	0.975533	0.955012
633	0.942446	0.945652	0.994737	0.974743	0.953573
634	0.942446	0.945652	0.994737	0.974743	0.953573
635	0.942446	0.945652	0.994737	0.974743	0.953573
636	0.942446	0.945652	0.994737	0.974743	0.953573
637	0.942446	0.945652	0.994737	0.974743	0.953573
638	0.942446	0.945652	0.994737	0.974743	0.953573
639	0.942446	0.945652	0.994737	0.974743	0.953573
640	0.942446	0.945652	0.994737	0.974743	0.953573
641	0.942446	0.945652	0.994737	0.974743	0.953573
642	0.942446	0.942935	0.994737	0.973954	0.952135

643	0.942446	0.942935	0.994737	0.973954	0.952135
644	0.942446	0.942935	0.994737	0.973954	0.952135
645	0.942446	0.942935	0.994737	0.973954	0.952135
646	0.942446	0.942935	0.994737	0.973954	0.952135
647	0.942446	0.942935	0.994737	0.973954	0.952135
648	0.942446	0.942935	0.994737	0.973954	0.952135
649	0.942446	0.942935	0.994737	0.973954	0.952135
650	0.942446	0.945652	0.994737	0.974743	0.953573
651	0.942446	0.94837	0.994737	0.975533	0.955012
652	0.942446	0.951087	0.994737	0.976322	0.956453
653	0.942446	0.951087	0.994737	0.976322	0.956453
654	0.942446	0.94837	0.994737	0.975533	0.955012
655	0.942446	0.94837	0.994737	0.975533	0.955012
656	0.942446	0.945652	0.994737	0.974743	0.953573
657	0.942446	0.945652	0.994737	0.974743	0.953573
658	0.942446	0.942935	0.994737	0.973954	0.952135
659	0.942446	0.945652	0.993421	0.973954	0.952073
660	0.942446	0.945652	0.993421	0.973954	0.952073
661	0.942446	0.945652	0.993421	0.973954	0.952073
662	0.942446	0.945652	0.993421	0.973954	0.952073
663	0.942446	0.945652	0.993421	0.973954	0.952073
664	0.942446	0.945652	0.993421	0.973954	0.952073
665	0.942446	0.945652	0.993421	0.973954	0.952073
666	0.942446	0.945652	0.993421	0.973954	0.952073
667	0.942446	0.945652	0.993421	0.973954	0.952073
668	0.942446	0.940217	0.993421	0.972376	0.949192
669	0.942446	0.940217	0.993421	0.972376	0.949192
670	0.942446	0.940217	0.993421	0.972376	0.949192
671	0.942446	0.940217	0.993421	0.972376	0.949192
672	0.942446	0.940217	0.993421	0.972376	0.949192
673	0.942446	0.940217	0.993421	0.972376	0.949192
674	0.942446	0.940217	0.993421	0.972376	0.949192
675	0.942446	0.940217	0.993421	0.972376	0.949192
676	0.942446	0.940217	0.993421	0.972376	0.949192
677	0.942446	0.940217	0.993421	0.972376	0.949192
678	0.942446	0.940217	0.993421	0.972376	0.949192
679	0.942446	0.940217	0.993421	0.972376	0.949192
680	0.942446	0.940217	0.993421	0.972376	0.949192
681	0.942446	0.942935	0.993421	0.973165	0.950632
682	0.942446	0.942935	0.993421	0.973165	0.950632
683	0.942446	0.942935	0.993421	0.973165	0.950632
684	0.942446	0.94837	0.993421	0.974743	0.953515
685	0.942446	0.945652	0.993421	0.973954	0.952073

686	0.942446	0.942935	0.993421	0.973165	0.950632
687	0.942446	0.945652	0.993421	0.973954	0.952073
688	0.942446	0.94837	0.993421	0.974743	0.953515
689	0.942446	0.94837	0.993421	0.974743	0.953515
690	0.942446	0.94837	0.993421	0.974743	0.953515
691	0.942446	0.94837	0.993421	0.974743	0.953515
692	0.942446	0.94837	0.993421	0.974743	0.953515
693	0.942446	0.945652	0.993421	0.973954	0.952073
694	0.942446	0.945652	0.993421	0.973954	0.952073
695	0.942446	0.942935	0.993421	0.973165	0.950632
696	0.942446	0.942935	0.993421	0.973165	0.950632
697	0.942446	0.945652	0.993421	0.973954	0.952073
698	0.942446	0.945652	0.993421	0.973954	0.952073
699	0.942446	0.945652	0.993421	0.973954	0.952073
700	0.942446	0.945652	0.993421	0.973954	0.952073
701	0.942446	0.94837	0.993421	0.974743	0.953515
702	0.942446	0.945652	0.993421	0.973954	0.952073
703	0.942446	0.945652	0.993421	0.973954	0.952073
704	0.942446	0.945652	0.993421	0.973954	0.952073
705	0.942446	0.945652	0.993421	0.973954	0.952073
706	0.942446	0.945652	0.993421	0.973954	0.952073
707	0.942446	0.945652	0.994737	0.974743	0.953552
708	0.942446	0.945652	0.994737	0.974743	0.953552
709	0.942446	0.945652	0.994737	0.974743	0.953552
710	0.942446	0.942935	0.994737	0.973954	0.952112
711	0.942446	0.942935	0.994737	0.973954	0.952112
712	0.942446	0.942935	0.994737	0.973954	0.952112
713	0.942446	0.942935	0.994737	0.973954	0.952112
714	0.942446	0.942935	0.994737	0.973954	0.952112
715	0.942446	0.942935	0.994737	0.973954	0.952112
716	0.942446	0.9375	0.994737	0.972376	0.949236
717	0.942446	0.9375	0.994737	0.972376	0.949236
718	0.942446	0.9375	0.994737	0.972376	0.949236
719	0.942446	0.9375	0.994737	0.972376	0.949236
720	0.942446	0.9375	0.994737	0.972376	0.949236
721	0.942446	0.9375	0.994737	0.972376	0.949236
722	0.942446	0.9375	0.994737	0.972376	0.949236
723	0.942446	0.9375	0.994737	0.972376	0.949236
724	0.942446	0.9375	0.994737	0.972376	0.949236
725	0.942446	0.9375	0.994737	0.972376	0.949236
726	0.942446	0.9375	0.994737	0.972376	0.949236
727	0.942446	0.9375	0.994737	0.972376	0.949236
728	0.942446	0.9375	0.994737	0.972376	0.949236

729	0.942446	0.9375	0.994737	0.972376	0.949236
730	0.942446	0.9375	0.994737	0.972376	0.949236
731	0.942446	0.9375	0.994737	0.972376	0.949236
732	0.942446	0.9375	0.994737	0.972376	0.949236
733	0.942446	0.940217	0.994737	0.973165	0.950673
734	0.942446	0.940217	0.994737	0.973165	0.950673
735	0.942446	0.940217	0.994737	0.973165	0.950673
736	0.942446	0.940217	0.994737	0.973165	0.950673
737	0.942446	0.940217	0.994737	0.973165	0.950673
738	0.942446	0.940217	0.994737	0.973165	0.950673
739	0.942446	0.942935	0.994737	0.973954	0.952112
740	0.942446	0.942935	0.994737	0.973954	0.952112
741	0.942446	0.942935	0.994737	0.973954	0.952112
742	0.942446	0.940217	0.994737	0.973165	0.950673
743	0.942446	0.9375	0.994737	0.972376	0.949236
744	0.942446	0.9375	0.994737	0.972376	0.949236
745	0.942446	0.9375	0.994737	0.972376	0.949236
746	0.942446	0.934783	0.994737	0.971586	0.947799
747	0.942446	0.934783	0.994737	0.971586	0.947799
748	0.942446	0.934783	0.994737	0.971586	0.947799
749	0.942446	0.934783	0.993421	0.970797	0.946316
750	0.942446	0.934783	0.993421	0.970797	0.946316
751	0.942446	0.934783	0.993421	0.970797	0.946316
752	0.942446	0.934783	0.993421	0.970797	0.946316
753	0.942446	0.934783	0.993421	0.970797	0.946316
754	0.942446	0.934783	0.993421	0.970797	0.946316
755	0.942446	0.934783	0.993421	0.970797	0.946316
756	0.942446	0.932065	0.993421	0.970008	0.944879
757	0.942446	0.932065	0.993421	0.970008	0.944879
758	0.942446	0.932065	0.994737	0.970797	0.946363
759	0.942446	0.932065	0.994737	0.970797	0.946363
760	0.942446	0.932065	0.994737	0.970797	0.946363
761	0.942446	0.932065	0.994737	0.970797	0.946363
762	0.942446	0.932065	0.994737	0.970797	0.946363
763	0.942446	0.932065	0.994737	0.970797	0.946363
764	0.942446	0.934783	0.994737	0.971586	0.947799
765	0.942446	0.934783	0.994737	0.971586	0.947799
766	0.942446	0.934783	0.994737	0.971586	0.947799
767	0.942446	0.934783	0.994737	0.971586	0.947799
768	0.942446	0.934783	0.994737	0.971586	0.947799
769	0.942446	0.9375	0.994737	0.972376	0.949236
770	0.942446	0.9375	0.994737	0.972376	0.949236
771	0.942446	0.9375	0.994737	0.972376	0.949236

772	0.942446	0.9375	0.994737	0.972376	0.949236
773	0.942446	0.9375	0.994737	0.972376	0.949236
774	0.942446	0.934783	0.994737	0.971586	0.947799
775	0.942446	0.934783	0.994737	0.971586	0.947799
776	0.942446	0.934783	0.994737	0.971586	0.947799
777	0.942446	0.934783	0.994737	0.971586	0.947799
778	0.942446	0.932065	0.994737	0.970797	0.946363
779	0.942446	0.932065	0.994737	0.970797	0.946363
780	0.942446	0.934783	0.994737	0.971586	0.947799
781	0.942446	0.934783	0.994737	0.971586	0.947799
782	0.942446	0.934783	0.994737	0.971586	0.947799
783	0.942446	0.934783	0.994737	0.971586	0.947799
784	0.942446	0.934783	0.994737	0.971586	0.947799
785	0.942446	0.934783	0.994737	0.971586	0.947799
786	0.942446	0.934783	0.994737	0.971586	0.947799
787	0.942446	0.934783	0.994737	0.971586	0.947799
788	0.942446	0.934783	0.994737	0.971586	0.947799
789	0.942446	0.934783	0.994737	0.971586	0.947799
790	0.942446	0.932065	0.994737	0.970797	0.946337
791	0.942446	0.932065	0.994737	0.970797	0.946337
792	0.942446	0.932065	0.994737	0.970797	0.946337
793	0.942446	0.932065	0.994737	0.970797	0.946337
794	0.942446	0.932065	0.994737	0.970797	0.946337
795	0.942446	0.932065	0.994737	0.970797	0.946337
796	0.942446	0.932065	0.994737	0.970797	0.946337
797	0.942446	0.932065	0.994737	0.970797	0.946337
798	0.942446	0.932065	0.994737	0.970797	0.946337
799	0.942446	0.932065	0.994737	0.970797	0.946337
800	0.942446	0.932065	0.994737	0.970797	0.946337
801	0.942446	0.932065	0.994737	0.970797	0.946337
802	0.942446	0.932065	0.994737	0.970797	0.946337
803	0.942446	0.932065	0.994737	0.970797	0.946337
804	0.942446	0.932065	0.994737	0.970797	0.946337
805	0.942446	0.932065	0.994737	0.970797	0.946337
806	0.942446	0.932065	0.994737	0.970797	0.946337
807	0.942446	0.932065	0.994737	0.970797	0.946337
808	0.942446	0.932065	0.994737	0.970797	0.946337
809	0.942446	0.934783	0.994737	0.971586	0.947774
810	0.942446	0.934783	0.994737	0.971586	0.947774
811	0.942446	0.934783	0.994737	0.971586	0.947774
812	0.942446	0.934783	0.994737	0.971586	0.947774
813	0.942446	0.932065	0.994737	0.970797	0.946337
814	0.942446	0.932065	0.994737	0.970797	0.946337

815	0.942446	0.932065	0.994737	0.970797	0.946337
816	0.942446	0.932065	0.994737	0.970797	0.946337
817	0.942446	0.932065	0.994737	0.970797	0.946337
818	0.942446	0.932065	0.994737	0.970797	0.946337
819	0.942446	0.932065	0.994737	0.970797	0.946337
820	0.942446	0.932065	0.994737	0.970797	0.946337
821	0.942446	0.932065	0.994737	0.970797	0.946337
822	0.942446	0.932065	0.994737	0.970797	0.946337
823	0.942446	0.932065	0.994737	0.970797	0.946337
824	0.942446	0.932065	0.994737	0.970797	0.946337
825	0.942446	0.932065	0.994737	0.970797	0.946337
826	0.942446	0.932065	0.994737	0.970797	0.946337
827	0.942446	0.932065	0.994737	0.970797	0.946337
828	0.942446	0.929348	0.994737	0.970008	0.944901
829	0.942446	0.929348	0.994737	0.970008	0.944901
830	0.942446	0.929348	0.994737	0.970008	0.944901
831	0.942446	0.929348	0.994737	0.970008	0.944901
832	0.942446	0.929348	0.994737	0.970008	0.944901
833	0.942446	0.929348	0.994737	0.970008	0.944901
834	0.942446	0.929348	0.994737	0.970008	0.944901
835	0.942446	0.929348	0.994737	0.970008	0.944901
836	0.942446	0.92663	0.994737	0.969219	0.943466
837	0.942446	0.92663	0.994737	0.969219	0.943466
838	0.942446	0.92663	0.994737	0.969219	0.943466
839	0.942446	0.92663	0.994737	0.969219	0.943466
840	0.942446	0.92663	0.994737	0.969219	0.943466
841	0.942446	0.92663	0.994737	0.969219	0.943466
842	0.942446	0.92663	0.994737	0.969219	0.943466
843	0.942446	0.92663	0.994737	0.969219	0.943466
844	0.942446	0.92663	0.994737	0.969219	0.943466
845	0.942446	0.92663	0.994737	0.969219	0.943466
846	0.942446	0.92663	0.994737	0.969219	0.943466
847	0.942446	0.923913	0.994737	0.968429	0.942032
848	0.942446	0.923913	0.994737	0.968429	0.942032
849	0.942446	0.923913	0.994737	0.968429	0.942032
850	0.942446	0.923913	0.994737	0.968429	0.942032
851	0.942446	0.923913	0.994737	0.968429	0.942032
852	0.942446	0.921196	0.994737	0.96764	0.940599
853	0.942446	0.921196	0.994737	0.96764	0.940599
854	0.942446	0.921196	0.994737	0.96764	0.940599
855	0.942446	0.921196	0.994737	0.96764	0.940599
856	0.942446	0.921196	0.994737	0.96764	0.940599
857	0.942446	0.921196	0.994737	0.96764	0.940599

858	0.942446	0.923913	0.994737	0.968429	0.942032
859	0.942446	0.923913	0.994737	0.968429	0.942032
860	0.942446	0.923913	0.994737	0.968429	0.942032
861	0.94964	0.923913	0.994737	0.969219	0.943495
862	0.94964	0.923913	0.994737	0.969219	0.943495
863	0.94964	0.923913	0.994737	0.969219	0.943495
864	0.94964	0.921196	0.994737	0.968429	0.942062
865	0.94964	0.923913	0.994737	0.969219	0.943495
866	0.94964	0.923913	0.994737	0.969219	0.943495
867	0.94964	0.923913	0.994737	0.969219	0.943495
868	0.94964	0.923913	0.994737	0.969219	0.943495
869	0.94964	0.923913	0.994737	0.969219	0.943495
870	0.94964	0.923913	0.994737	0.969219	0.943495
871	0.94964	0.923913	0.994737	0.969219	0.943495
872	0.94964	0.923913	0.994737	0.969219	0.943495
873	0.94964	0.923913	0.994737	0.969219	0.943495
874	0.94964	0.923913	0.994737	0.969219	0.943495
875	0.94964	0.923913	0.994737	0.969219	0.943495
876	0.94964	0.92663	0.994737	0.970008	0.944929
877	0.94964	0.92663	0.994737	0.970008	0.944929
878	0.94964	0.923913	0.994737	0.969219	0.943495
879	0.94964	0.923913	0.994737	0.969219	0.943495
880	0.94964	0.923913	0.994737	0.969219	0.943495
881	0.94964	0.923913	0.994737	0.969219	0.943495
882	0.942446	0.923913	0.994737	0.968429	0.942032
883	0.942446	0.921196	0.994737	0.96764	0.940599
884	0.942446	0.921196	0.994737	0.96764	0.940599
885	0.935252	0.923913	0.994737	0.96764	0.94057
886	0.935252	0.923913	0.994737	0.96764	0.94057
887	0.935252	0.923913	0.994737	0.96764	0.94057
888	0.935252	0.923913	0.994737	0.96764	0.94057
889	0.942446	0.923913	0.993421	0.96764	0.940543
890	0.942446	0.923913	0.993421	0.96764	0.940543
891	0.942446	0.923913	0.993421	0.96764	0.940543
892	0.942446	0.92663	0.993421	0.968429	0.941978
893	0.935252	0.929348	0.993421	0.968429	0.94195
894	0.935252	0.929348	0.993421	0.968429	0.94195
895	0.935252	0.929348	0.993421	0.968429	0.94195
896	0.935252	0.929348	0.993421	0.968429	0.94195
897	0.935252	0.929348	0.993421	0.968429	0.94195
898	0.935252	0.92663	0.993421	0.96764	0.940513
899	0.935252	0.921196	0.993421	0.966062	0.937643
900	0.935252	0.921196	0.993421	0.966062	0.937643

901	0.942446	0.921196	0.993421	0.966851	0.939108
902	0.942446	0.921196	0.993421	0.966851	0.939108
903	0.942446	0.918478	0.993421	0.966062	0.937675
904	0.942446	0.918478	0.993421	0.966062	0.937675
905	0.942446	0.915761	0.993421	0.965272	0.936243
906	0.942446	0.915761	0.993421	0.965272	0.936243
907	0.942446	0.918478	0.993421	0.966062	0.937675
908	0.942446	0.921196	0.993421	0.966851	0.939108
909	0.942446	0.921196	0.993421	0.966851	0.939108
910	0.942446	0.921196	0.993421	0.966851	0.939108
911	0.935252	0.921196	0.993421	0.966062	0.937643
912	0.935252	0.918478	0.993421	0.965272	0.936209
913	0.935252	0.918478	0.993421	0.965272	0.936209
914	0.935252	0.918478	0.993421	0.965272	0.936209
915	0.935252	0.918478	0.993421	0.965272	0.936209
916	0.935252	0.918478	0.993421	0.965272	0.936209
917	0.942446	0.918478	0.993421	0.966062	0.937675
918	0.942446	0.918478	0.993421	0.966062	0.937675
919	0.942446	0.918478	0.993421	0.966062	0.937675
920	0.942446	0.918478	0.993421	0.966062	0.937675
921	0.942446	0.918478	0.993421	0.966062	0.937675
922	0.942446	0.918478	0.993421	0.966062	0.937675
923	0.942446	0.918478	0.993421	0.966062	0.937675
924	0.942446	0.918478	0.993421	0.966062	0.937675
925	0.942446	0.918478	0.993421	0.966062	0.937675
926	0.942446	0.918478	0.993421	0.966062	0.937675
927	0.942446	0.918478	0.993421	0.966062	0.937675
928	0.942446	0.918478	0.993421	0.966062	0.937675
929	0.942446	0.918478	0.993421	0.966062	0.937675
930	0.942446	0.915761	0.993421	0.965272	0.936243
931	0.94964	0.915761	0.993421	0.966062	0.937709
932	0.94964	0.915761	0.993421	0.966062	0.937709
933	0.94964	0.915761	0.993421	0.966062	0.937709
934	0.94964	0.915761	0.993421	0.966062	0.937709
935	0.94964	0.915761	0.993421	0.966062	0.937709
936	0.94964	0.915761	0.993421	0.966062	0.937709
937	0.94964	0.915761	0.993421	0.966062	0.937709
938	0.94964	0.915761	0.993421	0.966062	0.937709
939	0.94964	0.915761	0.993421	0.966062	0.937709
940	0.94964	0.915761	0.993421	0.966062	0.937709
941	0.94964	0.915761	0.993421	0.966062	0.937709
942	0.94964	0.915761	0.994737	0.966851	0.9392
943	0.94964	0.915761	0.994737	0.966851	0.9392

944	0.94964	0.915761	0.994737	0.966851	0.9392
945	0.94964	0.915761	0.994737	0.966851	0.9392
946	0.94964	0.915761	0.994737	0.966851	0.9392
947	0.94964	0.918478	0.994737	0.96764	0.940631
948	0.94964	0.918478	0.994737	0.96764	0.940631
949	0.94964	0.918478	0.994737	0.96764	0.940631
950	0.94964	0.918478	0.994737	0.96764	0.940631
951	0.94964	0.918478	0.994737	0.96764	0.940631
952	0.94964	0.918478	0.994737	0.96764	0.940631
953	0.94964	0.918478	0.994737	0.96764	0.940631
954	0.94964	0.915761	0.994737	0.966851	0.9392
955	0.94964	0.915761	0.994737	0.966851	0.9392
956	0.94964	0.915761	0.994737	0.966851	0.9392
957	0.94964	0.915761	0.994737	0.966851	0.9392
958	0.94964	0.915761	0.994737	0.966851	0.9392
959	0.94964	0.915761	0.994737	0.966851	0.9392
960	0.94964	0.918478	0.994737	0.96764	0.940631
961	0.94964	0.921196	0.994737	0.968429	0.942062
962	0.94964	0.921196	0.994737	0.968429	0.942062
963	0.94964	0.921196	0.994737	0.968429	0.942062
964	0.94964	0.921196	0.994737	0.968429	0.942062
965	0.94964	0.921196	0.994737	0.968429	0.942062
966	0.94964	0.921196	0.994737	0.968429	0.942062
967	0.94964	0.921196	0.993421	0.96764	0.940574
968	0.94964	0.921196	0.993421	0.96764	0.940574
969	0.94964	0.921196	0.993421	0.96764	0.940574
970	0.94964	0.921196	0.993421	0.96764	0.940574
971	0.94964	0.921196	0.993421	0.96764	0.940574
972	0.94964	0.921196	0.993421	0.96764	0.940574
973	0.94964	0.921196	0.993421	0.96764	0.940574
974	0.94964	0.921196	0.993421	0.96764	0.940574
975	0.94964	0.921196	0.993421	0.96764	0.940574
976	0.94964	0.921196	0.993421	0.96764	0.940574
977	0.94964	0.921196	0.993421	0.96764	0.940574
978	0.94964	0.921196	0.993421	0.96764	0.940574
979	0.94964	0.921196	0.993421	0.96764	0.940574
980	0.94964	0.921196	0.993421	0.96764	0.940574
981	0.94964	0.921196	0.993421	0.96764	0.940574
982	0.94964	0.921196	0.993421	0.96764	0.940574
983	0.94964	0.921196	0.993421	0.96764	0.940574
984	0.94964	0.921196	0.993421	0.96764	0.940574
985	0.94964	0.921196	0.993421	0.96764	0.940574
986	0.94964	0.921196	0.993421	0.96764	0.940574

987	0.94964	0.921196	0.993421	0.96764	0.940574
988	0.94964	0.921196	0.993421	0.96764	0.940574
989	0.94964	0.921196	0.993421	0.96764	0.940574
990	0.94964	0.921196	0.993421	0.96764	0.940574
991	0.94964	0.921196	0.993421	0.96764	0.940574
992	0.94964	0.921196	0.993421	0.96764	0.940574
993	0.94964	0.921196	0.993421	0.96764	0.940574
994	0.94964	0.921196	0.993421	0.96764	0.940574
995	0.94964	0.921196	0.993421	0.96764	0.940574
996	0.94964	0.921196	0.993421	0.96764	0.940574
997	0.94964	0.918478	0.993421	0.966851	0.939141
998	0.94964	0.918478	0.993421	0.966851	0.939141
999	0.94964	0.918478	0.993421	0.966851	0.939141
1000	0.94964	0.918478	0.993421	0.966851	0.939141

Table S5. The accuracies for different races and total prediction accuracy obtained by IFS method and Dagging

Number of features	Accuracy for American race	Accuracy for Asian race	Accuracy for European race	Overall prediction accuracy	MCC
4	0.352518	0.67663	0.992105	0.830308	0.683995
5	0.633094	0.679348	0.992105	0.861878	0.745944
6	0.661871	0.76087	0.988158	0.886346	0.790158
7	0.726619	0.769022	0.993421	0.898974	0.814795
8	0.748201	0.796196	0.992105	0.908445	0.832248
9	0.805755	0.798913	0.996053	0.917916	0.85039
10	0.81295	0.820652	0.994737	0.92423	0.861601
11	0.805755	0.836957	0.996053	0.928966	0.870143
12	0.827338	0.88587	0.997368	0.94633	0.901839
13	0.820144	0.88587	0.997368	0.945541	0.900346
14	0.81295	0.883152	0.997368	0.943962	0.897465
15	0.805755	0.891304	0.997368	0.945541	0.900283
16	0.81295	0.875	0.996053	0.940805	0.891587
17	0.81295	0.899457	0.997368	0.948698	0.90602
18	0.805755	0.907609	0.998684	0.951066	0.910443
19	0.841727	0.896739	0.998684	0.951855	0.912001
20	0.863309	0.902174	0.998684	0.955801	0.919243
21	0.848921	0.894022	0.998684	0.951855	0.912034
22	0.848921	0.902174	0.997368	0.953433	0.914753
23	0.841727	0.913043	0.997368	0.955801	0.919007
24	0.863309	0.918478	0.997368	0.959747	0.92623
25	0.863309	0.923913	1	0.962904	0.932181
26	0.863309	0.92663	1	0.963694	0.93361
27	0.863309	0.92663	1	0.963694	0.93361
28	0.863309	0.932065	1	0.965272	0.936469
29	0.848921	0.932065	1	0.963694	0.933575
30	0.870504	0.929348	1	0.965272	0.936487
31	0.884892	0.929348	1	0.966851	0.939385
32	0.906475	0.92663	0.998684	0.96764	0.940792
33	0.899281	0.934783	1	0.970008	0.945143
34	0.906475	0.932065	1	0.970008	0.945164
35	0.913669	0.932065	1	0.970797	0.946615
36	0.906475	0.94837	1	0.974743	0.953751
37	0.906475	0.953804	0.998684	0.975533	0.955116
38	0.906475	0.959239	1	0.977901	0.959496
39	0.906475	0.959239	1	0.977901	0.959496
40	0.920863	0.970109	1	0.982636	0.96815

41	0.906475	0.967391	1	0.980268	0.963816
42	0.913669	0.967391	1	0.981058	0.965262
43	0.899281	0.964674	0.998684	0.977901	0.959425
44	0.899281	0.970109	1	0.980268	0.963812
45	0.906475	0.970109	1	0.981058	0.965258
46	0.906475	0.967391	1	0.980268	0.963816
47	0.906475	0.970109	1	0.981058	0.965258
48	0.899281	0.967391	1	0.979479	0.96237
49	0.913669	0.970109	0.997368	0.980268	0.963708
50	0.899281	0.967391	1	0.979479	0.96237
51	0.906475	0.970109	0.998684	0.980268	0.963756
52	0.906475	0.972826	1	0.981847	0.966701
53	0.906475	0.964674	1	0.979479	0.962375
54	0.913669	0.970109	0.997368	0.980268	0.963708
55	0.906475	0.967391	0.998684	0.979479	0.962311
56	0.899281	0.964674	0.998684	0.977901	0.95942
57	0.928058	0.970109	0.998684	0.982636	0.968101
58	0.928058	0.970109	1	0.983425	0.969597
59	0.928058	0.967391	1	0.982636	0.968156
60	0.942446	0.964674	0.998684	0.982636	0.968117
61	0.928058	0.964674	1	0.981847	0.966716
62	0.920863	0.970109	0.998684	0.981847	0.966652
63	0.928058	0.970109	0.998684	0.982636	0.968101
64	0.928058	0.972826	0.998684	0.983425	0.969546
65	0.935252	0.967391	0.998684	0.982636	0.968108
66	0.928058	0.967391	0.997368	0.981058	0.965163
67	0.935252	0.970109	0.997368	0.982636	0.968061
68	0.942446	0.975543	0.996053	0.984215	0.970928
69	0.935252	0.975543	0.996053	0.983425	0.969475
70	0.928058	0.975543	0.997368	0.983425	0.969505
71	0.935252	0.975543	0.997368	0.984215	0.97096
72	0.942446	0.972826	0.997368	0.984215	0.97096
73	0.935252	0.978261	0.997368	0.985004	0.972405
74	0.935252	0.972826	0.997368	0.983425	0.969508
75	0.928058	0.972826	0.998684	0.983425	0.969546
76	0.928058	0.970109	0.997368	0.981847	0.966609
77	0.928058	0.972826	0.997368	0.982636	0.968057
78	0.928058	0.972826	0.997368	0.982636	0.968057
79	0.942446	0.972826	0.998684	0.985004	0.972446
80	0.928058	0.970109	0.998684	0.982636	0.968101
81	0.935252	0.972826	0.998684	0.984215	0.970996
82	0.920863	0.975543	0.998684	0.983425	0.969543
83	0.928058	0.970109	0.997368	0.981847	0.966609

84	0.920863	0.970109	0.998684	0.981847	0.966652
85	0.928058	0.980978	0.998684	0.985793	0.973887
86	0.920863	0.978261	0.996053	0.982636	0.968022
87	0.920863	0.978261	1	0.985004	0.972481
88	0.935252	0.978261	0.997368	0.985004	0.972405
89	0.935252	0.975543	0.997368	0.984215	0.970956
90	0.928058	0.975543	0.996053	0.982636	0.968022
91	0.935252	0.978261	0.997368	0.985004	0.972405
92	0.920863	0.972826	0.997368	0.981847	0.966606
93	0.935252	0.975543	0.997368	0.984215	0.970956
94	0.935252	0.978261	0.997368	0.985004	0.972405
95	0.935252	0.978261	0.996053	0.984215	0.970926
96	0.920863	0.980978	0.998684	0.985004	0.972439
97	0.920863	0.978261	0.998684	0.984215	0.970991
98	0.928058	0.978261	0.998684	0.985004	0.972439
99	0.94964	0.978261	0.997368	0.986582	0.975308
100	0.928058	0.978261	0.997368	0.984215	0.970954
101	0.928058	0.980978	0.997368	0.985004	0.972405
102	0.920863	0.975543	0.997368	0.982636	0.968054
103	0.920863	0.975543	0.997368	0.982636	0.968054
104	0.928058	0.975543	0.998684	0.984215	0.970992
105	0.935252	0.980978	0.998684	0.986582	0.975335
106	0.935252	0.978261	0.997368	0.985004	0.972405
107	0.920863	0.980978	0.997368	0.984215	0.970955
108	0.928058	0.975543	0.997368	0.983425	0.969505
109	0.928058	0.978261	0.997368	0.984215	0.970954
110	0.920863	0.975543	0.998684	0.983425	0.969543
111	0.920863	0.978261	0.997368	0.983425	0.969504
112	0.928058	0.978261	0.997368	0.984215	0.970954
113	0.935252	0.978261	0.997368	0.985004	0.972405
114	0.920863	0.978261	0.997368	0.983425	0.969504
115	0.920863	0.980978	0.997368	0.984215	0.970955
116	0.920863	0.978261	0.997368	0.983425	0.969504
117	0.920863	0.978261	1	0.985004	0.972481
118	0.920863	0.972826	0.997368	0.981847	0.966606
119	0.920863	0.975543	1	0.984215	0.971036
120	0.913669	0.980978	0.998684	0.984215	0.970991
121	0.928058	0.972826	0.997368	0.982636	0.968057
122	0.928058	0.980978	0.998684	0.985793	0.973887
123	0.913669	0.978261	0.998684	0.983425	0.969543
124	0.928058	0.972826	0.998684	0.983425	0.969546
125	0.913669	0.978261	0.998684	0.983425	0.969543
126	0.920863	0.978261	1	0.985004	0.972481

127	0.928058	0.978261	0.998684	0.985004	0.972439
128	0.920863	0.975543	1	0.984215	0.971036
129	0.913669	0.980978	0.996053	0.982636	0.968024
130	0.920863	0.975543	1	0.984215	0.971036
131	0.920863	0.972826	1	0.983425	0.969593
132	0.928058	0.978261	0.998684	0.985004	0.972439
133	0.913669	0.978261	0.998684	0.983425	0.969543
134	0.913669	0.975543	0.997368	0.981847	0.966605
135	0.913669	0.978261	1	0.984215	0.971035
136	0.913669	0.975543	0.997368	0.981847	0.966605
137	0.906475	0.980978	0.997368	0.982636	0.968057
138	0.928058	0.975543	1	0.985004	0.972483
139	0.920863	0.983696	0.997368	0.985004	0.972407
140	0.913669	0.980978	1	0.985004	0.972481
141	0.913669	0.978261	0.998684	0.983425	0.969543
142	0.928058	0.980978	1	0.986582	0.975373
143	0.935252	0.980978	0.998684	0.986582	0.975335
144	0.920863	0.975543	0.998684	0.983425	0.969543
145	0.920863	0.980978	0.998684	0.985004	0.972439
146	0.920863	0.978261	0.997368	0.983425	0.969504
147	0.913669	0.975543	1	0.983425	0.96959
148	0.920863	0.983696	1	0.986582	0.975373
149	0.913669	0.978261	0.998684	0.983425	0.969543
150	0.920863	0.978261	0.998684	0.984215	0.970991
151	0.920863	0.980978	0.997368	0.984215	0.970955
152	0.928058	0.978261	1	0.985793	0.973927
153	0.920863	0.980978	1	0.985793	0.973927
154	0.920863	0.983696	1	0.986582	0.975373
155	0.928058	0.983696	1	0.987372	0.976819
156	0.928058	0.978261	1	0.985793	0.973927
157	0.920863	0.980978	0.997368	0.984215	0.970955
158	0.928058	0.975543	1	0.985004	0.972483
159	0.920863	0.986413	1	0.987372	0.976821
160	0.913669	0.975543	0.998684	0.982636	0.968095
161	0.928058	0.980978	1	0.986582	0.975373
162	0.942446	0.975543	1	0.986582	0.975378
163	0.920863	0.978261	0.998684	0.984215	0.970991
164	0.920863	0.978261	1	0.985004	0.972481
165	0.928058	0.978261	0.997368	0.984215	0.970954
166	0.920863	0.980978	0.996053	0.983425	0.969475
167	0.920863	0.975543	1	0.984215	0.971036
168	0.935252	0.980978	1	0.987372	0.976819
169	0.920863	0.978261	0.998684	0.984215	0.970991

170	0.928058	0.975543	1	0.985004	0.972483
171	0.913669	0.978261	1	0.984215	0.971035
172	0.913669	0.978261	1	0.984215	0.971035
173	0.935252	0.978261	1	0.986582	0.975374
174	0.928058	0.972826	1	0.984215	0.97104
175	0.928058	0.980978	1	0.986582	0.975373
176	0.920863	0.980978	1	0.985793	0.973927
177	0.920863	0.980978	1	0.985793	0.973927
178	0.920863	0.978261	0.998684	0.984215	0.970991
179	0.913669	0.980978	1	0.985004	0.972481
180	0.935252	0.975543	0.997368	0.984215	0.970956
181	0.928058	0.980978	0.998684	0.985793	0.973887
182	0.928058	0.975543	1	0.985004	0.972483
183	0.920863	0.975543	0.998684	0.983425	0.969543
184	0.928058	0.975543	0.998684	0.984215	0.970992
185	0.920863	0.975543	1	0.984215	0.971036
186	0.920863	0.980978	0.998684	0.985004	0.972439
187	0.928058	0.978261	0.998684	0.985004	0.972439
188	0.920863	0.975543	0.998684	0.983425	0.969543
189	0.928058	0.978261	1	0.985793	0.973927
190	0.928058	0.980978	1	0.986582	0.975373
191	0.928058	0.975543	1	0.985004	0.972483
192	0.928058	0.972826	1	0.984215	0.97104
193	0.913669	0.975543	1	0.983425	0.96959
194	0.920863	0.975543	1	0.984215	0.971036
195	0.920863	0.975543	0.998684	0.983425	0.969543
196	0.913669	0.983696	0.998684	0.985004	0.972441
197	0.920863	0.978261	1	0.985004	0.972481
198	0.942446	0.975543	0.998684	0.985793	0.973891
199	0.913669	0.978261	0.998684	0.983425	0.969543
200	0.906475	0.975543	1	0.982636	0.968145
201	0.920863	0.983696	0.997368	0.985004	0.972407
202	0.913669	0.975543	1	0.983425	0.96959
203	0.913669	0.978261	1	0.984215	0.971035
204	0.920863	0.980978	1	0.985793	0.973927
205	0.920863	0.980978	0.998684	0.985004	0.972439
206	0.913669	0.978261	1	0.984215	0.971035
207	0.913669	0.978261	1	0.984215	0.971035
208	0.920863	0.975543	1	0.984215	0.971036
209	0.920863	0.980978	1	0.985793	0.973927
210	0.913669	0.983696	0.998684	0.985004	0.972441
211	0.913669	0.980978	0.998684	0.984215	0.970991
212	0.928058	0.980978	0.998684	0.985793	0.973887

213	0.935252	0.978261	1	0.986582	0.975374
214	0.935252	0.978261	1	0.986582	0.975374
215	0.920863	0.978261	1	0.985004	0.972481
216	0.928058	0.978261	0.998684	0.985004	0.972439
217	0.928058	0.978261	1	0.985793	0.973927
218	0.928058	0.980978	0.998684	0.985793	0.973887
219	0.920863	0.975543	0.998684	0.983425	0.969543
220	0.920863	0.975543	0.998684	0.983425	0.969543
221	0.920863	0.980978	1	0.985793	0.973927
222	0.935252	0.975543	0.998684	0.985004	0.972441
223	0.913669	0.978261	0.998684	0.983425	0.969543
224	0.913669	0.980978	0.998684	0.984215	0.970991
225	0.920863	0.975543	0.998684	0.983425	0.969543
226	0.913669	0.978261	1	0.984215	0.971035
227	0.928058	0.978261	0.997368	0.984215	0.970954
228	0.920863	0.978261	1	0.985004	0.972481
229	0.920863	0.972826	0.998684	0.982636	0.968097
230	0.920863	0.978261	1	0.985004	0.972481
231	0.913669	0.980978	0.998684	0.984215	0.970991
232	0.920863	0.983696	0.998684	0.985793	0.973888
233	0.920863	0.980978	0.998684	0.985004	0.972439
234	0.913669	0.980978	0.998684	0.984215	0.970991
235	0.920863	0.983696	0.998684	0.985793	0.973888
236	0.913669	0.978261	0.998684	0.983425	0.969543
237	0.906475	0.978261	0.998684	0.982636	0.968096
238	0.920863	0.978261	0.998684	0.984215	0.970991
239	0.920863	0.983696	0.998684	0.985793	0.973888
240	0.928058	0.978261	0.998684	0.985004	0.972439
241	0.920863	0.983696	1	0.986582	0.975373
242	0.935252	0.980978	0.998684	0.986582	0.975335
243	0.935252	0.980978	0.998684	0.986582	0.975335
244	0.920863	0.980978	0.998684	0.985004	0.972439
245	0.935252	0.980978	1	0.987372	0.976819
246	0.920863	0.978261	0.998684	0.984215	0.970991
247	0.928058	0.983696	1	0.987372	0.976819
248	0.928058	0.980978	1	0.986582	0.975373
249	0.935252	0.980978	1	0.987372	0.976819
250	0.920863	0.975543	0.998684	0.983425	0.969543
251	0.928058	0.980978	1	0.986582	0.975373
252	0.935252	0.978261	1	0.986582	0.975374
253	0.928058	0.978261	0.998684	0.985004	0.972439
254	0.920863	0.980978	1	0.985793	0.973927
255	0.928058	0.978261	0.998684	0.985004	0.972439

256	0.942446	0.983696	1	0.98895	0.979712
257	0.913669	0.980978	0.998684	0.984215	0.970991
258	0.913669	0.978261	1	0.984215	0.971035
259	0.935252	0.980978	0.997368	0.985793	0.973855
260	0.920863	0.978261	1	0.985004	0.972481
261	0.920863	0.975543	0.997368	0.982636	0.968054
262	0.913669	0.978261	0.998684	0.983425	0.969543
263	0.913669	0.980978	0.998684	0.984215	0.970991
264	0.920863	0.980978	1	0.985793	0.973927
265	0.935252	0.980978	0.997368	0.985793	0.973855
266	0.928058	0.978261	0.998684	0.985004	0.972439
267	0.913669	0.980978	1	0.985004	0.972481
268	0.920863	0.980978	0.997368	0.984215	0.970955
269	0.935252	0.978261	0.998684	0.985793	0.973888
270	0.928058	0.980978	0.998684	0.985793	0.973887
271	0.935252	0.978261	1	0.986582	0.975374
272	0.928058	0.980978	0.998684	0.985793	0.973887
273	0.906475	0.983696	1	0.985004	0.972484
274	0.928058	0.983696	1	0.987372	0.976819
275	0.928058	0.980978	1	0.986582	0.975373
276	0.913669	0.980978	0.998684	0.984215	0.970991
277	0.935252	0.980978	0.998684	0.986582	0.975335
278	0.928058	0.980978	0.998684	0.985793	0.973887
279	0.920863	0.978261	0.997368	0.983425	0.969504
280	0.920863	0.978261	0.998684	0.984215	0.970991
281	0.913669	0.980978	0.998684	0.984215	0.970991
282	0.928058	0.980978	0.997368	0.985004	0.972405
283	0.935252	0.983696	0.997368	0.986582	0.975306
284	0.928058	0.980978	1	0.986582	0.975373
285	0.935252	0.983696	0.997368	0.986582	0.975306
286	0.928058	0.980978	0.998684	0.985793	0.973887
287	0.935252	0.980978	0.998684	0.986582	0.975335
288	0.935252	0.978261	0.997368	0.985004	0.972405
289	0.935252	0.978261	0.997368	0.985004	0.972405
290	0.935252	0.978261	1	0.986582	0.975374
291	0.942446	0.980978	0.997368	0.986582	0.975306
292	0.935252	0.980978	0.998684	0.986582	0.975335
293	0.942446	0.978261	0.998684	0.986582	0.975337
294	0.942446	0.980978	0.998684	0.987372	0.976784
295	0.928058	0.978261	0.998684	0.985004	0.972439
296	0.928058	0.980978	0.997368	0.985004	0.972405
297	0.928058	0.978261	0.997368	0.984215	0.970954
298	0.935252	0.980978	0.997368	0.985793	0.973855

299	0.935252	0.980978	1	0.987372	0.976819
300	0.94964	0.980978	1	0.98895	0.979714
301	0.942446	0.980978	1	0.988161	0.978266
302	0.956835	0.978261	1	0.98895	0.979719
303	0.935252	0.980978	0.998684	0.986582	0.975335
304	0.935252	0.983696	0.998684	0.987372	0.976784
305	0.942446	0.978261	0.998684	0.986582	0.975337
306	0.928058	0.978261	1	0.985793	0.973927
307	0.935252	0.978261	1	0.986582	0.975374
308	0.942446	0.978261	1	0.987372	0.976822
309	0.906475	0.978261	0.998684	0.982636	0.968096
310	0.94964	0.980978	1	0.98895	0.979714
311	0.928058	0.980978	1	0.986582	0.975373
312	0.928058	0.980978	1	0.986582	0.975373
313	0.956835	0.980978	0.998684	0.98895	0.979685
314	0.942446	0.975543	0.998684	0.985793	0.973891
315	0.935252	0.978261	1	0.986582	0.975374
316	0.920863	0.978261	0.998684	0.984215	0.970991
317	0.920863	0.978261	1	0.985004	0.972481
318	0.928058	0.980978	0.998684	0.985793	0.973887
319	0.913669	0.978261	1	0.984215	0.971035
320	0.935252	0.980978	0.998684	0.986582	0.975335
321	0.942446	0.975543	0.998684	0.985793	0.973891
322	0.920863	0.975543	1	0.984215	0.971036
323	0.920863	0.978261	1	0.985004	0.972481
324	0.94964	0.978261	1	0.988161	0.97827
325	0.935252	0.972826	0.998684	0.984215	0.970996
326	0.935252	0.978261	0.997368	0.985004	0.972405
327	0.942446	0.980978	0.998684	0.987372	0.976784
328	0.935252	0.978261	0.998684	0.985793	0.973888
329	0.928058	0.978261	1	0.985793	0.973927
330	0.920863	0.980978	1	0.985793	0.973927
331	0.935252	0.978261	0.998684	0.985793	0.973888
332	0.942446	0.980978	1	0.988161	0.978266
333	0.935252	0.978261	1	0.986582	0.975374
334	0.928058	0.975543	1	0.985004	0.972483
335	0.928058	0.978261	1	0.985793	0.973927
336	0.913669	0.980978	0.998684	0.984215	0.970991
337	0.928058	0.978261	0.998684	0.985004	0.972439
338	0.942446	0.978261	1	0.987372	0.976822
339	0.935252	0.980978	1	0.987372	0.976819
340	0.942446	0.980978	1	0.988161	0.978266
341	0.920863	0.980978	1	0.985793	0.973927

342	0.935252	0.978261	0.998684	0.985793	0.973888
343	0.928058	0.980978	0.997368	0.985004	0.972405
344	0.935252	0.978261	1	0.986582	0.975374
345	0.942446	0.972826	0.997368	0.984215	0.97096
346	0.928058	0.983696	0.998684	0.986582	0.975336
347	0.935252	0.978261	0.998684	0.985793	0.973888
348	0.942446	0.975543	0.998684	0.985793	0.973891
349	0.956835	0.980978	1	0.98974	0.981163
350	0.935252	0.980978	1	0.987372	0.976819
351	0.94964	0.978261	0.998684	0.987372	0.976787
352	0.94964	0.975543	0.998684	0.986582	0.975341
353	0.942446	0.980978	0.998684	0.987372	0.976784
354	0.928058	0.980978	0.998684	0.985793	0.973887
355	0.920863	0.975543	1	0.984215	0.971036
356	0.94964	0.978261	0.998684	0.987372	0.976787
357	0.942446	0.980978	0.998684	0.987372	0.976784
358	0.942446	0.978261	0.997368	0.985793	0.973856
359	0.94964	0.975543	1	0.987372	0.976826
360	0.935252	0.978261	0.997368	0.985004	0.972405
361	0.935252	0.978261	0.998684	0.985793	0.973888
362	0.942446	0.980978	0.998684	0.987372	0.976784
363	0.935252	0.978261	1	0.986582	0.975374
364	0.935252	0.980978	1	0.987372	0.976819
365	0.94964	0.978261	0.998684	0.987372	0.976787
366	0.942446	0.978261	1	0.987372	0.976822
367	0.935252	0.980978	1	0.987372	0.976819
368	0.913669	0.978261	1	0.984215	0.971035
369	0.935252	0.975543	0.998684	0.985004	0.972441
370	0.935252	0.975543	1	0.985793	0.97393
371	0.94964	0.978261	1	0.988161	0.97827
372	0.928058	0.975543	1	0.985004	0.972483
373	0.942446	0.975543	0.998684	0.985793	0.973891
374	0.920863	0.978261	0.998684	0.984215	0.970991
375	0.935252	0.975543	0.998684	0.985004	0.972441
376	0.928058	0.980978	0.998684	0.985793	0.973887
377	0.920863	0.975543	1	0.984215	0.971036
378	0.942446	0.975543	1	0.986582	0.975378
379	0.928058	0.975543	1	0.985004	0.972483
380	0.935252	0.978261	0.998684	0.985793	0.973888
381	0.935252	0.978261	1	0.986582	0.975374
382	0.928058	0.975543	0.998684	0.984215	0.970992
383	0.935252	0.975543	1	0.985793	0.97393
384	0.935252	0.972826	1	0.985004	0.972487

385	0.920863	0.978261	0.997368	0.983425	0.969504
386	0.935252	0.978261	0.998684	0.985793	0.973888
387	0.94964	0.975543	1	0.987372	0.976826
388	0.928058	0.978261	1	0.985793	0.973927
389	0.935252	0.978261	1	0.986582	0.975374
390	0.942446	0.975543	1	0.986582	0.975378
391	0.94964	0.978261	0.997368	0.986582	0.975308
392	0.935252	0.978261	0.997368	0.985004	0.972405
393	0.942446	0.978261	0.998684	0.986582	0.975337
394	0.942446	0.978261	1	0.987372	0.976822
395	0.935252	0.978261	0.998684	0.985793	0.973888
396	0.942446	0.978261	0.998684	0.986582	0.975337
397	0.928058	0.975543	1	0.985004	0.972483
398	0.928058	0.978261	1	0.985793	0.973927
399	0.942446	0.972826	0.998684	0.985004	0.972446
400	0.935252	0.978261	0.998684	0.985793	0.973888
401	0.928058	0.978261	0.997368	0.984215	0.970954
402	0.942446	0.978261	0.997368	0.985793	0.973856
403	0.94964	0.975543	1	0.987372	0.976826
404	0.942446	0.978261	0.998684	0.986582	0.975337
405	0.935252	0.975543	0.997368	0.984215	0.970956
406	0.920863	0.972826	0.998684	0.982636	0.968097
407	0.928058	0.978261	1	0.985793	0.973927
408	0.94964	0.972826	0.998684	0.985793	0.973896
409	0.942446	0.978261	0.998684	0.986582	0.975337
410	0.942446	0.978261	0.998684	0.986582	0.975337
411	0.94964	0.970109	0.998684	0.985004	0.972453
412	0.94964	0.978261	1	0.988161	0.97827
413	0.94964	0.978261	0.998684	0.987372	0.976787
414	0.935252	0.972826	0.998684	0.984215	0.970996
415	0.942446	0.978261	0.998684	0.986582	0.975337
416	0.94964	0.972826	0.997368	0.985004	0.972413
417	0.928058	0.975543	0.998684	0.984215	0.970992
418	0.935252	0.978261	0.998684	0.985793	0.973888
419	0.942446	0.975543	0.998684	0.985793	0.973891
420	0.928058	0.978261	0.997368	0.984215	0.970954
421	0.935252	0.980978	1	0.987372	0.976819
422	0.935252	0.972826	0.998684	0.984215	0.970996
423	0.928058	0.975543	0.997368	0.983425	0.969505
424	0.94964	0.978261	0.998684	0.987372	0.976787
425	0.928058	0.972826	0.998684	0.983425	0.969546
426	0.942446	0.980978	1	0.988161	0.978266
427	0.935252	0.975543	1	0.985793	0.97393

428	0.928058	0.978261	0.998684	0.985004	0.972439
429	0.935252	0.980978	1	0.987372	0.976819
430	0.956835	0.978261	1	0.98895	0.979719
431	0.942446	0.978261	0.998684	0.986582	0.975337
432	0.942446	0.975543	0.998684	0.985793	0.973891
433	0.935252	0.980978	0.998684	0.986582	0.975335
434	0.942446	0.978261	1	0.987372	0.976822
435	0.935252	0.975543	1	0.985793	0.97393
436	0.935252	0.978261	0.998684	0.985793	0.973888
437	0.942446	0.980978	1	0.988161	0.978266
438	0.935252	0.975543	0.997368	0.984215	0.970956
439	0.928058	0.980978	0.998684	0.985793	0.973887
440	0.928058	0.975543	0.998684	0.984215	0.970992
441	0.928058	0.972826	0.998684	0.983425	0.969546
442	0.94964	0.975543	0.998684	0.986582	0.975341
443	0.935252	0.978261	0.998684	0.985793	0.973888
444	0.928058	0.978261	0.997368	0.984215	0.970954
445	0.942446	0.975543	0.998684	0.985793	0.973891
446	0.928058	0.972826	0.998684	0.983425	0.969546
447	0.94964	0.975543	0.998684	0.986582	0.975341
448	0.928058	0.975543	0.998684	0.984215	0.970992
449	0.956835	0.975543	0.998684	0.987372	0.976792
450	0.928058	0.975543	0.998684	0.984215	0.970992
451	0.920863	0.978261	0.998684	0.984215	0.970991
452	0.935252	0.978261	1	0.986582	0.975374
453	0.935252	0.980978	0.998684	0.986582	0.975335
454	0.935252	0.978261	0.997368	0.985004	0.972405
455	0.935252	0.978261	1	0.986582	0.975374
456	0.935252	0.978261	0.998684	0.985793	0.973888
457	0.942446	0.980978	0.998684	0.987372	0.976784
458	0.935252	0.980978	0.998684	0.986582	0.975335
459	0.935252	0.975543	0.997368	0.984215	0.970956
460	0.928058	0.978261	1	0.985793	0.973927
461	0.935252	0.972826	0.998684	0.984215	0.970996
462	0.928058	0.975543	0.997368	0.983425	0.969505
463	0.928058	0.978261	0.997368	0.984215	0.970954
464	0.942446	0.978261	0.997368	0.985793	0.973856
465	0.935252	0.983696	0.998684	0.987372	0.976784
466	0.935252	0.978261	0.998684	0.985793	0.973888
467	0.935252	0.983696	0.998684	0.987372	0.976784
468	0.935252	0.980978	0.998684	0.986582	0.975335
469	0.935252	0.980978	1	0.987372	0.976819
470	0.928058	0.972826	0.998684	0.983425	0.969546

471	0.935252	0.975543	0.998684	0.985004	0.972441
472	0.928058	0.980978	0.998684	0.985793	0.973887
473	0.942446	0.980978	0.998684	0.987372	0.976784
474	0.935252	0.975543	0.998684	0.985004	0.972441
475	0.94964	0.978261	0.998684	0.987372	0.976787
476	0.942446	0.972826	1	0.985793	0.973935
477	0.935252	0.970109	1	0.984215	0.971045
478	0.920863	0.978261	0.998684	0.984215	0.970991
479	0.935252	0.978261	0.998684	0.985793	0.973888
480	0.928058	0.978261	0.998684	0.985004	0.972439
481	0.942446	0.978261	1	0.987372	0.976822
482	0.942446	0.978261	0.998684	0.986582	0.975337
483	0.928058	0.978261	0.998684	0.985004	0.972439
484	0.935252	0.978261	0.998684	0.985793	0.973888
485	0.935252	0.978261	0.998684	0.985793	0.973888
486	0.942446	0.975543	0.997368	0.985004	0.972408
487	0.942446	0.978261	0.998684	0.986582	0.975337
488	0.928058	0.980978	0.998684	0.985793	0.973887
489	0.94964	0.980978	1	0.98895	0.979714
490	0.920863	0.975543	0.998684	0.983425	0.969543
491	0.913669	0.983696	0.998684	0.985004	0.972441
492	0.928058	0.975543	1	0.985004	0.972483
493	0.928058	0.983696	1	0.987372	0.976819
494	0.928058	0.972826	0.998684	0.983425	0.969546
495	0.920863	0.975543	1	0.984215	0.971036
496	0.935252	0.975543	0.997368	0.984215	0.970956
497	0.942446	0.978261	0.998684	0.986582	0.975337
498	0.928058	0.980978	0.998684	0.985793	0.973887
499	0.956835	0.978261	0.998684	0.988161	0.978238
500	0.928058	0.980978	0.998684	0.985793	0.973887
501	0.935252	0.975543	0.998684	0.985004	0.972441
502	0.942446	0.975543	0.997368	0.985004	0.972408
503	0.942446	0.978261	0.998684	0.986582	0.975337
504	0.935252	0.978261	0.998684	0.985793	0.973888
505	0.928058	0.978261	0.998684	0.985004	0.972439
506	0.942446	0.975543	0.998684	0.985793	0.973891
507	0.928058	0.975543	0.997368	0.983425	0.969505
508	0.920863	0.978261	0.997368	0.983425	0.969504
509	0.94964	0.978261	0.998684	0.987372	0.976787
510	0.935252	0.978261	1	0.986582	0.975374
511	0.928058	0.975543	1	0.985004	0.972483
512	0.942446	0.972826	0.998684	0.985004	0.972446
513	0.942446	0.978261	0.998684	0.986582	0.975337

514	0.935252	0.975543	0.998684	0.985004	0.972441
515	0.935252	0.978261	0.998684	0.985793	0.973888
516	0.920863	0.975543	0.998684	0.983425	0.969543
517	0.928058	0.980978	1	0.986582	0.975373
518	0.942446	0.978261	0.997368	0.985793	0.973856
519	0.942446	0.978261	1	0.987372	0.976822
520	0.920863	0.980978	0.998684	0.985004	0.972439
521	0.942446	0.980978	0.998684	0.987372	0.976784
522	0.94964	0.978261	1	0.988161	0.97827
523	0.94964	0.975543	0.998684	0.986582	0.975341
524	0.935252	0.980978	0.998684	0.986582	0.975335
525	0.935252	0.975543	0.998684	0.985004	0.972441
526	0.942446	0.978261	1	0.987372	0.976822
527	0.935252	0.975543	0.998684	0.985004	0.972441
528	0.935252	0.978261	1	0.986582	0.975374
529	0.935252	0.978261	0.998684	0.985793	0.973888
530	0.935252	0.978261	0.997368	0.985004	0.972405
531	0.928058	0.980978	0.997368	0.985004	0.972405
532	0.928058	0.975543	1	0.985004	0.972483
533	0.935252	0.972826	1	0.985004	0.972487
534	0.964029	0.980978	0.998684	0.98974	0.981136
535	0.935252	0.975543	0.998684	0.985004	0.972441
536	0.942446	0.978261	0.998684	0.986582	0.975337
537	0.920863	0.975543	1	0.984215	0.971036
538	0.928058	0.975543	0.997368	0.983425	0.969505
539	0.935252	0.972826	0.998684	0.984215	0.970996
540	0.935252	0.983696	1	0.988161	0.978265
541	0.94964	0.978261	0.998684	0.987372	0.976787
542	0.935252	0.978261	0.998684	0.985793	0.973888
543	0.913669	0.978261	1	0.984215	0.971035
544	0.94964	0.980978	1	0.98895	0.979714
545	0.935252	0.972826	0.998684	0.984215	0.970996
546	0.956835	0.980978	1	0.98974	0.981163
547	0.942446	0.975543	0.997368	0.985004	0.972408
548	0.942446	0.978261	1	0.987372	0.976822
549	0.928058	0.980978	0.998684	0.985793	0.973887
550	0.956835	0.978261	0.998684	0.988161	0.978238
551	0.942446	0.978261	1	0.987372	0.976822
552	0.94964	0.975543	1	0.987372	0.976826
553	0.94964	0.975543	0.998684	0.986582	0.975341
554	0.94964	0.978261	0.998684	0.987372	0.976787
555	0.942446	0.983696	0.998684	0.988161	0.978233
556	0.942446	0.978261	1	0.987372	0.976822

557	0.935252	0.980978	0.998684	0.986582	0.975335
558	0.94964	0.978261	0.998684	0.987372	0.976787
559	0.928058	0.978261	0.998684	0.985004	0.972439
560	0.935252	0.978261	0.998684	0.985793	0.973888
561	0.942446	0.978261	0.997368	0.985793	0.973856
562	0.935252	0.975543	1	0.985793	0.97393
563	0.928058	0.980978	0.998684	0.985793	0.973887
564	0.942446	0.980978	1	0.988161	0.978266
565	0.935252	0.972826	0.998684	0.984215	0.970996
566	0.94964	0.975543	1	0.987372	0.976826
567	0.935252	0.983696	0.998684	0.987372	0.976784
568	0.94964	0.980978	1	0.98895	0.979714
569	0.942446	0.975543	1	0.986582	0.975378
570	0.94964	0.978261	0.997368	0.986582	0.975308
571	0.942446	0.980978	0.998684	0.987372	0.976784
572	0.942446	0.978261	0.997368	0.985793	0.973856
573	0.935252	0.980978	1	0.987372	0.976819
574	0.928058	0.980978	0.998684	0.985793	0.973887
575	0.935252	0.975543	0.998684	0.985004	0.972441
576	0.942446	0.980978	0.998684	0.987372	0.976784
577	0.942446	0.978261	0.998684	0.986582	0.975337
578	0.928058	0.978261	1	0.985793	0.973927
579	0.935252	0.975543	0.998684	0.985004	0.972441
580	0.928058	0.980978	0.998684	0.985793	0.973887
581	0.935252	0.980978	0.998684	0.986582	0.975335
582	0.942446	0.980978	1	0.988161	0.978266
583	0.942446	0.975543	0.998684	0.985793	0.973891
584	0.935252	0.975543	1	0.985793	0.97393
585	0.935252	0.978261	1	0.986582	0.975374
586	0.935252	0.980978	0.997368	0.985793	0.973855
587	0.94964	0.978261	0.997368	0.986582	0.975308
588	0.942446	0.975543	0.998684	0.985793	0.973891
589	0.94964	0.975543	0.998684	0.986582	0.975341
590	0.94964	0.975543	1	0.987372	0.976826
591	0.935252	0.980978	0.998684	0.986582	0.975335
592	0.942446	0.978261	0.998684	0.986582	0.975337
593	0.935252	0.978261	0.998684	0.985793	0.973888
594	0.942446	0.980978	0.997368	0.986582	0.975306
595	0.956835	0.980978	0.998684	0.98895	0.979685
596	0.94964	0.978261	0.998684	0.987372	0.976787
597	0.935252	0.975543	0.998684	0.985004	0.972441
598	0.928058	0.980978	0.998684	0.985793	0.973887
599	0.942446	0.978261	0.998684	0.986582	0.975337

600	0.942446	0.975543	1	0.986582	0.975378
601	0.935252	0.978261	0.997368	0.985004	0.972405
602	0.94964	0.980978	0.998684	0.988161	0.978234
603	0.942446	0.983696	1	0.98895	0.979712
604	0.942446	0.975543	0.998684	0.985793	0.973891
605	0.928058	0.980978	0.998684	0.985793	0.973887
606	0.942446	0.978261	0.998684	0.986582	0.975337
607	0.928058	0.978261	1	0.985793	0.973927
608	0.94964	0.978261	1	0.988161	0.97827
609	0.920863	0.975543	0.998684	0.983425	0.969543
610	0.942446	0.983696	0.997368	0.987372	0.976757
611	0.928058	0.980978	1	0.986582	0.975373
612	0.935252	0.983696	0.997368	0.986582	0.975306
613	0.94964	0.978261	0.998684	0.987372	0.976787
614	0.94964	0.972826	0.998684	0.985793	0.973896
615	0.935252	0.978261	0.996053	0.984215	0.970926
616	0.928058	0.978261	0.998684	0.985004	0.972439
617	0.928058	0.975543	0.998684	0.984215	0.970992
618	0.928058	0.983696	0.998684	0.986582	0.975336
619	0.913669	0.980978	0.997368	0.983425	0.969506
620	0.942446	0.980978	0.997368	0.986582	0.975306
621	0.928058	0.975543	0.997368	0.983425	0.969505
622	0.928058	0.972826	0.998684	0.983425	0.969546
623	0.935252	0.978261	0.998684	0.985793	0.973888
624	0.928058	0.978261	0.998684	0.985004	0.972439
625	0.935252	0.978261	0.998684	0.985793	0.973888
626	0.935252	0.980978	0.997368	0.985793	0.973855
627	0.942446	0.980978	1	0.988161	0.978266
628	0.913669	0.983696	0.998684	0.985004	0.972441
629	0.928058	0.978261	0.998684	0.985004	0.972439
630	0.935252	0.975543	0.997368	0.984215	0.970956
631	0.942446	0.978261	0.998684	0.986582	0.975337
632	0.942446	0.980978	0.998684	0.987372	0.976784
633	0.935252	0.980978	0.997368	0.985793	0.973855
634	0.906475	0.980978	0.998684	0.983425	0.969545
635	0.935252	0.980978	0.998684	0.986582	0.975335
636	0.928058	0.975543	0.998684	0.984215	0.970992
637	0.935252	0.972826	0.998684	0.984215	0.970996
638	0.935252	0.972826	0.998684	0.984215	0.970996
639	0.935252	0.980978	0.997368	0.985793	0.973855
640	0.920863	0.978261	0.997368	0.983425	0.969504
641	0.935252	0.970109	0.998684	0.983425	0.969551
642	0.942446	0.975543	0.998684	0.985793	0.973891

643	0.942446	0.978261	0.997368	0.985793	0.973856
644	0.928058	0.978261	1	0.985793	0.973927
645	0.928058	0.980978	0.998684	0.985793	0.973887
646	0.913669	0.978261	0.998684	0.983425	0.969543
647	0.928058	0.975543	0.998684	0.984215	0.970992
648	0.920863	0.978261	0.997368	0.983425	0.969504
649	0.942446	0.980978	0.998684	0.987372	0.976784
650	0.956835	0.980978	0.998684	0.98895	0.979685
651	0.935252	0.978261	0.998684	0.985793	0.973888
652	0.942446	0.983696	0.997368	0.987372	0.976757
653	0.94964	0.980978	0.998684	0.988161	0.978234
654	0.942446	0.978261	0.998684	0.986582	0.975337
655	0.942446	0.978261	1	0.987372	0.976822
656	0.942446	0.980978	0.997368	0.986582	0.975306
657	0.942446	0.975543	0.997368	0.985004	0.972408
658	0.942446	0.983696	0.998684	0.988161	0.978233
659	0.935252	0.978261	0.998684	0.985793	0.973888
660	0.935252	0.978261	0.997368	0.985004	0.972405
661	0.942446	0.975543	0.998684	0.985793	0.973891
662	0.942446	0.978261	0.997368	0.985793	0.973856
663	0.964029	0.975543	0.998684	0.988161	0.978244
664	0.942446	0.980978	0.998684	0.987372	0.976784
665	0.928058	0.975543	0.997368	0.983425	0.969505
666	0.94964	0.975543	0.997368	0.985793	0.97386
667	0.942446	0.975543	0.998684	0.985793	0.973891
668	0.942446	0.975543	0.998684	0.985793	0.973891
669	0.94964	0.975543	0.998684	0.986582	0.975341
670	0.935252	0.978261	1	0.986582	0.975374
671	0.956835	0.975543	1	0.988161	0.978276
672	0.942446	0.978261	0.998684	0.986582	0.975337
673	0.94964	0.975543	1	0.987372	0.976826
674	0.956835	0.975543	1	0.988161	0.978276
675	0.94964	0.972826	0.998684	0.985793	0.973896
676	0.94964	0.975543	1	0.987372	0.976826
677	0.935252	0.972826	0.998684	0.984215	0.970996
678	0.935252	0.978261	0.998684	0.985793	0.973888
679	0.928058	0.978261	0.998684	0.985004	0.972439
680	0.94964	0.978261	0.998684	0.987372	0.976787
681	0.942446	0.983696	0.998684	0.988161	0.978233
682	0.942446	0.978261	0.998684	0.986582	0.975337
683	0.94964	0.978261	0.997368	0.986582	0.975308
684	0.956835	0.978261	0.998684	0.988161	0.978238
685	0.935252	0.978261	0.997368	0.985004	0.972405

686	0.94964	0.980978	0.998684	0.988161	0.978234
687	0.928058	0.980978	1	0.986582	0.975373
688	0.942446	0.975543	0.998684	0.985793	0.973891
689	0.94964	0.978261	0.998684	0.987372	0.976787
690	0.935252	0.978261	1	0.986582	0.975374
691	0.935252	0.980978	0.997368	0.985793	0.973855
692	0.94964	0.970109	0.997368	0.984215	0.970966
693	0.942446	0.978261	0.998684	0.986582	0.975337
694	0.942446	0.972826	0.998684	0.985004	0.972446
695	0.94964	0.975543	1	0.987372	0.976826
696	0.94964	0.975543	1	0.987372	0.976826
697	0.935252	0.980978	1	0.987372	0.976819
698	0.94964	0.978261	0.997368	0.986582	0.975308
699	0.942446	0.980978	0.998684	0.987372	0.976784
700	0.942446	0.970109	0.998684	0.984215	0.971002
701	0.94964	0.972826	0.998684	0.985793	0.973896
702	0.935252	0.978261	0.998684	0.985793	0.973888
703	0.935252	0.975543	0.997368	0.984215	0.970956
704	0.942446	0.980978	0.998684	0.987372	0.976784
705	0.920863	0.980978	0.998684	0.985004	0.972439
706	0.920863	0.978261	0.998684	0.984215	0.970991
707	0.928058	0.978261	0.998684	0.985004	0.972439
708	0.928058	0.978261	0.998684	0.985004	0.972439
709	0.935252	0.983696	1	0.988161	0.978265
710	0.942446	0.978261	0.998684	0.986582	0.975337
711	0.935252	0.978261	0.998684	0.985793	0.973888
712	0.935252	0.980978	1	0.987372	0.976819
713	0.942446	0.972826	0.997368	0.984215	0.97096
714	0.942446	0.978261	0.997368	0.985793	0.973856
715	0.942446	0.978261	0.998684	0.986582	0.975337
716	0.920863	0.975543	0.998684	0.983425	0.969543
717	0.94964	0.978261	0.998684	0.987372	0.976787
718	0.956835	0.978261	0.998684	0.988161	0.978238
719	0.920863	0.978261	0.998684	0.984215	0.970991
720	0.94964	0.975543	1	0.987372	0.976826
721	0.935252	0.975543	0.998684	0.985004	0.972441
722	0.956835	0.975543	0.997368	0.986582	0.975313
723	0.942446	0.980978	0.998684	0.987372	0.976784
724	0.956835	0.972826	0.998684	0.986582	0.975348
725	0.94964	0.978261	0.997368	0.986582	0.975308
726	0.942446	0.978261	0.998684	0.986582	0.975337
727	0.94964	0.975543	0.998684	0.986582	0.975341
728	0.913669	0.978261	0.997368	0.982636	0.968055

729	0.935252	0.975543	1	0.985793	0.97393
730	0.94964	0.975543	1	0.987372	0.976826
731	0.942446	0.978261	0.997368	0.985793	0.973856
732	0.94964	0.972826	0.998684	0.985793	0.973896
733	0.928058	0.978261	0.998684	0.985004	0.972439
734	0.935252	0.980978	0.997368	0.985793	0.973855
735	0.920863	0.972826	0.998684	0.982636	0.968097
736	0.94964	0.975543	0.997368	0.985793	0.97386
737	0.956835	0.975543	0.998684	0.987372	0.976792
738	0.928058	0.975543	0.998684	0.984215	0.970992
739	0.94964	0.975543	0.998684	0.986582	0.975341
740	0.942446	0.983696	0.997368	0.987372	0.976757
741	0.928058	0.978261	0.997368	0.984215	0.970954
742	0.94964	0.978261	0.998684	0.987372	0.976787
743	0.928058	0.975543	1	0.985004	0.972483
744	0.935252	0.980978	0.998684	0.986582	0.975335
745	0.928058	0.978261	0.998684	0.985004	0.972439
746	0.942446	0.983696	1	0.98895	0.979712
747	0.956835	0.972826	0.998684	0.986582	0.975348
748	0.956835	0.972826	0.998684	0.986582	0.975348
749	0.94964	0.975543	1	0.987372	0.976826
750	0.942446	0.975543	1	0.986582	0.975378
751	0.935252	0.983696	1	0.988161	0.978265
752	0.942446	0.975543	0.998684	0.985793	0.973891
753	0.928058	0.975543	0.998684	0.984215	0.970992
754	0.935252	0.978261	0.998684	0.985793	0.973888
755	0.956835	0.983696	0.997368	0.98895	0.979661
756	0.942446	0.975543	0.998684	0.985793	0.973891
757	0.942446	0.978261	0.998684	0.986582	0.975337
758	0.942446	0.975543	0.998684	0.985793	0.973891
759	0.920863	0.972826	0.998684	0.982636	0.968097
760	0.928058	0.975543	0.998684	0.984215	0.970992
761	0.94964	0.972826	0.998684	0.985793	0.973896
762	0.942446	0.972826	0.998684	0.985004	0.972446
763	0.94964	0.978261	0.998684	0.987372	0.976787
764	0.942446	0.980978	1	0.988161	0.978266
765	0.942446	0.975543	0.998684	0.985793	0.973891
766	0.956835	0.978261	0.997368	0.987372	0.976761
767	0.920863	0.975543	0.997368	0.982636	0.968054
768	0.956835	0.972826	0.997368	0.985793	0.973866
769	0.956835	0.972826	0.998684	0.986582	0.975348
770	0.935252	0.972826	0.998684	0.984215	0.970996
771	0.942446	0.975543	0.998684	0.985793	0.973891

772	0.942446	0.975543	0.998684	0.985793	0.973891
773	0.94964	0.970109	0.997368	0.984215	0.970966
774	0.94964	0.972826	0.997368	0.985004	0.972413
775	0.94964	0.978261	0.997368	0.986582	0.975308
776	0.942446	0.978261	1	0.987372	0.976822
777	0.956835	0.980978	0.997368	0.988161	0.97821
778	0.94964	0.980978	0.997368	0.987372	0.976758
779	0.942446	0.978261	0.998684	0.986582	0.975337
780	0.935252	0.978261	0.997368	0.985004	0.972405
781	0.928058	0.975543	0.998684	0.984215	0.970992
782	0.942446	0.972826	0.998684	0.985004	0.972446
783	0.935252	0.978261	0.998684	0.985793	0.973888
784	0.94964	0.975543	1	0.987372	0.976826
785	0.956835	0.978261	1	0.98895	0.979719
786	0.94964	0.978261	0.998684	0.987372	0.976787
787	0.935252	0.970109	0.997368	0.982636	0.968061
788	0.942446	0.975543	0.998684	0.985793	0.973891
789	0.956835	0.975543	0.998684	0.987372	0.976792
790	0.942446	0.978261	0.998684	0.986582	0.975337
791	0.94964	0.972826	0.998684	0.985793	0.973896
792	0.942446	0.980978	0.998684	0.987372	0.976784
793	0.928058	0.975543	0.998684	0.984215	0.970992
794	0.942446	0.980978	0.998684	0.987372	0.976784
795	0.942446	0.978261	0.998684	0.986582	0.975337
796	0.942446	0.972826	0.997368	0.984215	0.97096
797	0.956835	0.975543	1	0.988161	0.978276
798	0.94964	0.978261	0.998684	0.987372	0.976787
799	0.935252	0.975543	0.998684	0.985004	0.972441
800	0.94964	0.975543	1	0.987372	0.976826
801	0.94964	0.972826	1	0.986582	0.975384
802	0.935252	0.978261	0.998684	0.985793	0.973888
803	0.935252	0.978261	0.997368	0.985004	0.972405
804	0.935252	0.975543	0.998684	0.985004	0.972441
805	0.942446	0.975543	1	0.986582	0.975378
806	0.956835	0.975543	0.998684	0.987372	0.976792
807	0.935252	0.980978	0.998684	0.986582	0.975335
808	0.94964	0.975543	0.997368	0.985793	0.97386
809	0.942446	0.978261	0.998684	0.986582	0.975337
810	0.956835	0.972826	0.997368	0.985793	0.973866
811	0.94964	0.978261	0.997368	0.986582	0.975308
812	0.942446	0.983696	0.998684	0.988161	0.978233
813	0.935252	0.978261	0.998684	0.985793	0.973888
814	0.935252	0.972826	1	0.985004	0.972487

815	0.942446	0.975543	0.997368	0.985004	0.972408
816	0.956835	0.978261	0.997368	0.987372	0.976761
817	0.928058	0.972826	0.997368	0.982636	0.968057
818	0.94964	0.980978	0.997368	0.987372	0.976758
819	0.94964	0.975543	0.997368	0.985793	0.97386
820	0.935252	0.978261	0.997368	0.985004	0.972405
821	0.94964	0.978261	0.998684	0.987372	0.976787
822	0.942446	0.975543	0.998684	0.985793	0.973891
823	0.956835	0.978261	0.998684	0.988161	0.978238
824	0.956835	0.978261	0.998684	0.988161	0.978238
825	0.942446	0.978261	0.998684	0.986582	0.975337
826	0.942446	0.978261	0.998684	0.986582	0.975337
827	0.942446	0.980978	1	0.988161	0.978266
828	0.94964	0.975543	0.997368	0.985793	0.97386
829	0.94964	0.975543	0.998684	0.986582	0.975341
830	0.956835	0.975543	0.998684	0.987372	0.976792
831	0.956835	0.980978	0.997368	0.988161	0.97821
832	0.956835	0.975543	0.998684	0.987372	0.976792
833	0.942446	0.972826	0.997368	0.984215	0.97096
834	0.942446	0.980978	0.998684	0.987372	0.976784
835	0.920863	0.975543	1	0.984215	0.971036
836	0.942446	0.978261	0.998684	0.986582	0.975337
837	0.920863	0.975543	0.998684	0.983425	0.969543
838	0.942446	0.978261	0.997368	0.985793	0.973856
839	0.956835	0.975543	0.998684	0.987372	0.976792
840	0.94964	0.972826	1	0.986582	0.975384
841	0.956835	0.972826	0.998684	0.986582	0.975348
842	0.956835	0.972826	0.997368	0.985793	0.973866
843	0.94964	0.975543	0.997368	0.985793	0.97386
844	0.964029	0.980978	0.998684	0.98974	0.981136
845	0.956835	0.975543	0.997368	0.986582	0.975313
846	0.94964	0.978261	0.998684	0.987372	0.976787
847	0.942446	0.978261	0.998684	0.986582	0.975337
848	0.94964	0.978261	0.997368	0.986582	0.975308
849	0.935252	0.975543	0.998684	0.985004	0.972441
850	0.942446	0.975543	0.998684	0.985793	0.973891
851	0.942446	0.975543	0.998684	0.985793	0.973891
852	0.956835	0.975543	0.997368	0.986582	0.975313
853	0.956835	0.972826	0.997368	0.985793	0.973866
854	0.956835	0.978261	1	0.98895	0.979719
855	0.956835	0.972826	1	0.987372	0.976834
856	0.935252	0.975543	0.998684	0.985004	0.972441
857	0.942446	0.978261	0.998684	0.986582	0.975337

858	0.956835	0.975543	0.998684	0.987372	0.976792
859	0.956835	0.978261	0.998684	0.988161	0.978238
860	0.956835	0.978261	0.998684	0.988161	0.978238
861	0.94964	0.980978	0.998684	0.988161	0.978234
862	0.94964	0.978261	0.997368	0.986582	0.975308
863	0.942446	0.970109	0.997368	0.983425	0.969513
864	0.956835	0.975543	0.998684	0.987372	0.976792
865	0.942446	0.975543	1	0.986582	0.975378
866	0.956835	0.970109	0.998684	0.985793	0.973904
867	0.956835	0.978261	0.998684	0.988161	0.978238
868	0.956835	0.972826	0.998684	0.986582	0.975348
869	0.94964	0.967391	0.998684	0.984215	0.97101
870	0.956835	0.975543	0.997368	0.986582	0.975313
871	0.94964	0.975543	0.998684	0.986582	0.975341
872	0.94964	0.980978	0.998684	0.988161	0.978234
873	0.956835	0.983696	0.998684	0.98974	0.981133
874	0.942446	0.972826	1	0.985793	0.973935
875	0.956835	0.978261	0.998684	0.988161	0.978238
876	0.94964	0.975543	0.998684	0.986582	0.975341
877	0.942446	0.972826	0.998684	0.985004	0.972446
878	0.94964	0.978261	0.997368	0.986582	0.975308
879	0.94964	0.980978	0.998684	0.988161	0.978234
880	0.942446	0.975543	0.997368	0.985004	0.972408
881	0.94964	0.975543	0.998684	0.986582	0.975341
882	0.94964	0.975543	1	0.987372	0.976826
883	0.935252	0.980978	0.997368	0.985793	0.973855
884	0.956835	0.975543	0.998684	0.987372	0.976792
885	0.956835	0.978261	0.997368	0.987372	0.976761
886	0.964029	0.972826	0.998684	0.987372	0.9768
887	0.935252	0.975543	0.998684	0.985004	0.972441
888	0.956835	0.978261	0.997368	0.987372	0.976761
889	0.956835	0.972826	0.998684	0.986582	0.975348
890	0.942446	0.978261	0.997368	0.985793	0.973856
891	0.942446	0.978261	0.998684	0.986582	0.975337
892	0.935252	0.978261	0.998684	0.985793	0.973888
893	0.94964	0.978261	0.998684	0.987372	0.976787
894	0.942446	0.978261	0.998684	0.986582	0.975337
895	0.94964	0.975543	0.998684	0.986582	0.975341
896	0.942446	0.975543	0.998684	0.985793	0.973891
897	0.942446	0.975543	0.998684	0.985793	0.973891
898	0.942446	0.978261	0.998684	0.986582	0.975337
899	0.94964	0.975543	1	0.987372	0.976826
900	0.956835	0.978261	0.997368	0.987372	0.976761

901	0.928058	0.975543	0.998684	0.984215	0.970992
902	0.94964	0.972826	0.998684	0.985793	0.973896
903	0.942446	0.972826	0.998684	0.985004	0.972446
904	0.94964	0.978261	0.997368	0.986582	0.975308
905	0.94964	0.980978	0.998684	0.988161	0.978234
906	0.956835	0.975543	0.998684	0.987372	0.976792
907	0.94964	0.978261	0.998684	0.987372	0.976787
908	0.956835	0.978261	0.997368	0.987372	0.976761
909	0.956835	0.975543	0.998684	0.987372	0.976792
910	0.94964	0.978261	0.998684	0.987372	0.976787
911	0.94964	0.978261	0.997368	0.986582	0.975308
912	0.956835	0.972826	1	0.987372	0.976834
913	0.94964	0.978261	0.998684	0.987372	0.976787
914	0.956835	0.972826	0.998684	0.986582	0.975348
915	0.94964	0.975543	0.998684	0.986582	0.975341
916	0.935252	0.975543	1	0.985793	0.97393
917	0.94964	0.972826	1	0.986582	0.975384
918	0.94964	0.978261	0.997368	0.986582	0.975308
919	0.956835	0.978261	0.998684	0.988161	0.978238
920	0.94964	0.972826	0.998684	0.985793	0.973896
921	0.94964	0.978261	0.997368	0.986582	0.975308
922	0.942446	0.972826	0.998684	0.985004	0.972446
923	0.956835	0.978261	0.998684	0.988161	0.978238
924	0.942446	0.978261	0.998684	0.986582	0.975337
925	0.94964	0.975543	0.998684	0.986582	0.975341
926	0.964029	0.972826	0.997368	0.986582	0.97532
927	0.956835	0.975543	0.997368	0.986582	0.975313
928	0.94964	0.972826	1	0.986582	0.975384
929	0.942446	0.978261	0.998684	0.986582	0.975337
930	0.935252	0.978261	0.998684	0.985793	0.973888
931	0.956835	0.978261	0.998684	0.988161	0.978238
932	0.935252	0.975543	0.998684	0.985004	0.972441
933	0.94964	0.978261	0.997368	0.986582	0.975308
934	0.94964	0.972826	0.998684	0.985793	0.973896
935	0.94964	0.978261	0.997368	0.986582	0.975308
936	0.94964	0.975543	0.997368	0.985793	0.97386
937	0.956835	0.975543	0.998684	0.987372	0.976792
938	0.94964	0.972826	0.998684	0.985793	0.973896
939	0.94964	0.975543	0.998684	0.986582	0.975341
940	0.935252	0.978261	0.998684	0.985793	0.973888
941	0.94964	0.975543	0.997368	0.985793	0.97386
942	0.94964	0.978261	0.998684	0.987372	0.976787
943	0.94964	0.978261	0.998684	0.987372	0.976787

944	0.94964	0.980978	1	0.98895	0.979714
945	0.956835	0.978261	0.998684	0.988161	0.978238
946	0.942446	0.978261	0.998684	0.986582	0.975337
947	0.94964	0.978261	0.997368	0.986582	0.975308
948	0.942446	0.978261	0.997368	0.985793	0.973856
949	0.956835	0.970109	0.998684	0.985793	0.973904
950	0.942446	0.972826	0.997368	0.984215	0.97096
951	0.942446	0.975543	0.997368	0.985004	0.972408
952	0.956835	0.978261	1	0.98895	0.979719
953	0.942446	0.978261	0.998684	0.986582	0.975337
954	0.94964	0.975543	0.998684	0.986582	0.975341
955	0.94964	0.975543	1	0.987372	0.976826
956	0.964029	0.978261	1	0.98974	0.981168
957	0.956835	0.978261	0.998684	0.988161	0.978238
958	0.935252	0.978261	0.997368	0.985004	0.972405
959	0.942446	0.980978	0.998684	0.987372	0.976784
960	0.94964	0.978261	0.997368	0.986582	0.975308
961	0.964029	0.980978	0.997368	0.98895	0.979664
962	0.956835	0.978261	0.998684	0.988161	0.978238
963	0.94964	0.978261	0.998684	0.987372	0.976787
964	0.956835	0.972826	0.998684	0.986582	0.975348
965	0.942446	0.972826	1	0.985793	0.973935
966	0.935252	0.978261	0.998684	0.985793	0.973888
967	0.94964	0.978261	0.997368	0.986582	0.975308
968	0.94964	0.975543	0.998684	0.986582	0.975341
969	0.94964	0.978261	0.997368	0.986582	0.975308
970	0.942446	0.970109	0.998684	0.984215	0.971002
971	0.964029	0.975543	0.998684	0.988161	0.978244
972	0.942446	0.975543	0.998684	0.985793	0.973891
973	0.94964	0.972826	0.998684	0.985793	0.973896
974	0.94964	0.975543	0.997368	0.985793	0.97386
975	0.94964	0.978261	0.998684	0.987372	0.976787
976	0.964029	0.975543	1	0.98895	0.979725
977	0.942446	0.975543	0.997368	0.985004	0.972408
978	0.94964	0.975543	0.998684	0.986582	0.975341
979	0.94964	0.978261	1	0.988161	0.97827
980	0.942446	0.980978	0.998684	0.987372	0.976784
981	0.94964	0.975543	0.997368	0.985793	0.97386
982	0.956835	0.978261	0.998684	0.988161	0.978238
983	0.942446	0.980978	0.997368	0.986582	0.975306
984	0.964029	0.975543	0.998684	0.988161	0.978244
985	0.964029	0.978261	0.998684	0.98895	0.979689
986	0.956835	0.975543	0.997368	0.986582	0.975313

987	0.942446	0.975543	0.998684	0.985793	0.973891
988	0.942446	0.975543	0.997368	0.985004	0.972408
989	0.942446	0.978261	0.997368	0.985793	0.973856
990	0.942446	0.978261	0.998684	0.986582	0.975337
991	0.935252	0.978261	0.997368	0.985004	0.972405
992	0.942446	0.980978	0.998684	0.987372	0.976784
993	0.956835	0.975543	0.998684	0.987372	0.976792
994	0.935252	0.975543	1	0.985793	0.97393
995	0.942446	0.978261	0.998684	0.986582	0.975337
996	0.956835	0.978261	0.998684	0.988161	0.978238
997	0.94964	0.978261	0.998684	0.987372	0.976787
998	0.956835	0.975543	0.998684	0.987372	0.976792
999	0.956835	0.978261	1	0.98895	0.979719
1000	0.956835	0.978261	0.998684	0.988161	0.978238

Table S6. The accuracies for different races and total prediction accuracy obtained by IFS method and random forest

Number of features	Accuracy for American race	Accuracy for Asian race	Accuracy for European race	Overall prediction accuracy	MCC
4	0.956835	0.869565	0.942105	0.922652	0.857483
5	0.992806	0.869565	0.95	0.931334	0.873473
6	0.985612	0.904891	0.955263	0.943962	0.897008
7	0.985612	0.910326	0.957895	0.947119	0.90274
8	0.978417	0.921196	0.957895	0.949487	0.907153
9	0.978417	0.92663	0.971053	0.958958	0.924256
10	0.978417	0.929348	0.965789	0.95659	0.920052
11	0.985612	0.934783	0.973684	0.963694	0.933121
12	0.978417	0.961957	0.973684	0.970797	0.946363
13	0.978417	0.951087	0.972368	0.966851	0.939069
14	0.978417	0.964674	0.977632	0.973954	0.952116
15	0.978417	0.967391	0.975	0.973165	0.950752
16	0.985612	0.964674	0.981579	0.977111	0.957891
17	0.978417	0.967391	0.986842	0.980268	0.96363
18	0.971223	0.967391	0.982895	0.977111	0.957855
19	0.978417	0.967391	0.985526	0.979479	0.962191
20	0.985612	0.975543	0.980263	0.979479	0.962345
21	0.985612	0.967391	0.982895	0.97869	0.960785
22	0.978417	0.956522	0.980263	0.973165	0.950566
23	0.971223	0.970109	0.985526	0.979479	0.962191
24	0.985612	0.964674	0.989474	0.981847	0.966545
25	0.978417	0.964674	0.984211	0.977901	0.959293
26	0.985612	0.967391	0.984211	0.979479	0.962223
27	0.978417	0.953804	0.986842	0.976322	0.956343
28	0.978417	0.970109	0.992105	0.984215	0.970918
29	0.985612	0.967391	0.982895	0.97869	0.960823
30	0.985612	0.964674	0.990789	0.982636	0.968032
31	0.985612	0.967391	0.988158	0.981847	0.96656
32	0.985612	0.970109	0.992105	0.985004	0.972372
33	0.985612	0.972826	0.988158	0.983425	0.96948
34	0.978417	0.961957	0.985526	0.977901	0.959278
35	0.985612	0.970109	0.984211	0.980268	0.96369
36	0.985612	0.980978	0.986842	0.985004	0.972437
37	0.978417	0.98913	0.990789	0.98895	0.979675
38	0.971223	0.997283	0.992105	0.991318	0.984041
39	0.985612	0.994565	0.992105	0.992107	0.985503
40	0.978417	0.983696	0.989474	0.986582	0.97531

41	0.978417	0.986413	0.992105	0.98895	0.979655
42	0.971223	0.98913	0.990789	0.988161	0.978211
43	0.985612	0.986413	0.990789	0.98895	0.979676
44	0.978417	0.98913	0.986842	0.986582	0.975368
45	0.978417	0.98913	0.992105	0.98974	0.981128
46	0.964029	0.98913	0.988158	0.985793	0.973869
47	0.978417	0.986413	0.993421	0.98974	0.981104
48	0.985612	0.986413	0.988158	0.987372	0.976808
49	0.971223	0.972826	0.990789	0.983425	0.969449
50	0.978417	0.980978	0.986842	0.984215	0.970959
51	0.978417	0.980978	0.992105	0.987372	0.976735
52	0.978417	0.991848	0.990789	0.98974	0.981152
53	0.964029	0.986413	0.992105	0.987372	0.976733
54	0.971223	0.986413	0.996053	0.990529	0.982559
55	0.978417	0.980978	0.994737	0.98895	0.979651
56	0.978417	0.986413	0.993421	0.98974	0.981104
57	0.978417	0.978261	0.996053	0.98895	0.979656
58	0.978417	0.975543	0.990789	0.985004	0.97238
59	0.964029	0.980978	0.994737	0.987372	0.97673
60	0.978417	0.986413	0.988158	0.986582	0.975349
61	0.964029	0.991848	0.993421	0.98974	0.981104
62	0.978417	0.983696	0.990789	0.987372	0.97675
63	0.978417	0.994565	0.993421	0.992107	0.985493
64	0.978417	0.991848	0.992105	0.990529	0.982579
65	0.978417	0.98913	0.994737	0.991318	0.984014
66	0.978417	0.986413	0.992105	0.98895	0.979661
67	0.985612	0.975543	0.990789	0.985793	0.97383
68	0.971223	0.98913	0.993421	0.98974	0.981104
69	0.985612	0.98913	0.994737	0.992107	0.985471
70	0.978417	0.983696	0.989474	0.986582	0.975321
71	0.978417	0.980978	0.990789	0.986582	0.975295
72	0.978417	0.98913	0.994737	0.991318	0.984014
73	0.964029	0.98913	0.993421	0.98895	0.979649
74	0.978417	0.983696	0.990789	0.987372	0.976754
75	0.978417	0.980978	0.990789	0.986582	0.975289
76	0.978417	0.986413	0.993421	0.98974	0.981104
77	0.985612	0.986413	0.993421	0.990529	0.982564
78	0.971223	0.994565	0.996053	0.992897	0.986924
79	0.978417	0.994565	0.994737	0.992897	0.98693
80	0.971223	0.98913	0.992105	0.98895	0.979665
81	0.971223	0.980978	0.988158	0.984215	0.970936
82	0.971223	0.986413	0.990789	0.987372	0.97675
83	0.971223	0.986413	0.992105	0.988161	0.978194

84	0.964029	0.983696	0.990789	0.985793	0.973821
85	0.971223	0.98913	0.993421	0.98974	0.981104
86	0.985612	0.975543	0.994737	0.988161	0.978209
87	0.978417	0.98913	0.992105	0.98974	0.981117
88	0.978417	0.991848	0.992105	0.990529	0.982579
89	0.956835	0.991848	0.994737	0.98974	0.981098
90	0.985612	0.980978	0.993421	0.98895	0.979664
91	0.978417	0.986413	0.989474	0.987372	0.97677
92	0.971223	0.983696	0.993421	0.988161	0.978189
93	0.971223	0.980978	0.993421	0.987372	0.976739
94	0.978417	0.986413	0.996053	0.991318	0.984015
95	0.978417	0.98913	0.993421	0.990529	0.982564
96	0.978417	0.983696	0.989474	0.986582	0.97531
97	0.978417	0.98913	0.994737	0.991318	0.984013
98	0.992806	0.983696	0.993421	0.990529	0.982572
99	0.971223	0.98913	0.993421	0.98974	0.981104
100	0.978417	0.991848	0.997368	0.993686	0.988378
101	0.971223	0.983696	0.997368	0.990529	0.982567
102	0.971223	0.980978	0.996053	0.98895	0.97965
103	0.956835	0.986413	0.998684	0.990529	0.982584
104	0.964029	0.986413	0.994737	0.98895	0.979642
105	0.971223	0.991848	0.994737	0.991318	0.984013
106	0.956835	0.983696	0.993421	0.986582	0.975269
107	0.985612	0.978261	0.993421	0.988161	0.978209
108	0.971223	0.986413	0.992105	0.988161	0.978198
109	0.971223	0.98913	0.993421	0.98974	0.98111
110	0.971223	0.98913	0.994737	0.990529	0.982556
111	0.964029	0.983696	0.990789	0.985793	0.973822
112	0.971223	0.98913	0.993421	0.98974	0.981104
113	0.964029	0.98913	0.993421	0.98895	0.979645
114	0.978417	0.991848	0.994737	0.992107	0.985478
115	0.964029	0.98913	0.989474	0.986582	0.975306
116	0.971223	0.98913	0.992105	0.98895	0.979656
117	0.978417	0.986413	0.993421	0.98974	0.981104
118	0.971223	0.986413	0.994737	0.98974	0.981098
119	0.971223	0.98913	0.993421	0.98974	0.981104
120	0.978417	0.983696	0.996053	0.990529	0.982559
121	0.971223	0.991848	0.994737	0.991318	0.984017
122	0.964029	0.978261	0.996053	0.987372	0.976743
123	0.964029	0.991848	0.993421	0.98974	0.981106
124	0.978417	0.983696	0.990789	0.987372	0.97675
125	0.964029	0.994565	0.990789	0.98895	0.979675
126	0.971223	0.991848	0.993421	0.990529	0.982564

127	0.971223	0.994565	0.993421	0.991318	0.984023
128	0.971223	0.986413	0.996053	0.990529	0.982556
129	0.971223	0.991848	0.996053	0.992107	0.985467
130	0.971223	0.980978	0.994737	0.988161	0.978188
131	0.956835	0.983696	0.990789	0.985004	0.972359
132	0.964029	0.98913	0.994737	0.98974	0.981099
133	0.978417	0.98913	0.996053	0.992107	0.985466
134	0.985612	0.98913	0.996053	0.992897	0.986922
135	0.971223	0.991848	0.994737	0.991318	0.984014
136	0.964029	0.986413	0.990789	0.986582	0.975284
137	0.964029	0.986413	0.993421	0.988161	0.978186
138	0.964029	0.994565	0.996053	0.992107	0.985469
139	0.94964	0.994565	0.990789	0.987372	0.976747
140	0.942446	0.983696	0.993421	0.985004	0.972353
141	0.964029	0.991848	0.993421	0.98974	0.981106
142	0.964029	0.983696	0.990789	0.985793	0.97383
143	0.978417	0.983696	0.996053	0.990529	0.982564
144	0.964029	0.994565	0.990789	0.98895	0.979683
145	0.978417	0.994565	0.992105	0.991318	0.984041
146	0.956835	0.983696	0.994737	0.987372	0.976734
147	0.971223	0.98913	0.994737	0.990529	0.982556
148	0.978417	0.991848	0.994737	0.992107	0.985471
149	0.978417	0.98913	0.992105	0.98974	0.981117
150	0.964029	0.98913	0.986842	0.985004	0.972428
151	0.94964	0.978261	0.989474	0.981847	0.966527
152	0.964029	0.983696	0.996053	0.98895	0.979648
153	0.971223	0.986413	0.997368	0.991318	0.984018
154	0.971223	0.994565	0.994737	0.992107	0.985473
155	0.971223	0.980978	0.993421	0.987372	0.976733
156	0.978417	0.994565	0.993421	0.992107	0.985483
157	0.94964	0.98913	0.996053	0.98895	0.979649
158	0.978417	0.991848	0.990789	0.98974	0.98114
159	0.964029	0.98913	0.993421	0.98895	0.979645
160	0.978417	0.991848	0.992105	0.990529	0.982579
161	0.978417	0.986413	0.994737	0.990529	0.982556
162	0.971223	0.991848	0.990789	0.98895	0.979675
163	0.964029	0.98913	0.997368	0.991318	0.984017
164	0.971223	0.986413	0.992105	0.988161	0.978198
165	0.964029	0.986413	0.992105	0.987372	0.976735
166	0.978417	0.986413	0.993421	0.98974	0.981126
167	0.964029	0.98913	0.990789	0.987372	0.976747
168	0.978417	0.986413	0.994737	0.990529	0.982556
169	0.956835	0.980978	0.993421	0.985793	0.973813

170	0.956835	0.98913	0.992105	0.987372	0.976739
171	0.956835	0.978261	0.994737	0.985793	0.973823
172	0.964029	0.991848	0.993421	0.98974	0.981104
173	0.964029	0.994565	0.992105	0.98974	0.981119
174	0.956835	0.986413	0.994737	0.988161	0.978185
175	0.978417	0.98913	0.993421	0.990529	0.982572
176	0.978417	0.983696	0.990789	0.987372	0.976776
177	0.978417	0.994565	0.992105	0.991318	0.984041
178	0.971223	0.98913	0.994737	0.990529	0.982555
179	0.971223	0.986413	0.990789	0.987372	0.976747
180	0.978417	0.994565	0.997368	0.994475	0.989831
181	0.971223	0.986413	0.993421	0.98895	0.979646
182	0.971223	0.991848	0.992105	0.98974	0.981127
183	0.978417	0.98913	0.990789	0.98895	0.979676
184	0.971223	0.986413	0.990789	0.987372	0.976747
185	0.964029	0.991848	0.993421	0.98974	0.981104
186	0.964029	0.986413	0.993421	0.988161	0.978186
187	0.964029	0.991848	0.994737	0.990529	0.982559
188	0.971223	0.98913	0.989474	0.987372	0.97677
189	0.971223	0.994565	0.992105	0.990529	0.98258
190	0.956835	0.994565	0.992105	0.98895	0.979659
191	0.964029	0.98913	0.993421	0.98895	0.979645
192	0.942446	0.991848	0.994737	0.988161	0.978192
193	0.956835	0.994565	0.994737	0.990529	0.982562
194	0.978417	0.991848	0.993421	0.991318	0.984023
195	0.956835	0.986413	0.996053	0.98895	0.979647
196	0.978417	0.980978	0.989474	0.985793	0.973848
197	0.942446	0.991848	0.992105	0.986582	0.97528
198	0.956835	0.986413	0.992105	0.986582	0.975273
199	0.971223	0.994565	0.993421	0.991318	0.984026
200	0.971223	0.994565	0.989474	0.98895	0.979701
201	0.964029	0.991848	0.990789	0.988161	0.978211
202	0.971223	0.98913	0.992105	0.98895	0.979655
203	0.964029	0.98913	0.994737	0.98974	0.9811
204	0.971223	0.975543	0.994737	0.986582	0.975289
205	0.956835	0.991848	0.990789	0.987372	0.97675
206	0.971223	0.986413	0.990789	0.987372	0.976768
207	0.978417	0.98913	0.994737	0.991318	0.984013
208	0.956835	0.983696	0.993421	0.986582	0.975272
209	0.964029	0.991848	0.992105	0.98895	0.979655
210	0.964029	0.983696	0.993421	0.987372	0.976733
211	0.964029	0.991848	0.996053	0.991318	0.984011
212	0.964029	0.98913	0.992105	0.988161	0.978194

213	0.964029	0.997283	0.993421	0.991318	0.984023
214	0.971223	0.994565	0.993421	0.991318	0.984023
215	0.971223	0.98913	0.993421	0.98974	0.981106
216	0.956835	0.994565	0.996053	0.991318	0.984012
217	0.978417	0.983696	0.990789	0.987372	0.976754
218	0.978417	0.98913	0.990789	0.98895	0.979674
219	0.964029	0.991848	0.993421	0.98974	0.981104
220	0.978417	0.98913	0.982895	0.984215	0.971093
221	0.956835	0.98913	0.993421	0.988161	0.978186
222	0.956835	0.986413	0.994737	0.988161	0.978185
223	0.956835	0.991848	0.996053	0.990529	0.982559
224	0.964029	0.98913	0.993421	0.98895	0.979646
225	0.94964	0.991848	0.994737	0.98895	0.979642
226	0.971223	0.991848	0.988158	0.987372	0.976801
227	0.971223	0.991848	0.990789	0.98895	0.979676
228	0.978417	0.980978	0.990789	0.986582	0.975289
229	0.985612	0.980978	0.990789	0.987372	0.976761
230	0.956835	0.986413	0.993421	0.987372	0.976733
231	0.964029	0.991848	0.994737	0.990529	0.982557
232	0.964029	0.98913	0.993421	0.98895	0.979645
233	0.956835	0.98913	0.989474	0.985793	0.97384
234	0.956835	0.991848	0.994737	0.98974	0.981102
235	0.94964	0.98913	0.992105	0.986582	0.975273
236	0.964029	0.994565	0.994737	0.991318	0.984017
237	0.971223	0.983696	0.997368	0.990529	0.982567
238	0.956835	0.98913	0.990789	0.986582	0.975284
239	0.956835	0.983696	0.996053	0.988161	0.978193
240	0.971223	0.994565	0.996053	0.992897	0.986924
241	0.94964	0.978261	0.994737	0.985004	0.972363
242	0.956835	0.980978	0.990789	0.984215	0.970901
243	0.978417	0.994565	0.993421	0.992107	0.985485
244	0.971223	0.986413	0.994737	0.98974	0.981098
245	0.971223	0.986413	0.993421	0.98895	0.979646
246	0.978417	0.98913	0.996053	0.992107	0.985466
247	0.956835	0.991848	0.994737	0.98974	0.981102
248	0.971223	0.978261	0.992105	0.985793	0.97382
249	0.964029	0.98913	0.994737	0.98974	0.981099
250	0.956835	0.98913	0.992105	0.987372	0.976733
251	0.971223	0.991848	0.992105	0.98974	0.981117
252	0.956835	0.991848	0.994737	0.98974	0.981099
253	0.942446	0.994565	0.997368	0.990529	0.982572
254	0.956835	0.991848	0.992105	0.988161	0.978202
255	0.971223	0.98913	0.993421	0.98974	0.981104

256	0.964029	0.98913	0.997368	0.991318	0.984017
257	0.978417	0.997283	0.992105	0.992107	0.98552
258	0.956835	0.994565	0.992105	0.98895	0.979656
259	0.971223	0.991848	0.993421	0.990529	0.982567
260	0.964029	0.994565	0.992105	0.98974	0.981117
261	0.971223	0.980978	0.996053	0.98895	0.979651
262	0.94964	0.991848	0.994737	0.98895	0.979643
263	0.94964	0.986413	0.990789	0.985004	0.972359
264	0.942446	0.986413	0.992105	0.985004	0.972353
265	0.971223	0.986413	0.992105	0.988161	0.978194
266	0.964029	0.980978	0.993421	0.986582	0.975269
267	0.971223	0.991848	0.990789	0.98895	0.979676
268	0.956835	0.994565	0.993421	0.98974	0.981111
269	0.964029	0.983696	0.994737	0.988161	0.978185
270	0.935252	0.98913	0.989474	0.983425	0.969447
271	0.964029	0.991848	0.993421	0.98974	0.981106
272	0.94964	0.991848	0.994737	0.98895	0.979645
273	0.956835	0.983696	0.994737	0.987372	0.976727
274	0.964029	0.994565	0.993421	0.990529	0.982565
275	0.971223	0.991848	0.996053	0.992107	0.985467
276	0.94964	0.98913	0.994737	0.988161	0.978189
277	0.964029	0.994565	0.994737	0.991318	0.984014
278	0.94964	0.994565	0.994737	0.98974	0.981107
279	0.935252	0.991848	0.993421	0.986582	0.97527
280	0.942446	0.991848	0.996053	0.98895	0.979651
281	0.956835	0.98913	0.990789	0.986582	0.975293
282	0.94964	0.991848	0.993421	0.988161	0.978186
283	0.956835	0.983696	0.996053	0.988161	0.978193
284	0.956835	0.991848	0.993421	0.98895	0.979645
285	0.964029	0.983696	0.993421	0.987372	0.976729
286	0.978417	0.980978	0.990789	0.986582	0.975289
287	0.964029	0.98913	0.993421	0.98895	0.979644
288	0.971223	0.980978	0.990789	0.985793	0.973825
289	0.978417	0.991848	0.993421	0.991318	0.984023
290	0.964029	0.98913	0.992105	0.988161	0.978195
291	0.942446	0.991848	0.996053	0.98895	0.979651
292	0.94964	0.986413	0.994737	0.987372	0.97673
293	0.978417	0.98913	0.994737	0.991318	0.98402
294	0.971223	0.980978	0.989474	0.985004	0.972377
295	0.964029	0.98913	0.996053	0.990529	0.982556
296	0.971223	0.991848	0.992105	0.98974	0.981117
297	0.964029	0.991848	0.993421	0.98974	0.981104
298	0.978417	0.991848	0.989474	0.98895	0.979703

299	0.971223	0.994565	0.993421	0.991318	0.984026
300	0.956835	0.986413	0.993421	0.987372	0.976727
301	0.956835	0.991848	0.992105	0.988161	0.978196
302	0.971223	0.98913	0.994737	0.990529	0.982558
303	0.964029	0.997283	0.992105	0.990529	0.98258
304	0.94964	0.986413	0.993421	0.986582	0.97527
305	0.964029	0.986413	0.993421	0.988161	0.978186
306	0.964029	0.98913	0.993421	0.98895	0.979649
307	0.971223	0.98913	0.993421	0.98974	0.981104
308	0.971223	0.991848	0.994737	0.991318	0.984013
309	0.964029	0.98913	0.993421	0.98895	0.979644
310	0.971223	0.98913	0.996053	0.991318	0.984012
311	0.94964	0.986413	0.994737	0.987372	0.97673
312	0.964029	0.98913	0.994737	0.98974	0.981099
313	0.956835	0.994565	0.990789	0.988161	0.978212
314	0.94964	0.994565	0.993421	0.98895	0.979655
315	0.971223	0.98913	0.990789	0.988161	0.978224
316	0.94964	0.986413	0.993421	0.986582	0.975269
317	0.956835	0.991848	0.993421	0.98895	0.979649
318	0.964029	0.98913	0.996053	0.990529	0.982556
319	0.956835	0.997283	0.993421	0.990529	0.982575
320	0.935252	0.98913	0.997368	0.988161	0.978205
321	0.964029	0.983696	0.993421	0.987372	0.976729
322	0.971223	0.98913	0.996053	0.991318	0.984011
323	0.964029	0.997283	0.993421	0.991318	0.984026
324	0.964029	0.997283	0.992105	0.990529	0.982583
325	0.94964	0.986413	0.992105	0.985793	0.973812
326	0.935252	0.986413	0.990789	0.983425	0.969435
327	0.978417	0.994565	0.993421	0.992107	0.985493
328	0.964029	0.994565	0.994737	0.991318	0.984019
329	0.971223	0.986413	0.993421	0.98895	0.979644
330	0.971223	0.994565	0.993421	0.991318	0.984026
331	0.94964	0.98913	0.993421	0.987372	0.97673
332	0.94964	0.986413	0.994737	0.987372	0.976729
333	0.978417	0.991848	0.993421	0.991318	0.984031
334	0.942446	0.983696	0.993421	0.985004	0.972353
335	0.94964	0.986413	0.990789	0.985004	0.972361
336	0.971223	0.98913	0.992105	0.98895	0.979655
337	0.964029	0.98913	0.994737	0.98974	0.981098
338	0.964029	0.980978	0.994737	0.987372	0.97673
339	0.964029	0.98913	0.990789	0.987372	0.976747
340	0.94964	0.991848	0.993421	0.988161	0.978188
341	0.956835	0.991848	0.993421	0.98895	0.979651

342	0.971223	0.98913	0.989474	0.987372	0.97677
343	0.94964	0.983696	0.993421	0.985793	0.973813
344	0.964029	0.98913	0.993421	0.98895	0.979644
345	0.964029	0.991848	0.996053	0.991318	0.984011
346	0.94964	0.991848	0.990789	0.986582	0.975284
347	0.956835	0.991848	0.996053	0.990529	0.982558
348	0.964029	0.994565	0.990789	0.98895	0.979677
349	0.964029	0.98913	0.994737	0.98974	0.981098
350	0.956835	0.980978	0.992105	0.985004	0.972358
351	0.956835	0.980978	0.992105	0.985004	0.972357
352	0.94964	0.98913	0.992105	0.986582	0.975273
353	0.956835	0.994565	0.992105	0.98895	0.979664
354	0.956835	0.98913	0.992105	0.987372	0.976733
355	0.964029	0.994565	0.994737	0.991318	0.984017
356	0.964029	0.994565	0.993421	0.990529	0.982565
357	0.94964	0.991848	0.988158	0.985004	0.972397
358	0.978417	0.991848	0.994737	0.992107	0.985471
359	0.956835	0.994565	0.990789	0.988161	0.978222
360	0.971223	0.98913	0.993421	0.98974	0.981104
361	0.94964	0.980978	0.994737	0.985793	0.973817
362	0.964029	0.994565	0.996053	0.992107	0.985469
363	0.964029	0.991848	0.993421	0.98974	0.981104
364	0.942446	0.983696	0.992105	0.984215	0.970894
365	0.985612	0.994565	0.993421	0.992897	0.986943
366	0.94964	0.997283	0.996053	0.991318	0.984022
367	0.94964	0.98913	0.994737	0.988161	0.978185
368	0.971223	0.991848	0.992105	0.98974	0.98112
369	0.956835	0.994565	0.997368	0.992107	0.985474
370	0.964029	0.986413	0.992105	0.987372	0.976735
371	0.971223	0.986413	0.992105	0.988161	0.978198
372	0.971223	0.98913	0.996053	0.991318	0.984011
373	0.964029	0.991848	0.994737	0.990529	0.982555
374	0.94964	0.98913	0.992105	0.986582	0.975273
375	0.94964	0.991848	0.992105	0.987372	0.976736
376	0.964029	0.980978	0.992105	0.985793	0.97382
377	0.956835	0.991848	0.993421	0.98895	0.979645
378	0.964029	0.98913	0.996053	0.990529	0.982556
379	0.956835	0.98913	0.998684	0.991318	0.984024
380	0.956835	0.98913	0.990789	0.986582	0.975285
381	0.964029	0.991848	0.993421	0.98974	0.981106
382	0.956835	0.986413	0.994737	0.988161	0.978185
383	0.964029	0.994565	0.993421	0.990529	0.982565
384	0.935252	0.991848	0.993421	0.986582	0.975274

385	0.94964	0.994565	0.993421	0.98895	0.979649
386	0.956835	0.983696	0.994737	0.987372	0.976729
387	0.935252	0.991848	0.994737	0.987372	0.976739
388	0.935252	0.983696	0.989474	0.981847	0.966517
389	0.942446	0.98913	0.992105	0.985793	0.973825
390	0.964029	0.994565	0.996053	0.992107	0.985469
391	0.964029	0.994565	0.993421	0.990529	0.982565
392	0.956835	0.98913	0.993421	0.988161	0.978186
393	0.964029	0.986413	0.993421	0.988161	0.978186
394	0.964029	0.994565	0.990789	0.98895	0.979688
395	0.942446	0.98913	0.994737	0.987372	0.976733
396	0.964029	0.994565	0.993421	0.990529	0.982565
397	0.94964	0.986413	0.989474	0.984215	0.97091
398	0.956835	0.994565	0.993421	0.98974	0.981111
399	0.94964	0.991848	0.997368	0.990529	0.982565
400	0.94964	0.994565	0.993421	0.98895	0.979649
401	0.985612	0.991848	0.994737	0.992897	0.986929
402	0.94964	0.98913	0.993421	0.987372	0.976731
403	0.964029	0.983696	0.992105	0.986582	0.975273
404	0.978417	0.986413	0.993421	0.98974	0.98111
405	0.971223	0.991848	0.989474	0.988161	0.978236
406	0.964029	0.983696	0.997368	0.98974	0.981114
407	0.964029	0.983696	0.993421	0.987372	0.976729
408	0.971223	0.991848	0.992105	0.98974	0.98112
409	0.971223	0.986413	0.994737	0.98974	0.981098
410	0.985612	0.991848	0.992105	0.991318	0.984043
411	0.971223	0.98913	0.996053	0.991318	0.984012
412	0.956835	0.986413	0.994737	0.988161	0.978185
413	0.94964	0.986413	0.992105	0.985793	0.973812
414	0.956835	0.991848	0.992105	0.988161	0.978194
415	0.94964	0.991848	0.990789	0.986582	0.975288
416	0.971223	0.991848	0.994737	0.991318	0.984013
417	0.956835	0.980978	0.992105	0.985004	0.972357
418	0.985612	0.986413	0.990789	0.98895	0.979674
419	0.956835	0.983696	0.993421	0.986582	0.975269
420	0.94964	0.991848	0.994737	0.98895	0.979643
421	0.94964	0.98913	0.992105	0.986582	0.975273
422	0.942446	0.986413	0.993421	0.985793	0.973813
423	0.956835	0.991848	0.993421	0.98895	0.979649
424	0.942446	0.986413	0.993421	0.985793	0.973811
425	0.94964	0.991848	0.992105	0.987372	0.976736
426	0.956835	0.994565	0.993421	0.98974	0.981106
427	0.94964	0.997283	0.996053	0.991318	0.984016

428	0.942446	0.986413	0.992105	0.985004	0.972353
429	0.942446	0.983696	0.992105	0.984215	0.970892
430	0.971223	0.994565	0.992105	0.990529	0.982579
431	0.942446	0.994565	0.992105	0.987372	0.976741
432	0.964029	0.991848	0.994737	0.990529	0.982557
433	0.964029	0.98913	0.994737	0.98974	0.9811
434	0.964029	0.991848	0.992105	0.98895	0.979656
435	0.964029	0.991848	0.993421	0.98974	0.981104
436	0.978417	0.991848	0.993421	0.991318	0.984023
437	0.964029	0.997283	0.989474	0.98895	0.979722
438	0.942446	0.98913	0.993421	0.986582	0.97527
439	0.956835	0.980978	0.994737	0.986582	0.975273
440	0.971223	0.994565	0.996053	0.992897	0.986924
441	0.964029	0.994565	0.992105	0.98974	0.981119
442	0.964029	0.983696	0.996053	0.98895	0.979648
443	0.971223	0.98913	0.992105	0.98895	0.979655
444	0.956835	0.98913	0.990789	0.986582	0.975284
445	0.935252	0.98913	0.994737	0.986582	0.975275
446	0.942446	0.983696	0.994737	0.985793	0.973818
447	0.94964	0.991848	0.994737	0.98895	0.979643
448	0.964029	0.986413	0.996053	0.98974	0.981103
449	0.971223	0.991848	0.993421	0.990529	0.982565
450	0.964029	0.98913	0.992105	0.988161	0.978195
451	0.971223	0.997283	0.990789	0.990529	0.982606
452	0.964029	0.98913	0.993421	0.98895	0.979645
453	0.94964	0.98913	0.992105	0.986582	0.975277
454	0.971223	0.98913	0.992105	0.98895	0.979655
455	0.94964	0.991848	0.996053	0.98974	0.981105
456	0.956835	0.994565	0.993421	0.98974	0.981106
457	0.942446	0.991848	0.988158	0.984215	0.970931
458	0.94964	0.994565	0.990789	0.987372	0.976754
459	0.964029	0.991848	0.993421	0.98974	0.981104
460	0.971223	0.980978	0.994737	0.988161	0.978192
461	0.942446	0.994565	0.994737	0.98895	0.979647
462	0.964029	0.986413	0.994737	0.98895	0.979646
463	0.978417	0.991848	0.992105	0.990529	0.982579
464	0.94964	0.991848	0.992105	0.987372	0.976736
465	0.971223	0.98913	0.994737	0.990529	0.982555
466	0.94964	0.991848	0.994737	0.98895	0.979647
467	0.942446	0.994565	0.992105	0.987372	0.976741
468	0.956835	0.991848	0.994737	0.98974	0.981099
469	0.964029	0.983696	0.990789	0.985793	0.973822
470	0.94964	0.994565	0.993421	0.98895	0.979655

471	0.956835	0.98913	0.988158	0.985004	0.972397
472	0.971223	0.991848	0.993421	0.990529	0.982564
473	0.964029	0.991848	0.990789	0.988161	0.978212
474	0.964029	0.997283	0.993421	0.991318	0.984026
475	0.935252	0.991848	0.993421	0.986582	0.975274
476	0.942446	0.994565	0.993421	0.988161	0.978192
477	0.94964	0.991848	0.993421	0.988161	0.978188
478	0.942446	0.98913	0.996053	0.988161	0.978196
479	0.971223	0.994565	0.993421	0.991318	0.984036
480	0.971223	0.98913	0.993421	0.98974	0.981104
481	0.94964	0.983696	0.996053	0.987372	0.976739
482	0.964029	0.997283	0.990789	0.98974	0.981144
483	0.956835	0.983696	0.993421	0.986582	0.975269
484	0.964029	0.994565	0.992105	0.98974	0.981117
485	0.942446	0.98913	0.992105	0.985793	0.973814
486	0.978417	0.98913	0.992105	0.98974	0.98112
487	0.964029	0.98913	0.996053	0.990529	0.982556
488	0.935252	0.983696	0.996053	0.985793	0.973832
489	0.94964	0.991848	0.996053	0.98974	0.981105
490	0.964029	0.98913	0.996053	0.990529	0.982559
491	0.956835	0.986413	0.986842	0.983425	0.96949
492	0.956835	0.986413	0.993421	0.987372	0.976727
493	0.956835	0.980978	0.993421	0.985793	0.973813
494	0.964029	0.991848	0.994737	0.990529	0.982555
495	0.964029	0.986413	0.992105	0.987372	0.976733
496	0.964029	0.98913	0.989474	0.986582	0.975304
497	0.942446	0.994565	0.994737	0.98895	0.979647
498	0.956835	0.978261	0.994737	0.985793	0.973817
499	0.94964	0.983696	0.997368	0.988161	0.978209
500	0.94964	0.980978	0.990789	0.983425	0.969437
501	0.956835	0.98913	0.993421	0.988161	0.978189
502	0.971223	0.98913	0.990789	0.988161	0.978222
503	0.94964	0.986413	0.994737	0.987372	0.97673
504	0.94964	0.98913	0.993421	0.987372	0.976727
505	0.956835	0.986413	0.997368	0.98974	0.981112
506	0.964029	0.98913	0.994737	0.98974	0.981101
507	0.964029	0.991848	0.993421	0.98974	0.981104
508	0.978417	0.983696	0.992105	0.988161	0.978198
509	0.956835	0.98913	0.994737	0.98895	0.979645
510	0.964029	0.991848	0.993421	0.98974	0.981104
511	0.94964	0.986413	0.994737	0.987372	0.97673
512	0.956835	0.991848	0.996053	0.990529	0.982556
513	0.94964	0.991848	0.989474	0.985793	0.973839

514	0.94964	0.986413	0.990789	0.985004	0.972362
515	0.964029	0.986413	0.992105	0.987372	0.976733
516	0.942446	0.991848	0.992105	0.986582	0.975283
517	0.956835	0.983696	0.992105	0.985793	0.973812
518	0.964029	0.991848	0.994737	0.990529	0.982562
519	0.964029	0.994565	0.996053	0.992107	0.985469
520	0.942446	0.994565	0.997368	0.990529	0.982572
521	0.971223	0.986413	0.993421	0.98895	0.979649
522	0.956835	0.991848	0.994737	0.98974	0.981102
523	0.956835	0.986413	0.993421	0.987372	0.97673
524	0.956835	0.991848	0.996053	0.990529	0.982558
525	0.94964	0.986413	0.993421	0.986582	0.975269
526	0.935252	0.98913	0.992105	0.985004	0.972356
527	0.971223	0.991848	0.996053	0.992107	0.985466
528	0.942446	0.986413	0.990789	0.984215	0.970898
529	0.964029	0.98913	0.993421	0.98895	0.979645
530	0.971223	0.997283	0.993421	0.992107	0.985485
531	0.956835	0.98913	0.996053	0.98974	0.981102
532	0.956835	0.98913	0.992105	0.987372	0.976739
533	0.935252	0.997283	0.997368	0.990529	0.982572
534	0.956835	0.991848	0.984211	0.983425	0.969568
535	0.956835	0.994565	0.990789	0.988161	0.978211
536	0.964029	0.986413	0.992105	0.987372	0.976733
537	0.942446	0.994565	0.993421	0.988161	0.978192
538	0.94964	0.994565	0.992105	0.988161	0.9782
539	0.94964	0.991848	0.992105	0.987372	0.976736
540	0.942446	0.997283	0.994737	0.98974	0.981114
541	0.956835	0.98913	0.993421	0.988161	0.978186
542	0.956835	0.991848	0.993421	0.98895	0.979649
543	0.942446	0.991848	0.996053	0.98895	0.979649
544	0.956835	0.994565	0.998684	0.992897	0.986936
545	0.94964	0.991848	0.994737	0.98895	0.979645
546	0.978417	0.983696	0.992105	0.988161	0.978198
547	0.956835	0.994565	0.994737	0.990529	0.982562
548	0.964029	0.986413	0.992105	0.987372	0.976733
549	0.956835	0.986413	0.990789	0.985793	0.973822
550	0.94964	0.997283	0.996053	0.991318	0.984022
551	0.964029	0.991848	0.994737	0.990529	0.982557
552	0.964029	0.991848	0.993421	0.98974	0.981104
553	0.971223	0.983696	0.997368	0.990529	0.982567
554	0.971223	0.994565	0.994737	0.992107	0.98548
555	0.94964	0.986413	0.990789	0.985004	0.972359
556	0.942446	0.991848	0.992105	0.986582	0.975272

557	0.956835	0.994565	0.996053	0.991318	0.984018
558	0.964029	0.994565	0.994737	0.991318	0.984017
559	0.942446	0.98913	0.990789	0.985004	0.972359
560	0.94964	0.991848	0.989474	0.985793	0.973839
561	0.94964	0.98913	0.994737	0.988161	0.978185
562	0.964029	0.991848	0.994737	0.990529	0.982555
563	0.942446	0.986413	0.992105	0.985004	0.972352
564	0.94964	0.980978	0.994737	0.985793	0.973823
565	0.985612	0.991848	0.993421	0.992107	0.985494
566	0.964029	0.991848	0.994737	0.990529	0.982555
567	0.935252	0.983696	0.990789	0.982636	0.967974
568	0.978417	0.983696	0.993421	0.98895	0.979646
569	0.94964	0.991848	0.994737	0.98895	0.979647
570	0.964029	0.98913	0.994737	0.98974	0.981098
571	0.964029	0.994565	0.996053	0.992107	0.985469
572	0.94964	0.980978	0.994737	0.985793	0.973819
573	0.956835	0.98913	0.996053	0.98974	0.981102
574	0.956835	0.986413	0.994737	0.988161	0.978185
575	0.971223	0.98913	0.993421	0.98974	0.981104
576	0.935252	0.991848	0.992105	0.985793	0.973819
577	0.94964	0.986413	0.997368	0.98895	0.979661
578	0.920863	0.991848	0.990789	0.983425	0.969442
579	0.956835	0.986413	0.990789	0.985793	0.97383
580	0.94964	0.986413	0.992105	0.985793	0.973816
581	0.956835	0.986413	0.992105	0.986582	0.975272
582	0.94964	0.994565	0.996053	0.990529	0.982563
583	0.956835	0.98913	0.993421	0.988161	0.978186
584	0.928058	0.994565	0.996053	0.988161	0.978201
585	0.94964	0.991848	0.994737	0.98895	0.979649
586	0.942446	0.994565	0.994737	0.98895	0.979647
587	0.964029	0.983696	0.990789	0.985793	0.973825
588	0.942446	0.991848	0.994737	0.988161	0.978188
589	0.94964	0.994565	0.993421	0.98895	0.979649
590	0.942446	0.986413	0.996053	0.987372	0.97674
591	0.94964	0.98913	0.993421	0.987372	0.976727
592	0.971223	0.98913	0.996053	0.991318	0.984011
593	0.956835	0.98913	0.994737	0.98895	0.979642
594	0.956835	0.994565	0.994737	0.990529	0.982562
595	0.971223	0.994565	0.992105	0.990529	0.98258
596	0.964029	0.983696	0.994737	0.988161	0.978185
597	0.94964	0.991848	0.996053	0.98974	0.981102
598	0.956835	0.997283	0.994737	0.991318	0.984023
599	0.942446	0.986413	0.994737	0.986582	0.975275

600	0.978417	0.98913	0.996053	0.992107	0.985468
601	0.956835	0.991848	0.994737	0.98974	0.981099
602	0.971223	0.983696	0.996053	0.98974	0.981108
603	0.94964	0.994565	0.993421	0.98895	0.979651
604	0.94964	0.98913	0.994737	0.988161	0.978185
605	0.971223	0.991848	0.992105	0.98974	0.98112
606	0.956835	0.991848	0.992105	0.988161	0.978196
607	0.956835	0.98913	0.996053	0.98974	0.981102
608	0.964029	0.98913	0.993421	0.98895	0.979644
609	0.964029	0.98913	0.996053	0.990529	0.982556
610	0.956835	0.986413	0.994737	0.988161	0.978185
611	0.94964	0.98913	0.996053	0.98895	0.979647
612	0.94964	0.986413	0.996053	0.988161	0.978193
613	0.942446	0.994565	0.994737	0.98895	0.979647
614	0.971223	0.991848	0.994737	0.991318	0.984013
615	0.942446	0.994565	0.993421	0.988161	0.978192
616	0.964029	0.98913	0.992105	0.988161	0.978196
617	0.964029	0.98913	0.994737	0.98974	0.981099
618	0.956835	0.98913	0.994737	0.98895	0.979642
619	0.964029	0.986413	0.993421	0.988161	0.978186
620	0.942446	0.98913	0.996053	0.988161	0.978196
621	0.971223	0.980978	0.994737	0.988161	0.978192
622	0.956835	0.983696	0.992105	0.985793	0.973812
623	0.94964	0.98913	0.990789	0.985793	0.973827
624	0.978417	0.991848	0.990789	0.98974	0.981139
625	0.956835	0.991848	0.993421	0.98895	0.979644
626	0.94964	0.991848	0.990789	0.986582	0.975285
627	0.94964	0.991848	0.997368	0.990529	0.982568
628	0.964029	0.991848	0.992105	0.98895	0.979656
629	0.956835	0.991848	0.993421	0.98895	0.979644
630	0.964029	0.994565	0.996053	0.992107	0.985469
631	0.971223	0.997283	0.992105	0.991318	0.984049
632	0.956835	0.994565	0.994737	0.990529	0.982562
633	0.942446	0.986413	0.997368	0.988161	0.978209
634	0.956835	0.994565	0.992105	0.98895	0.979659
635	0.94964	0.991848	0.994737	0.98895	0.979643
636	0.956835	0.991848	0.994737	0.98974	0.981102
637	0.956835	0.991848	0.994737	0.98974	0.981102
638	0.942446	0.991848	0.993421	0.987372	0.97673
639	0.956835	0.997283	0.993421	0.990529	0.982575
640	0.971223	0.986413	0.992105	0.988161	0.978195
641	0.956835	0.98913	0.994737	0.98895	0.979642
642	0.942446	0.98913	0.992105	0.985793	0.97382

643	0.956835	0.98913	0.993421	0.988161	0.978189
644	0.94964	0.986413	0.996053	0.988161	0.978193
645	0.935252	0.991848	0.994737	0.987372	0.976739
646	0.94964	0.986413	0.994737	0.987372	0.97673
647	0.971223	0.991848	0.996053	0.992107	0.985467
648	0.94964	0.991848	0.996053	0.98974	0.981105
649	0.964029	0.991848	0.993421	0.98974	0.981104
650	0.971223	0.986413	0.992105	0.988161	0.978195
651	0.94964	0.991848	0.990789	0.986582	0.975285
652	0.956835	0.991848	0.994737	0.98974	0.981102
653	0.971223	0.98913	0.993421	0.98974	0.981104
654	0.956835	0.991848	0.993421	0.98895	0.979645
655	0.956835	0.986413	0.996053	0.98895	0.979647
656	0.964029	0.98913	0.994737	0.98974	0.981098
657	0.964029	0.986413	0.993421	0.988161	0.978186
658	0.94964	0.991848	0.996053	0.98974	0.981105
659	0.964029	0.98913	0.993421	0.98895	0.979644
660	0.94964	0.991848	0.990789	0.986582	0.975288
661	0.971223	0.98913	0.992105	0.98895	0.979661
662	0.94964	0.98913	0.990789	0.985793	0.973829
663	0.956835	0.98913	0.990789	0.986582	0.975285
664	0.964029	0.983696	0.990789	0.985793	0.973822
665	0.964029	0.978261	0.992105	0.985004	0.972363
666	0.964029	0.98913	0.993421	0.98895	0.979644
667	0.942446	0.986413	0.993421	0.985793	0.973811
668	0.964029	0.986413	0.994737	0.98895	0.979642
669	0.971223	0.986413	0.994737	0.98974	0.981098
670	0.942446	0.98913	0.994737	0.987372	0.976733
671	0.94964	0.98913	0.993421	0.987372	0.97673
672	0.971223	0.98913	0.996053	0.991318	0.984015
673	0.956835	0.98913	0.996053	0.98974	0.981101
674	0.964029	0.994565	0.996053	0.992107	0.985469
675	0.956835	0.994565	0.992105	0.98895	0.979659
676	0.942446	0.983696	0.992105	0.984215	0.970894
677	0.956835	0.98913	0.994737	0.98895	0.979643
678	0.942446	0.98913	0.997368	0.98895	0.979663
679	0.942446	0.991848	0.992105	0.986582	0.975273
680	0.956835	0.98913	0.988158	0.985004	0.972397
681	0.956835	1	0.990789	0.98974	0.981144
682	0.928058	0.991848	0.997368	0.988161	0.978218
683	0.94964	0.98913	0.996053	0.98895	0.979647
684	0.956835	0.98913	0.994737	0.98895	0.979642
685	0.935252	0.986413	0.992105	0.984215	0.970892

686	0.964029	0.983696	0.990789	0.985793	0.973822
687	0.935252	0.991848	0.994737	0.987372	0.976739
688	0.971223	0.991848	0.994737	0.991318	0.984014
689	0.935252	0.991848	0.996053	0.988161	0.978196
690	0.956835	0.991848	0.994737	0.98974	0.981098
691	0.971223	0.98913	0.992105	0.98895	0.979657
692	0.971223	0.98913	0.992105	0.98895	0.979655
693	0.94964	0.994565	0.994737	0.98974	0.981102
694	0.94964	0.98913	0.994737	0.988161	0.978185
695	0.956835	0.994565	0.994737	0.990529	0.982562
696	0.971223	0.98913	0.993421	0.98974	0.981106
697	0.956835	0.991848	0.992105	0.988161	0.978196
698	0.971223	0.986413	0.992105	0.988161	0.978198
699	0.964029	0.991848	0.992105	0.98895	0.979664
700	0.942446	0.98913	0.990789	0.985004	0.972362
701	0.956835	0.983696	0.996053	0.988161	0.978193
702	0.964029	0.98913	0.992105	0.988161	0.978196
703	0.942446	0.98913	0.996053	0.988161	0.978196
704	0.94964	0.983696	0.996053	0.987372	0.976739
705	0.94964	0.994565	0.994737	0.98974	0.981099
706	0.964029	0.98913	0.997368	0.991318	0.984021
707	0.964029	0.991848	0.992105	0.98895	0.979656
708	0.971223	0.991848	0.993421	0.990529	0.982565
709	0.956835	0.994565	0.996053	0.991318	0.984012
710	0.964029	0.986413	0.998684	0.991318	0.984032
711	0.935252	0.994565	0.997368	0.98974	0.981123
712	0.956835	0.997283	0.990789	0.98895	0.979677
713	0.94964	0.98913	0.993421	0.987372	0.976727
714	0.964029	0.98913	0.989474	0.986582	0.975305
715	0.964029	0.983696	0.994737	0.988161	0.978188
716	0.94964	0.991848	0.994737	0.98895	0.979643
717	0.942446	0.991848	0.990789	0.985793	0.973823
718	0.956835	0.991848	0.994737	0.98974	0.981102
719	0.94964	0.997283	0.993421	0.98974	0.981111
720	0.956835	0.983696	0.997368	0.98895	0.979664
721	0.935252	0.98913	0.994737	0.986582	0.975275
722	0.964029	0.994565	0.992105	0.98974	0.981119
723	0.94964	0.991848	0.993421	0.988161	0.978188
724	0.964029	0.991848	0.992105	0.98895	0.979655
725	0.942446	0.986413	0.996053	0.987372	0.97674
726	0.964029	0.991848	0.989474	0.987372	0.97677
727	0.956835	0.994565	0.993421	0.98974	0.981104
728	0.971223	0.991848	0.994737	0.991318	0.984013

729	0.971223	0.991848	0.996053	0.992107	0.985467
730	0.956835	0.986413	0.993421	0.987372	0.976729
731	0.956835	0.991848	0.994737	0.98974	0.981099
732	0.942446	0.98913	0.994737	0.987372	0.976733
733	0.94964	0.980978	0.990789	0.983425	0.969435
734	0.928058	0.991848	0.994737	0.986582	0.975285
735	0.956835	0.986413	0.992105	0.986582	0.975273
736	0.935252	0.983696	0.993421	0.984215	0.970897
737	0.971223	0.98913	0.993421	0.98974	0.981104
738	0.956835	0.991848	0.996053	0.990529	0.982558
739	0.964029	0.994565	0.992105	0.98974	0.981119
740	0.94964	0.991848	0.993421	0.988161	0.978192
741	0.978417	0.991848	0.994737	0.992107	0.985471
742	0.964029	0.98913	0.992105	0.988161	0.978198
743	0.964029	0.986413	0.989474	0.985793	0.97384
744	0.94964	0.975543	0.994737	0.984215	0.970909
745	0.971223	0.991848	0.996053	0.992107	0.985467
746	0.978417	0.991848	0.993421	0.991318	0.984023
747	0.956835	0.98913	0.998684	0.991318	0.984032
748	0.942446	0.98913	0.997368	0.98895	0.979663
749	0.964029	0.986413	0.990789	0.986582	0.975286
750	0.964029	0.98913	0.993421	0.98895	0.979644
751	0.964029	0.986413	0.994737	0.98895	0.979646
752	0.920863	0.98913	0.992105	0.983425	0.969435
753	0.928058	0.98913	0.996053	0.986582	0.975292
754	0.94964	0.986413	0.994737	0.987372	0.97673
755	0.942446	0.986413	0.990789	0.984215	0.970898
756	0.94964	0.986413	0.990789	0.985004	0.972365
757	0.94964	0.994565	0.996053	0.990529	0.982563
758	0.942446	0.991848	0.990789	0.985793	0.973823
759	0.94964	0.98913	0.994737	0.988161	0.978185
760	0.956835	0.98913	0.994737	0.98895	0.979642
761	0.942446	0.986413	0.997368	0.988161	0.978209
762	0.94964	0.997283	0.996053	0.991318	0.984016
763	0.942446	0.994565	0.994737	0.98895	0.979653
764	0.964029	0.986413	0.994737	0.98895	0.979642
765	0.94964	0.98913	0.994737	0.988161	0.978185
766	0.94964	0.986413	0.996053	0.988161	0.978193
767	0.94964	0.994565	0.992105	0.988161	0.9782
768	0.94964	0.986413	0.990789	0.985004	0.972359
769	0.935252	0.991848	0.993421	0.986582	0.97527
770	0.94964	0.98913	0.993421	0.987372	0.97673
771	0.94964	0.983696	0.990789	0.984215	0.970898

772	0.971223	0.98913	0.992105	0.98895	0.979655
773	0.935252	0.991848	0.990789	0.985004	0.972359
774	0.956835	0.983696	0.997368	0.98895	0.979664
775	0.956835	0.983696	0.994737	0.987372	0.976734
776	0.935252	0.986413	0.993421	0.985004	0.972354
777	0.935252	0.98913	0.996053	0.987372	0.976744
778	0.942446	0.98913	0.990789	0.985004	0.972362
779	0.928058	0.98913	0.992105	0.984215	0.970892
780	0.956835	0.98913	0.993421	0.988161	0.978188
781	0.942446	0.98913	0.993421	0.986582	0.975274
782	0.94964	0.994565	0.996053	0.990529	0.982558
783	0.94964	0.98913	0.993421	0.987372	0.976727
784	0.94964	0.983696	0.996053	0.987372	0.976739
785	0.956835	0.986413	0.990789	0.985793	0.973825
786	0.956835	0.991848	0.994737	0.98974	0.981105
787	0.942446	0.98913	0.996053	0.988161	0.978193
788	0.956835	0.994565	0.994737	0.990529	0.982557
789	0.942446	0.98913	0.994737	0.987372	0.976727
790	0.956835	0.986413	0.996053	0.98895	0.979647
791	0.935252	0.986413	0.996053	0.986582	0.975288
792	0.964029	0.986413	0.992105	0.987372	0.97674
793	0.971223	0.986413	0.996053	0.990529	0.982556
794	0.94964	0.978261	0.992105	0.983425	0.969439
795	0.942446	0.994565	0.993421	0.988161	0.978192
796	0.935252	0.983696	0.992105	0.983425	0.969434
797	0.942446	0.991848	0.996053	0.98895	0.979649
798	0.935252	0.98913	0.997368	0.988161	0.978212
799	0.94964	0.98913	0.997368	0.98974	0.981113
800	0.964029	0.986413	0.990789	0.986582	0.975284
801	0.956835	0.98913	0.993421	0.988161	0.978186
802	0.94964	0.986413	0.994737	0.987372	0.976729
803	0.971223	0.991848	0.994737	0.991318	0.984014
804	0.964029	0.98913	0.997368	0.991318	0.984017
805	0.935252	0.991848	0.993421	0.986582	0.975274
806	0.928058	0.994565	0.997368	0.98895	0.979667
807	0.942446	0.991848	0.993421	0.987372	0.97673
808	0.935252	0.98913	0.990789	0.984215	0.970897
809	0.942446	0.991848	0.988158	0.984215	0.97093
810	0.942446	0.98913	0.993421	0.986582	0.97527
811	0.94964	0.994565	0.997368	0.991318	0.984021
812	0.928058	0.986413	0.993421	0.984215	0.970897
813	0.935252	0.994565	0.994737	0.988161	0.978192
814	0.978417	0.991848	0.993421	0.991318	0.984031

815	0.964029	0.994565	0.994737	0.991318	0.984014
816	0.956835	0.983696	0.994737	0.987372	0.976734
817	0.94964	0.994565	0.996053	0.990529	0.982558
818	0.942446	0.986413	0.993421	0.985793	0.973811
819	0.942446	0.991848	0.994737	0.988161	0.978188
820	0.942446	0.991848	0.994737	0.988161	0.978192
821	0.94964	0.991848	0.997368	0.990529	0.982568
822	0.956835	0.991848	0.993421	0.98895	0.979649
823	0.956835	0.983696	0.994737	0.987372	0.976729
824	0.942446	0.98913	0.992105	0.985793	0.973814
825	0.942446	0.983696	0.997368	0.987372	0.976747
826	0.964029	0.98913	0.996053	0.990529	0.982556
827	0.942446	0.986413	0.994737	0.986582	0.975273
828	0.956835	0.994565	0.994737	0.990529	0.982557
829	0.978417	0.991848	0.990789	0.98974	0.981141
830	0.964029	0.983696	0.992105	0.986582	0.975277
831	0.94964	0.991848	0.993421	0.988161	0.978188
832	0.956835	0.98913	0.994737	0.98895	0.979642
833	0.971223	0.983696	0.992105	0.987372	0.976746
834	0.942446	0.991848	0.994737	0.988161	0.978188
835	0.935252	0.997283	0.993421	0.988161	0.978199
836	0.956835	0.991848	0.993421	0.98895	0.979644
837	0.971223	0.991848	0.994737	0.991318	0.984014
838	0.94964	0.994565	0.996053	0.990529	0.982564
839	0.956835	0.994565	0.990789	0.988161	0.978211
840	0.964029	0.994565	0.992105	0.98974	0.981119
841	0.971223	0.98913	0.992105	0.98895	0.979655
842	0.964029	0.991848	0.994737	0.990529	0.982555
843	0.920863	0.991848	0.996053	0.986582	0.975292
844	0.935252	0.991848	0.992105	0.985793	0.973814
845	0.942446	0.997283	0.992105	0.988161	0.978196
846	0.956835	0.983696	0.994737	0.987372	0.976727
847	0.956835	0.994565	0.994737	0.990529	0.982555
848	0.94964	0.98913	0.993421	0.987372	0.976727
849	0.94964	0.994565	0.996053	0.990529	0.982558
850	0.920863	0.986413	0.992105	0.982636	0.967973
851	0.935252	0.98913	0.992105	0.985004	0.972352
852	0.964029	0.994565	0.989474	0.988161	0.978235
853	0.94964	0.986413	0.992105	0.985793	0.973812
854	0.964029	0.983696	0.996053	0.98895	0.979656
855	0.94964	0.98913	0.994737	0.988161	0.978185
856	0.971223	0.991848	0.994737	0.991318	0.984014
857	0.956835	0.986413	0.996053	0.98895	0.979647

858	0.942446	0.986413	0.997368	0.988161	0.978209
859	0.964029	0.994565	0.994737	0.991318	0.984014
860	0.942446	0.994565	0.994737	0.98895	0.979653
861	0.935252	0.98913	0.990789	0.984215	0.970897
862	0.942446	0.98913	0.994737	0.987372	0.97673
863	0.935252	0.994565	0.990789	0.985793	0.973827
864	0.935252	0.983696	0.996053	0.985793	0.973833
865	0.956835	0.997283	0.996053	0.992107	0.985469
866	0.956835	0.986413	0.994737	0.988161	0.978185
867	0.964029	0.983696	0.996053	0.98895	0.979648
868	0.94964	0.991848	0.993421	0.988161	0.978186
869	0.94964	0.991848	0.990789	0.986582	0.975285
870	0.964029	0.983696	0.992105	0.986582	0.975273
871	0.971223	0.997283	0.996053	0.993686	0.988382
872	0.964029	0.986413	0.994737	0.98895	0.979642
873	0.964029	0.991848	0.997368	0.992107	0.985471
874	0.94964	0.986413	0.996053	0.988161	0.978193
875	0.94964	0.994565	0.997368	0.991318	0.984023
876	0.94964	0.98913	0.998684	0.990529	0.982582
877	0.971223	0.98913	0.994737	0.990529	0.982555
878	0.956835	0.98913	0.996053	0.98974	0.981102
879	0.956835	0.98913	0.992105	0.987372	0.976733
880	0.942446	0.991848	0.994737	0.988161	0.978188
881	0.971223	0.991848	0.993421	0.990529	0.982565
882	0.964029	0.98913	0.994737	0.98974	0.981098
883	0.94964	0.994565	0.994737	0.98974	0.981107
884	0.971223	0.986413	0.996053	0.990529	0.982559
885	0.94964	0.997283	0.996053	0.991318	0.984016
886	0.942446	0.983696	0.998684	0.988161	0.978233
887	0.964029	0.980978	0.994737	0.987372	0.976735
888	0.94964	0.991848	0.996053	0.98974	0.981105
889	0.935252	0.98913	0.994737	0.986582	0.975275
890	0.985612	0.98913	0.998684	0.994475	0.989833
891	0.964029	0.98913	0.990789	0.987372	0.976747
892	0.964029	0.986413	0.994737	0.98895	0.979642
893	0.956835	0.991848	0.994737	0.98974	0.981102
894	0.964029	0.994565	0.993421	0.990529	0.982565
895	0.935252	0.994565	0.994737	0.988161	0.978192
896	0.971223	0.986413	0.994737	0.98974	0.9811
897	0.964029	0.983696	0.992105	0.986582	0.975273
898	0.964029	0.983696	0.994737	0.988161	0.978192
899	0.94964	0.994565	0.997368	0.991318	0.984023
900	0.971223	0.983696	0.994737	0.98895	0.979646

901	0.971223	0.986413	0.992105	0.988161	0.978195
902	0.956835	0.986413	0.997368	0.98974	0.981112
903	0.971223	0.997283	0.996053	0.993686	0.988382
904	0.942446	0.986413	0.994737	0.986582	0.975275
905	0.956835	0.991848	0.994737	0.98974	0.981099
906	0.964029	0.98913	0.993421	0.98895	0.979645
907	0.94964	0.98913	0.996053	0.98895	0.979649
908	0.964029	0.994565	0.997368	0.992897	0.986925
909	0.942446	0.994565	0.996053	0.98974	0.981111
910	0.964029	0.986413	0.994737	0.98895	0.979642
911	0.942446	0.991848	0.994737	0.988161	0.978188
912	0.964029	0.98913	0.994737	0.98974	0.981099
913	0.971223	0.98913	0.989474	0.987372	0.97677
914	0.94964	0.98913	0.993421	0.987372	0.97673
915	0.94964	0.98913	0.992105	0.986582	0.975272
916	0.942446	0.98913	0.994737	0.987372	0.976733
917	0.964029	0.983696	0.993421	0.987372	0.976733
918	0.94964	0.986413	0.990789	0.985004	0.972359
919	0.956835	0.991848	0.998684	0.992107	0.985483
920	0.956835	0.991848	0.996053	0.990529	0.982556
921	0.964029	0.986413	0.994737	0.98895	0.979642
922	0.971223	0.994565	0.994737	0.992107	0.985473
923	0.956835	0.991848	0.996053	0.990529	0.982558
924	0.964029	0.991848	0.996053	0.991318	0.984012
925	0.94964	0.98913	0.994737	0.988161	0.978189
926	0.942446	0.98913	0.997368	0.98895	0.979663
927	0.978417	0.994565	0.990789	0.990529	0.982602
928	0.964029	0.986413	0.994737	0.98895	0.979646
929	0.971223	0.983696	0.993421	0.988161	0.978189
930	0.94964	0.994565	0.992105	0.988161	0.9782
931	0.964029	0.991848	0.996053	0.991318	0.984012
932	0.964029	0.994565	0.993421	0.990529	0.982563
933	0.964029	0.986413	0.997368	0.990529	0.982565
934	0.94964	0.986413	0.994737	0.987372	0.97673
935	0.971223	0.991848	0.998684	0.993686	0.98838
936	0.971223	0.991848	0.997368	0.992897	0.986923
937	0.971223	0.991848	0.994737	0.991318	0.984013
938	0.94964	0.991848	0.996053	0.98974	0.981102
939	0.942446	0.991848	0.996053	0.98895	0.979649
940	0.94964	0.991848	0.996053	0.98974	0.981105
941	0.935252	0.991848	0.994737	0.987372	0.97674
942	0.964029	0.991848	0.993421	0.98974	0.981104
943	0.94964	0.983696	0.994737	0.986582	0.975273

944	0.94964	0.991848	0.996053	0.98974	0.981102
945	0.94964	0.98913	0.997368	0.98974	0.981113
946	0.956835	0.98913	0.992105	0.987372	0.976733
947	0.964029	0.991848	0.997368	0.992107	0.985471
948	0.94964	0.991848	0.993421	0.988161	0.978186
949	0.964029	0.994565	0.996053	0.992107	0.985469
950	0.964029	0.991848	0.994737	0.990529	0.982555
951	0.964029	0.986413	0.996053	0.98974	0.981101
952	0.971223	0.98913	0.993421	0.98974	0.981106
953	0.94964	0.98913	0.992105	0.986582	0.975272
954	0.956835	0.991848	0.997368	0.991318	0.984019
955	0.964029	0.986413	0.990789	0.986582	0.975296
956	0.964029	0.991848	0.997368	0.992107	0.98547
957	0.964029	0.986413	0.993421	0.988161	0.978186
958	0.964029	0.98913	0.994737	0.98974	0.981098
959	0.964029	0.98913	0.994737	0.98974	0.9811
960	0.964029	0.991848	0.993421	0.98974	0.981106
961	0.971223	0.991848	0.993421	0.990529	0.982563
962	0.935252	0.986413	0.997368	0.987372	0.976757
963	0.94964	0.98913	0.994737	0.988161	0.978185
964	0.942446	0.986413	0.992105	0.985004	0.972353
965	0.942446	0.98913	0.996053	0.988161	0.978196
966	0.94964	0.986413	0.998684	0.98974	0.981132
967	0.956835	0.991848	0.997368	0.991318	0.984019
968	0.942446	0.986413	0.996053	0.987372	0.97673
969	0.964029	0.994565	0.994737	0.991318	0.984014
970	0.964029	0.986413	0.992105	0.987372	0.976735
971	0.956835	0.991848	0.992105	0.988161	0.978204
972	0.978417	0.986413	0.993421	0.98974	0.98111
973	0.964029	0.994565	0.990789	0.98895	0.979674
974	0.94964	0.991848	0.996053	0.98974	0.981105
975	0.964029	0.98913	0.994737	0.98974	0.981098
976	0.94964	0.994565	0.992105	0.988161	0.978194
977	0.964029	0.991848	0.993421	0.98974	0.981104
978	0.971223	0.991848	0.997368	0.992897	0.986923
979	0.964029	0.98913	0.993421	0.98895	0.979644
980	0.971223	0.98913	0.994737	0.990529	0.982556
981	0.942446	0.986413	0.996053	0.987372	0.976739
982	0.956835	0.994565	0.996053	0.991318	0.984016
983	0.971223	0.986413	1	0.992897	0.986953
984	0.94964	0.994565	0.992105	0.988161	0.9782
985	0.94964	0.991848	0.994737	0.98895	0.979647
986	0.956835	0.991848	0.994737	0.98974	0.981102

987	0.978417	0.991848	0.993421	0.991318	0.984023
988	0.956835	0.994565	0.996053	0.991318	0.984016
989	0.985612	0.980978	0.994737	0.98974	0.98112
990	0.942446	0.98913	0.994737	0.987372	0.976733
991	0.942446	0.991848	0.992105	0.986582	0.975273
992	0.964029	0.98913	0.996053	0.990529	0.982556
993	0.964029	0.994565	0.996053	0.992107	0.985469
994	0.971223	0.98913	0.993421	0.98974	0.981106
995	0.956835	0.98913	0.996053	0.98974	0.981102
996	0.971223	0.994565	0.997368	0.993686	0.988377
997	0.94964	0.983696	0.997368	0.988161	0.97821
998	0.956835	0.991848	0.996053	0.990529	0.982558
999	0.956835	0.994565	0.994737	0.990529	0.982557
1000	0.94964	0.994565	0.996053	0.990529	0.982563
